# ﻿An updated checklist of liverworts and hornworts of Malaysia

**DOI:** 10.3897/phytokeys.199.76693

**Published:** 2022-06-06

**Authors:** Gaik Ee Lee, S. Robbert Gradstein, Elizabeth Pesiu, Nik Norhazrina

**Affiliations:** 1 Faculty of Science and Marine Environment, 21030 Kuala Nerus, Universiti Malaysia Terengganu, Terengganu, Malaysia; 2 Institute of Tropical Biodiversity and Sustainable Development, 21030 Kuala Nerus, Universiti Malaysia Terengganu, Terengganu, Malaysia; 3 Meise Botanic Garden, 1860 Meise, Belgium; 4 Muséum National d’Histoire Naturelle, Institute de Systematique, Évolution, Biodiversité (UMR 7205), 75005 Paris, France; 5 Department of Biological Sciences and Biotechnology, Faculty of Science and Technology, Universiti Kebangsaan Malaysia, Bangi, 43600 Selangor, Malaysia

**Keywords:** Bryophytes, checklist, hornworts, liverworts, Malaysia, species

## Abstract

An updated checklist of the liverworts and hornworts of Malaysia accepts 773 species and 31 infraspecific taxa of liverworts, in 120 genera and 40 families, and 7 species of hornworts (6 genera, 3 families). The largest family is Lejeuneaceae with 312 species in 30 genera, accounting for 40% of the total number of species. The largest genera are *Cololejeunea*, *Bazzania* and *Frullania* with 90, 61 and 55 species, respectively. The greatest number of species has been recorded from Sabah with 568 species, followed by Pahang and Sarawak with 338 and 265 species, respectively.

## ﻿Introduction

The country of Malaysia consists of two separate regions, Peninsular Malaysia in the West, being part of the Asian mainland, and the states of Sabah and Sarawak on the island of Borneo in the East. Physiogeographically, the two regions are characterized by hills and mountain ranges. On Peninsular Malaysia, mountain ranges cover about one-third of the total land surface, running parallel to the long axis of the peninsula. They include Titiwangsa Range (the main range), Bintang Range, Nakawan Range, Kedah-Singgora Range, Keledang Range, Benom Range, Tahan Range and Pantai Timur Range. Many of them rise to 2000 m and about 23% of the land lies above 300 m (Wyatt-Smith, 1963; Mohamed and Tan 1988). Mount Tahan (2189 m) in Titiwangsa Range, at the border with Thailand, is the highest peak on the peninsula. The rest of the area consists of coastal plains and alluvial terraces, usually extending 15 to 65 km inland from the coast, as well as riverine flood plains.

Sabah’s highlands include Crocker Range, Trus Madi Range, Witti Range, Maitland Range and Brassey Range. Among these, Crocker Range is the longest and highest mountain range, rising to 4095 m on Mt Kinabalu, being the highest mountain of Malaysia and of Southeast Asia. Those of Sarawak are Tama Abu Range, Iran Mountains, Hose Mountains, Kapuas Hulu Range and Kelinkang Range. The physiographies of these two states are somewhat different. Only a small proportion of the land of Sabah consists of lowland, including coastal plains and river valleys. Sarawak, in contrast, has more low-lying, flat lands and swampy coastal areas. Both states, however, have steep undulating hills and mountains in the interior parts, acting as a barrier against inland penetration.

### ﻿Climate

Malaysia is located near the Equator and experiences a humid tropical climate with constant temperatures, high humidity and copious rainfall. Rainfall distribution is usually seasonal dependent on wind flow patterns and physiogeography (Malaysian Meteorological 2021). Generally, the East Coast states of Peninsular Malaysia receive high rainfall almost throughout the year with June and July being the driest months. The remaining states (except for the southwest coastal region) have two maximum rainfall periods (October-November and April-May) that are separated by drier periods (Malaysian Meteorological 2021). The country is subjected to four monsoonal seasons each year: northeast monsoon, southwest monsoon and two shorter inter-monsoon periods. The northeast monsoon normally occurs from November to March and the southwest monsoon during May or early June to September.

### ﻿Vegetation and flora

The vegetation of Malaysia can be classified into different types based on climate and soil. Climatic vegetation types include evergreen and semi-evergreen lowland rainforest, lower montane forest and the upper montane forest, while the edaphic types include heath vegetation, limestone forest, beach vegetation, mangrove forest, brackish water vegetation, peat swamp forest, freshwater swamp forest and riparian vegetation (Symington 2004). In lowland and hill areas, dipterocarp forests are the most prominent forest type (Ashton 1995; Saw 2010). Malaysia has a very rich flora that is made up of Sundaic and mainland Asiatic floristic elements in Peninsular Malaysia and of Sundaic elements with many local endemics on the island of Borneo (Saw and Chung 2015). The Malaysian rainforests stand out by their very high species diversity (e.g., Austin et al. 1973; Baillie and Ashton 1983; Baillie et al. 1987; Saw and Chung 2015), as well as by intricate stratification and regeneration dynamics patterns that have been the subject of numerous major studies by specialists from all over the world (e.g., Burgess 1961, 1975; Denslow 1987; Ashton and Hall 1992; Manokaran et al. 1992; Saiful and Latiff 2017).

### ﻿Malaysian liverwort and hornwort flora

The first checklist of liverworts and hornworts of Malaysia, recognizing 727 species and 37 infraspecific taxa in 127 genera and 39 families, was published by Chuah-Petiot (2011). Lee et al. (2018) presented a review of the history of liverwort exploration in Malaysia and the more recent papers on Malaysian liverworts, and further additions to the liverwort flora were published by Pócs et al. (2020), Pesiu et al. (2021) and Sarimi et al. (2021). A manual of the genera of liverworts and hornworts of Malaysia has been prepared by Lee and Gradstein (2021). The present paper provides an updated checklist of liverworts and hornworts of Malaysia based on a compilation of the published literature and a critical revision of the nomenclature of the taxa and new synonymy, resulting from taxonomic revisions. The updated checklist accepts 773 species and 31 infraspecific taxa in 120 genera in 40 families of liverworts, and 7 species (3 families, 6 genera) of hornworts. The largest family is Lejeuneaceae with 312 species in 30 genera, accounting for 40% of the total number of liverwort species. The largest genera are *Cololejeunea*, *Bazzania* and *Frullania* with 90, 61 and 55 species, respectively. Genera with more than ten species include *Lejeunea* (40), *Plagiochila* (35), *Radula* (34), *Colura* (27), *Drepanolejeunea* (26), *Cheilolejeunea* (24), *Riccardia* (19), *Lepidozia* (18), *Acromastigum* (16), *Leptolejeunea* (16), *Thysananthus* (16), *Solenostoma* (15), *Lopholejeunea* (12), *Heteroscyphus* (13) and *Anastrophyllum* (10). Forty eight genera have fewer than 10 species (e.g., *Acrolejeunea*, *Andrewsianthus*, *Cephalozia*, *Ceratolejeunea*, *Diplasiolejeunea*, *Kurzia*, *Marchantia*, *Microlejeunea*, *Metzgeria*, *Odontoschisma*, *Schistochila*, *Syzygiella*, *Trichocolea* and *Tricholepidozia*) and 54 are represented only by a single species, such as *Acroscyphella*, *Balantiopsis*, *Conoscyphus*, *Eotrichocolea*, *Haplomitrium*, *Jubula*, *Kymatocalyx*, *Mastigopelma*, *Metalejeunea*, *Mesoptygia*, *Nardia*, *Pictolejeunea*, *Soella*, *Southbya*, *Treubia*, *Triandrophyllum*, *Tritomaria* and the five genera of hornworts. Sabah has the largest number of species with 568 recorded species, followed by Pahang and Sarawak with 338, 265, respectively (Table 1).

**Table 1.** The number of recorded species of liverworts and hornworts per state in Malaysia.

State	Number of species		
	Liverworts	Hornworts	Total
Sabah	565	3	568
Pahang	334	4	338
Sarawak	263	2	265
Penang	95	–	95
Perak	82	1	83
Johor	81	–	81
Terengganu	62	–	62
Kedah including Langkawi	59	1	60
Selangor	48	–	48
Negeri Sembilan	44	–	44
Kelantan	35	–	35
Malacca	11	–	11
Perlis	5	–	5

## ﻿An updated checklist of liverworts and hornworts of Malaysia

The nomenclature follows Söderström et al. (2016) with updates. For explanation of some of the new nomenclature, see Lee and Gradstein (2021). In taxon authorities, only the name of the validating author is cited and “ex” authors are omitted for brevity and because their citation is not obligatory. Reports from Malaysia that are only based on secondary information (= papers dealing with liverworts of other regions (not Malaysia) and citing ‘Malaya’, ‘Malaysia’, ‘Malay Peninsular’, ‘Malacca’ etc. in the section on species distribution) and are not supported by direct evidence, are omitted. Similarly, records based on doubtful identifications (“cf.”, “aff.”) are not taken into account. Furthermore, we have omitted all reports citing only “Borneo” unless there is evidence that the record was from Malaysia (Sarawak, Sabah). For example, species reported by Stephani from Borneo based on material collected by Everett (e.g., *Bazzaniafleischeri* (Steph.) Abeyw., *B.inaequitexta* Steph., *Plagiochilaborneensis* Steph., *P.kuhliana* Sande Lac.) are included in the checklist because these Everett collections are presumably from Sarawak (Menzel 1988).

### Liverworts (Marchantiophyta)

***Acrobolbusciliatus*** (Mitt.) Schiffn. – Kodama and Narita (1974). Sabah.

***Acrobolbussaccatus*** (Hook.) Briscoe – Kitagawa and Kodama (1974 as *Tylimanthussaccatus*). Sabah.

***Acrolejeuneaarcuata*** (Nees) Grolle et Gradst. – Kodama and Kitagawa (1974 as *Ptychocoleuscoroniformis*), Gradstein (1975), Kürschner (1990), Lee et al. (2013), Renner (2013), Wang et al. (2014b), Cheah (2017). Pahang, Sabah.

***Acrolejeuneacrassicaulis*** (Steph.) J.Wang bis et Gradst. – Mizutani (1989 as *Trocholejeuneacrassicaulis*), Gradstein et al. (2002 as *T.crassicaulis*), Wang et al. (2014a). Sabah.

***Acrolejeuneafertilis*** (Reinw., Blume et Nees) Schiffn. – Verdoorn (1933 as *Ptychocoleuswichurae*, 1934a as *P.fertilis*), Mizutani (1969 as *P.fertilis*), Gradstein (1975), Lee et al. (2013). Penang, Perak, Pahang, Sabah.

***Acrolejeuneapycnoclada*** (Taylor) Schiffn. – Schiffner (1898), Verdoorn (1933, 1934a both as *Ptychocoleuspycnocladus*), Mizutani (1969 as *P.pycnocladus*), Kitagawa (1971 as *P.pycnocladus*), Kamimura (1974 as *P.pycnocladus*), Gradstein (1975), Lee et al. (2013), Wang et al. (2016). Penang, Perak, Malacca, Sarawak, Sabah.

***Acrolejeuneatjibodensis*** (Verd.) Grolle et Gradst. – Kamimura (1974 as *Ptychocoleustjibodensis*), Gradstein (1975), Lee et al. (2013). Pahang, Sabah.

***Acromastigumaurescens*** A.Evans – Evans (1934). Johor.

***Acromastigumbancanum*** (Sande Lac.) A.Evans – De Notaris (1874 as *Mastigobryumbancanum*), Schiffner (1898 as *Bazzaniabancana*), Evans (1934, also as *A.bidenticulatum*), Herzog (1950), Kitagawa and Kodama (1973), Tixier (1974 as *A.bidenticulatum*), Kürschner (1990), Pócs et al. (2020). Kedah, Johor, Sarawak, Sabah.

***Acromastigumbifidum*** (Steph.) A.Evans – Evans (1934 as *A.denticulatum*), Herzog (1950), Grolle (1978). Sarawak.

***Acromastigumbrotheri*** (Steph.) A.Evans – Stephani (1909 as *Mastigobryumbrotheri*), Evans (1934), Kitagawa and Kodama (1973). Sarawak, Sabah.

***Acromastigumcurtilobum*** A.Evans – Kitagawa and Kodama (1973), Kürschner (1990). Sabah.

***Acromastigumdivaricatum*** (Nees) Reimers – Evans (1934), Kitagawa and Kodama (1973). Johor, Sabah.

***Acromastigumechinatiforme*** (De Not.) A.Evans – De Notaris (1874 as *Mastigobryumechinatiforme*), Schiffner (1898 as *Bazzaniaechinatiformis*), Evans (1934), Herzog (1950), Kitagawa and Kodama (1973), Grolle (1978), Kürschner (1990), Cheah (2017). Pahang, Sarawak, Sabah.

***Acromastigumechinatum*** (Gottsche) A.Evans – Evans (1934), Herzog (1950), Kitagawa and Kodama (1973), Tixier (1974). Kedah, Johor, Sarawak, Sabah.

***Acromastigum ﬁmbriatum*** (Steph.) A.Evans – Stephani (1909 as *Mastigobryumfimbriatum*), Evans (1934), Herzog (1950). Sarawak.

***Acromastigumherzogii*** Grolle – Grolle (1964), Kürschner (1990). Sarawak, Sabah.

***Acromastiguminaequilaterum*** (Lehm. et Lindenb.) A.Evans – De Notaris (1874 as *Mastigobryumelegantulum*), Schiffner (1898 as *Bazzanianotarisii*), Stephani (1909 as *M.notarisii*), Evans (1934), Herzog (1950), Kitagawa (1969c), Kitagawa and Kodama (1973), Kürschner (1990), Wolseley et al. (1996). Penang, Negeri Sembilan, Johor, Sarawak, Sabah.

***Acromastigumlaevigatum*** A.Evans – Evans (1934), Tixier (1974). Kedah, Sarawak.

***Acromastigumleptophyllum*** Herzog – Herzog (1950). Sarawak.

***Acromastigumlinganum*** (De Not.) A.Evans – De Notaris (1874 as *Mastigobryumlinganum*), Schiffner (1898 as *Bazzanialingana*), Stephani (1909 as *M.linganum*), Evans (1934). Sarawak.

***Acromastigumlobuliferum*** A.Evans – Grolle (1978). Sabah.

***Acromastigumobliquatum*** (Mitt.) A.Evans – Kitagawa and Kodama (1973 as *A.gemmiferum*), Grolle (1978). Sabah.

***Acroscyphellatjiwideiensis*** (Sande Lac.) N.Kitag. et Grolle – Cheah (2017). Pahang.

***Allorgellasemperiana*** (Steph.) Bechteler, G.E.Lee, Schäf.-Verw. et Heinrichs – Tixier (1980b as *A.changiana*), Grolle (1985 as *Otolejeuneasemperiana*), Zhu and So (2001 as *O.semperiana*), Schäfer-Verwimp (2006 as *O.semperiana*). Kedah, Perak, Pahang.

***Anastrophyllumassimile*** (Mitt.) Steph. – Kitagawa (1970), Gradstein and Váňa (1987), Váňa and Piippo (1989), Váňa (1991c). Sabah.

***Anastrophyllumauritum*** (Lehm.) Steph. – Kitagawa (1970 as *A.appendiculatum*), Váňa and Piippo (1989), Váňa (1991c). Sabah.

***Anastrophyllumbidens*** (Reinw., Blume et Nees) Steph. – Inoue (1967a, 1968b), Kitagawa (1970), Kitagawa and Kodama (1973), Kürschner (1990), Váňa (1991c). Pahang, Sarawak, Sabah.

***Anastrophyllumbidens*** var. ***aristatum*** N.Kitag. – Herzog (1950), Kitagawa (1970), Kitagawa and Kodama (1973), Kürschner (1990), Váňa (1991c). Sarawak, Sabah.

***Anastrophyllumborneense*** Herzog – Herzog (1950), Inoue (1967a,b, 1968b all as *A.malayense*), Kitagawa (1970), Váňa (1991c). Pahang, Sarawak, Sabah.

***Anastrophyllumfissum*** Steph. – Herzog (1950 as *A.divergens*), Váňa (1991c as *A.divergens*). Sarawak, Sabah.

***Anastrophyllumobtusum*** Herzog – Herzog (1950), Váňa (1991c). Sabah.

***Anastrophyllumpiligerum*** (Nees) Steph. – De Notaris (1874 as *Jungermanniapiligera* [‘*pilifera*’]), Schiffner (1898, also as *A.imbricatum*), Herzog (1950, also as *A.imbricatum*), Inoue (1967a), Kitagawa (1969c, 1970), Kitagawa and Kodama (1973), Kürschner (1990), Váňa (1991c), Álvaro-A (2011). Penang, Pahang, Johor (“Mt. Ophir” (Gunung Ledang) was mistakenly listed as in Malacca), Sarawak, Sabah.

***Anastrophyllumprionophyllum*** S.Hatt. – Kitagawa (1970), Váňa (1991c). Sabah.

***Anastrophyllumrevolutum*** Steph. – Kitagawa (1970), Váňa (1991c), Cheah (2017). Pahang, Sabah.

***Anastrophyllumsquarrosum*** Herzog – Kitagawa (1970), Váňa (1991c), Cheah (2017). Pahang, Sabah.

***Andrewsianthusbidens*** (Steph.) R.M.Schust. – Cheah (2017). Pahang.

***Andrewsianthuschimbuensis*** R.M.Schust. – Kürschner (1990), Váňa (1991c). Sabah.

***Andrewsianthuskinabaluensis*** N.Kitag. – Kitagawa (1970), Váňa (1974, 1991c). Sabah.

***Andrewsianthusmizutanii*** N.Kitag. – Amakawa (1969a as *Jamesoniellaminutissima*), Kitagawa (1969d), Váňa (1974, 1991c). Sabah.

***Andrewsianthuspapillosus*** N.Kitag. – Kitagawa (1970), Kitagawa and Kodama (1973), Váňa (1974, 1991c). Sabah.

***Andrewsianthuspuniceus*** (Nees) Grolle – Kitagawa (1970), Pocock et al. (1984), Váňa (1991c). Pahang, Sabah.

***Andrewsianthusrecurvifolius*** (Nees) R.M.Schust. – Váňa (1991c). Sabah.

***Aneuramaxima*** (Schiffn.) Steph. – Kitagawa and Kodama (1974). Sabah.

***Aneurapinguis*** (L.) Dumort. – Johnson (1972 as *Riccardiapinguis*). Pahang.

***Aphanotropissaxicola*** Herzog – Herzog (1952a). Sarawak. (synonym of *Colura*?, see Gradstein and Benitez 2014, Lee and Gradstein 2021).

***Asterellalimbata*** D.G.Long et Grolle – Long and Grolle (1994), Long (2006). Sabah.

***Balantiopsisciliaris*** S.Hatt. – Hattori (1966), Kitagawa and Kodama (1973). Sabah.

***Bazzaniaalbifolia*** Horik. – Cheah and Yong (2016). Pahang.

***Bazzaniaangustistipula*** N.Kitag. – Cheah and Yong (2016). Pahang.

***Bazzaniaasymmetrica*** (Steph.) N.Kitag. – Cheah and Yong (2016). Pahang.

***Bazzaniabicrenata*** N.Kitag. – Cheah and Yong (2016). Pahang.

***Bazzaniabidentula*** (Steph.) Yasuda – Cheah and Yong (2016). Pahang.

***Bazzaniabilobata*** N.Kitag. – Pócs et al. (2014). Pahang.

***Bazzaniaborneensis*** N.Kitag. – Stephani (1908 as as *Mastigobryumborneense*), Kitagawa (1973). Sarawak.

***Bazzaniacalcarata*** (Sande Lac.) Schiffn. – Stephani (1908 as *Mastigobryumcalcaratum*), Herzog (1950 as *B.richardsii*), Meijer (1960), Tixier (1971). Pahang, Sarawak.

***Bazzaniacaudata*** (Steph.) Herzog – Stephani (1908 as *Mastigobryumcaudatum*), Herzog (1950), Tixier (1971), Kitagawa and Kodama (1973). Penang, Pahang, Sarawak, Sabah.

***Bazzaniaceylanica*** (Mitt.) Steph. – Kamimura (1975). Sabah.

***Bazzaniacincinnata*** (De Not.) Trevis. – De Notaris (1874 as *Mastigobryumcincinnatum*), Schiffner (1898), Stephani (1908 as *M.cincinnatum*), Kamimura (1975). Sarawak, Sabah.

***Bazzaniacommutata*** (Lindenb. et Gottsche) Schiffn. – Tixier (1971). Pahang.

***Bazzaniaconfertifolia*** (Steph.) Herzog – Herzog (1950). Sarawak.

***Bazzaniaconophylla*** (Sande Lac.) Schiffn. – Kitagawa and Kodama (1973), Kitagawa (1973). Sabah.

***Bazzaniadensa*** (Sande Lac.) Schiffn. – Kitagawa (1971), Kitagawa and Kodama (1973), Kürschner (1990). Penang, Sabah.

***Bazzaniadiminuta*** Herzog – Herzog (1950). Sarawak.

***Bazzaniadistans*** (Nees) Trevis. – Johnson (1972). Pahang.

***Bazzaniadrepanophylla*** Herzog – Herzog (1950). Sarawak.

***Bazzaniadulitensis*** Herzog – Herzog (1950), Kitagawa and Kodama (1973), Kürschner (1990). Sarawak, Sabah.

***Bazzaniaerosa*** (Reinw., Blume et Nees) Trevis. – De Notaris (1874 as *Mastigobryumvagum*), Schiffner (1898), Stephani (1908 as *M.vagum*), Herzog (1950 as *B.vaga* and *B.serrulata*), Kitagawa and Kodama (1973), Kitagawa (1977), Cheah and Yong (2016). Penang, Pahang, Sarawak, Sabah.

***Bazzaniaerosa*** var. ***pulopenangensis*** (Lindenb. et Gottsche) Schiffn. – Lindenberg and Gottsche (1851 as *Mastigobryumerosum* δ *pulopenangense*), Schiffner (1898). Penang.

***Bazzaniafallax*** (Sande Lac.) Schiffn. – De Notaris (1874 as *Mastigobryumborneense*), Schiffner (1898), Herzog (1950 as B.fallaxf.fissa), Meijer (1960). Sarawak.

***Bazzania ﬂeischeri*** (Steph.) Abeyw. – Stephani (1908, 1909 both as *Mastigobryumfleischeri*). Sarawak.

***Bazzaniafriabilis*** N.Kitag. et T.Kodama – Kitagawa and Kodama (1973, 1975), Kürschner (1990), Cheah and Yong (2016). Pahang, Sabah.

***Bazzaniagrandiretis*** (Steph.) Herzog – Herzog (1950), Kitagawa and Kodama (1973). Sarawak, Sabah.

***Bazzaniaharpago*** (De Not.) Schiffn. – De Notaris (1874 as *Mastigobryumferox* and *M.harpago*), Schiffner (1898), Stephani (1908 as *M.harpago*), Herzog (1950, 1952b), Kitagawa (1972), Kitagawa and Kodama (1973), Kamimura (1975, also as *B.ferox*), Kürschner (1990), Ludwiczuk and Asakawa (2010), Ng et al. (2021). Sarawak, Sabah.

***Bazzaniaherzogiana*** Meijer – Herzog (1950 as *B.remotifolia*). Sarawak.

***Bazzaniahorridula*** Schiffn. – Herzog (1932 as *Mastigobryummuricatulum*, 1950), Kitagawa and Kodama (1973), Kürschner (1990), Cheah and Yong (2016). Pahang, Sarawak, Sabah.

***Bazzaniainaequitexta*** Steph. – Stephani (1908, 1909 both as *Mastigobryuminaequitextum*). Sarawak.

***Bazzaniaindica*** (Gottsche et Lindenb.) Trevis. – Kamimura (1975), Kürschner (1990). Sabah.

***Bazzaniainsignis*** (De Not.) Trevis. – De Notaris (1874 as *Mastigobryuminsigne*), Schiffner (1898), Evans (1932), Kamimura (1975). Sarawak, Sabah.

***Bazzaniaintermedia*** (Gottsche et Lindenb.) Trevis. – De Notaris (1874 as *Mastigobryumconcinnum*), Schiffner (1898 as *B.concinna*), Stephani (1908 as *M.concinnum*), Herzog (1950 as *B.concinna*), Meijer (1960), Kitagawa (1969c), Tixier (1971), Kitagawa and Kodama (1973), Kamimura (1975), Grolle (1987). Penang, Pahang, Sarawak, Sabah.

***Bazzaniaintermedia*** var. ***sarawakiana*** (De Not.) Schiffn. – De Notaris (1874 as Mastigobryumintermediumvar.sarawakianum), Schiffner (1898). Sarawak.

***Bazzaniainvolutiformis*** (De Not.) Trevis. – De Notaris (1874 as *Mastigobryumduplex* and *M.involutiforme*), Schiffner (1898), Herzog (1950, 1952b), Kitagawa (1972), Kitagawa and Kodama (1973), Kamimura (1975), Kürschner (1990). Sarawak, Sabah.

***Bazzaniakokawana*** N.Kitag. et T.Kodama – Kitagawa and Kodama (1973). Sabah.

***Bazzanialinearis*** Herzog – Herzog (1950). Sarawak.

***Bazzanialinguiformis*** (Sande Lac.) Trevis. – Stephani (1908 as *Mastigobryumlinguiforme* [‘*linguaeforme*’]). Penang.

***Bazzanialongicaulis*** (Sande Lac.) Schiffn. – Herzog (1950), Johnson (1972), Kitagawa and Kodama (1973), Kitagawa (1977), Kürschner (1990). Pahang, Sarawak, Sabah.

***Bazzanialoricata*** (Reinw., Blume et Nees) Trevis. – Stephani (1908 as *Mastigobryumloricatum*), Herzog (1950), Inoue (1967a, 1968b), Pocock et al. (1984). Pahang, Sarawak.

***Bazzanialowii*** (Steph.) Schiffn. – Stephani (1886, 1908 both as *Mastigobryumlowii*), Schiffner (1898), Evans (1932 as *M.lowii*). Sabah.

***Bazzaniamalaccensis*** (Steph.) Tixier – Stephani (1908 as *Mastigobryummalaccense*), Tixier (1971, 1974). Kedah, Perak, Pahang.

***Bazzaniamanillana*** (Steph.) Steph. – Stephani (1917 as *Mastigobryumcrenatistipulum*), Kamimura (1975), Mizutani (1967a). Penang, Sabah.

***Bazzaniamarginella*** (Herzog) N.Kitag. et T.Kodama – Kitagawa and Kodama (1973). Sabah.

***Bazzaniamenzelii*** E.D.Cooper – Herzog (1950 as *Acromastigumemarginatum*). Sarawak.

***Bazzaniaparadoxa*** (Sande Lac.) Steph. – Stephani (1908 as *Mastigobryumnatunense*), Meijer (1960), Kitagawa and Kodama (1973). Pahang, Sarawak, Sabah.

***Bazzaniaparvitexta*** Steph. – Kitagawa (1979a). Sabah.

***Bazzaniapatentistipa*** (Sande Lac.) Schiffn. – Kitagawa and Kodama (1973 [‘*patentistipula*’]). Sabah.

***Bazzaniapectinata*** (Lindenb. et Gottsche) Schiffn. – Herzog (1950, 1952b), Tixier (1971), Kitagawa and Kodama (1973), Pocock et al (1984). Pahang, Sarawak, Sabah.

***Bazzaniapraerupta*** (Reinw., Blume et Nees) Trevis. – Kitagawa and Kodama (1973), Kürschner (1990), Ludwiczuk and Asakawa (2010). Sabah.

***Bazzaniapseudovittata*** N.Kitag. et T.Kodama – Kitagawa and Kodama (1973, 1975), Kürschner (1990), Cheah and Yong (2016). Pahang, Sabah.

***Bazzaniarecurva*** (Mont.) Trevis. – Montagne (1843 as *Herpetiumrecurvum*, 1856 as *Mastigobryumrecurvum*), Gottsche et al. (1845 as *M.recurvum*), Lindenberg and Gottsche (1851 as *M.recurvum*), De Notaris (1874 as M.recurvumvar.pallens), Schiffner (1898 as B.recurvavar.pallens), Stephani (1908 as *M.recurvum*), Inoue (1968b). Penang, Pahang, Sarawak.

***Bazzaniarevoluta*** (Steph.) N.Kitag. – Kamimura (1975 as *B.recurvolimbata*). Sabah.

***Bazzaniaserpentina*** (Nees) Trevis. – Cheah and Yong (2016). Pahang.

***Bazzaniasikkimensis*** Schiffn. – Kamimura (1975). Sabah.

***Bazzaniaspiralis*** (Reinw., Blume et Nees) Meijer – Meijer (1960), Kitagawa and Kodama (1973), Tixier (1971, 1974), Kamimura (1975), Kürschner (1990), Ludwiczuk and Asakawa (2010). Kedah, Pahang, Sarawak, Sabah.

***Bazzaniasubtilis*** (Sande Lac.) Trevis. – De Notaris (1874 as *Mastigobryumsubtile*), Stephani (1917 as *M.repandistipulum*), Meijer (1960), Kitagawa and Kodama (1973), Kitagawa (1977, 1979 as *B.palmatifida*), Kürschner (1990), Wolseley et al. (1996). Negeri Sembilan, Sarawak, Sabah.

***Bazzaniasumbavensis*** (Steph.) Steph. – Kamimura (1975). Sabah.

***Bazzaniatridens*** (Reinw., Blume et Nees) Trevis. – Schiffner (1898 as *B.australis*), Herzog (1950, also as *B.coreana*), Kitagawa (1969c, 1972, also as *B.australis*), Tixier (1971), Kitagawa and Kodama (1973, also as *B.australis*), Kamimura (1975, also as B.tridensf.minutissima), Kürschner (1990). Penang, Pahang, Sarawak, Sabah.

***Bazzaniatridens*** var. ***assamica*** (Steph.) Pócs – Kamimura (1975), Pócs et al. (2014 as *B.assamica*). Pahang, Sabah.

***Bazzaniauncigera*** (Reinw., Blume et Nees) Trevis. – Herzog (1950), Kitagawa and Kodama (1973), Kürschner (1990), Cheah and Yong (2016). Pahang, Sarawak, Sabah.

***Bazzaniauncigera*** var. ***brevifolia*** Herzog – Herzog (1950). Sarawak.

***Bazzaniaundulata*** Herzog – Herzog (1950). Sarawak.

***Bazzaniavittata*** (Gottsche) Trevis. – Herzog (1950), Inoue (1967a), Kitagawa and Kodama (1973), Kürschner (1990). Pahang, Sarawak, Sabah.

***Bazzaniavittata*** var. ***luxurians*** (De Not.) Schiffn. – De Notaris (1874 as Mastigobryumvittatumvar.luxurians), Schiffner (1898). Sarawak.

***Bazzaniawiltensii*** (Steph.) Schiffn. – Pocock et al. (1984). Pahang.

***Bazzaniazollingeri*** (Lindenb.) Trevis. – De Notaris (1874 as *Mastigobryumzollingeri*). Sarawak.

***Blepharostomatrichophyllum*** (L.) Dumort. – Akiyama et al. (2001). Sabah.

***Calypogeiaapiculata*** (Steph.) Steph. – Kitagawa and Kodama (1973). Sabah.

***Calypogeiaarguta*** Nees et Mont – Wolseley et al. (1996), Bisang et al. (2003). Pahang, Negeri Sembilan.

***Caudalejeuneacristiloba*** (Steph.) Gradst. – Verdoorn (1934a, 1937 both as *C.circinata*), Mizutani (1966, 1969 both as *C.circinata*, 1988), Kitagawa and Kodama (1974 as *C.circinata*). Johor, Sarawak, Sabah.

***Caudalejeuneareniloba*** (Gottsche) Steph. – Stephani (1912 as *C.serrata*), Verdoorn (1934a), Herzog (1952b), Mizutani (1966, 1969, also as *C.stephanii*, 1988, also as *C.recurvistipula*), Tixier (1971 as *C.stephanii*, 1980a), Kitagawa and Kodama (1974), Thiers (1985), Pócs et al. (2020), Pesiu et al. (2021, in press). Penang, Perak, Selangor, Pahang, Malacca, Terengganu, Johor, Sarawak, Sabah.

***Cephalolejeuneaparvilobula*** Mizut. – Mizutani (1979b). Sabah.

***Cephaloziaacuminata*** (Herzog) Váňa – Herzog (1950 as *Metahygrobiellaacuminata*), Váňa (1993 as *M.acuminata*). Sarawak.

***Cephaloziaacutiloba*** (Inoue) Váňa – Inoue (1967a,b, both as *Metahygrobiellaacutiloba*). Pahang.

***Cephaloziahamatiloba*** Steph. – Müller and Schäfer-Verwimp (1999), Cheah (2017). Pahang.

***Cephaloziamollusca*** (De Not.) Váňa – De Notaris (1874 as *Jungermanniamollusca*), Schiffner (1898 as *Hygrobiellamollusca*), Stephani (1908 as *H.mollusca*), Herzog (1950 as *Metahygrobiellamollusca*), Kitagawa and Kodama (1974 as *M.mollusca*), Váňa (1993 as *M.mollusca*). Sarawak, Sabah.

***Cephaloziastolonacea*** (Herzog) Váňa – Herzog (1950 as *Metahygrobiellastolonacea* and Hygrobiellamolluscaf.subintegerrima), Grolle (1963 as *M.stolonacea*), Váňa (1993 as *M.stolonacea*). Sarawak.

***Cephaloziellacapillaris*** (Steph.) Douin – Inoue (1967a), Inoue and Miller (1967). Pahang.

***Cephaloziellakiaeri*** (Austin) Douin – Kitagawa and Kodama (1974 as *C.willisana*), Váňa (1992). Sabah.

***Cephaloziellamicrophylla*** (Steph.) Douin – Kitagawa (1969c). Penang.

***Ceratolejeuneaaliena*** Herzog – Herzog (1952b), Kitagawa and Kodama (1974), Mizutani (1981a). Sarawak, Sabah.

***Ceratolejeuneabelangeriana*** (Gottsche) Steph. – Kitagawa and Kodama (1974, also as *C.ryukyuensis*), Mizutani (1981a as *C.oceanica*), Zhu et al. (2005), Cheah (2017). Pahang, Johor, Sabah.

***Ceratolejeuneacornuta*** (Lindenb.) Steph. – Inoue (1967a as *C.tahitensis*). Pahang.

***Ceratolejeuneaminor*** Mizut. – Mizutani (1981a), Zhu et al. (2005), Sarimi et al. (2021). Terengganu, Sabah.

***Ceratolejeuneamoniliata*** Herzog – Herzog (1952b), Mizutani (1970, 1981a), Pesiu et al. (in press). Terengganu, Johor, Sarawak, Sabah.

***Ceratolejeuneasingapurensis*** (Lindenb.) Steph. – Herzog (1952b), Cheah (2017), Sarimi et al. (2021). Pahang, Terengganu, Sarawak.

***Ceratolejeuneaspinistipula*** (Herzog) R.L.Zhu, L.Shu, Xiong He et Y.M.Wei – Herzog (1948 as *Drepanolejeuneaspinistipula*), Pócs et al. (1995 as *Acantholejeuneaspinistipula*), Zhu et al. (2018a as *D.spinistipula*). Johor, Sarawak, Sabah.

***Cheilolejeuneaadnata*** (Lehm.) Grolle var. ***autoica*** Gradst. et Ilk.-Borg. – Shu et al. (2015 as *C.larsenii*). Penang.

***Cheilolejeuneaceylanica*** (Gottsche) R.M.Schust. et Kachroo – Schiffner (1898 as *Pycnolejeuneaceylanica*), Mizutani (1966, 1970, 1980), Tixier (1972, 1974 both as *P.arietina*, 1980a as *Xenolejeuneaceylanica*), Kitagawa and Kodama (1974), Grolle (1979), Kürschner (1990), Wolseley et al. (1996), Zhu and So (2001), Pócs et al. (2020), Pesiu et al. (2021). Kedah, Penang, Pahang, Negeri Sembilan, Malacca, Terengganu, Sabah.

***Cheilolejeuneadecursiva*** (Sande Lac.) R.M.Schust. – Mizutani (1970 as *C.spathulata*), Grolle (1977), Zhu and Lai (2005), Shu et al. (2014), Cheah (2017). Pahang, Sabah.

***Cheilolejeuneafalsinervis*** (Sande Lac.) R.M.Schust. et Kachroo – Mizutani (1966, 1970), Kitagawa and Kodama (1974), Tixier (1974 as *Xenolejeuneafalsinervis*), Thiers (1986), Kürschner (1990), Wolseley et al. (1996). Kedah, Negeri Sembilan, Sabah.

***Cheilolejeuneagigantea*** (Steph.) R.M.Schust. et Kachroo – Herzog (1950 as *Pycnolejeuneagigantea*). Sarawak.

***Cheilolejeuneaincisa*** (Gottsche) R.M.Schust. et Kachroo – Hoffmann (1935 as *Pycnolejeuneaexcisula*), Mizutani (1970 as *C.excisula*), Tixier (1971 as *P.excisula*), Kamimura (1974 as *C.excisula*), Kitagawa and Kodama (1974 as *C.excisula*), Grolle and Piippo (1990 as *C.excisula*). Pahang, Johor, Sabah.

***Cheilolejeuneainsignis*** Jovet-Ast et Tixier – Kitagawa and Kodama (1974). Sabah.

***Cheilolejeuneaintertexta*** (Lindenb.) Steph. – Mizutani (1967b, 1970), Kitagawa (1971), Grolle (1979), Kürschner (1990), Pócs et al. (2020). Penang, Sarawak, Sabah.

***Cheilolejeuneakrakakammae*** (Lindenb.) R.M.Schust. – Cheah (2017). Pahang.

***Cheilolejeunealindenbergii*** (Gottsche) Mizut. – Herzog (1950 as *Hygrolejeunealindenbergii* and *Euosmolejeunealuerssenii*), Inoue (1967 as *C.luerssenii* [‘*luersonii*’]), Kitagawa (1969a as *C.luerssenii*, 1971), Mizutani (1970), Kitagawa and Kodama (1974), Kürschner (1990), Cheah (2017). Penang, Pahang, Sarawak, Sabah.

***Cheilolejeunealoriana*** (Steph.) W.Ye et R.L.Zhu – Mizutani (1970 as *Leucolejeunealoriana*). Sabah.

***Cheilolejeuneamalaccensis*** (G.Hoffm.) Xiao L.He – Hoffmann (1935 as *Pycnolejeuneamalaccensis*), Thiers (1986 as *P.malaccensis*), He (1996). Penang.

***Cheilolejeuneamicholitzii*** (Steph.) R.M.Schust. et Kachroo – Hoffmann (1935 as *Pycnolejeuneamicholitzii*). Johor.

***Cheilolejeuneamizutanii*** W.Ye et R.L.Zhu – Mizutani (1976 as *Leucolejeuneadecurrens*), Grolle and Piippo (1990 as *L.decurrens*). Sabah.

***Cheilolejeuneanipponica*** (S.Hatt.) S.Hatt. – Pócs and Lee (2016). Selangor.

***Cheilolejeuneaocclusa*** (Herzog) T.Kodama et N.Kitag. – Herzog (1950 as *Strepsilejeuneaocclusa*), Kitagawa and Kodama (1974), Mizutani (1980), Wolseley et al. (1996), Pócs et al. (2020). Negeri Sembilan, Sarawak, Sabah.

***Cheilolejeuneaorientalis*** (Gottsche) Mizut. – Kitagawa and Kodama (1974). Sabah.

***Cheilolejeuneaserpentina*** (Mitt.) Mizut. – Hashimoto et al. (1993), Cheah (2017). Langkawi, Pahang.

***Cheilolejeuneastreimannii*** Pócs et Ninh – Pócs and Lee (2016). Pahang.

***Cheilolejeuneatrapezia*** (Nees) R.M.Schust. et Kachroo – Stephani (1890 as *Lejeuneaimbricata*), Hoffmann (1935 as *Pycnolejeuneaimbricata*, P.meyenianaand itsvar.ligulata, *C.longiloba*), Herzog (1948 as *Placolejeuneasubarrhyncha*), Mizutani (1966, 1970 both as *C.imbricata*, 1980 as *C.longiloba*), Inoue (1967a as *C.imbricata*), Kitagawa (1971 as *C.imbricata*), Tixier (1971, 1974, 1980a as *Xenolejeunealongiloba* and *X.meyeniana*), Kitagawa and Kodama (1974 as *C.imbricata* and *C.meyeniana*), Grolle (1979), Thiers (1986 as *C.meyeniana*), Kürschner (1990 as *C.imbricata*), Cheah (2017 as *C.imbricata*), Pócs et al. (2020, also as *C.meyeniana*), Pesiu et al. (2021). Kedah, Penang, Pahang, Terengganu, Johor, Sarawak, Sabah.

***Cheilolejeuneatrifaria*** (Reinw., Blume et Nees) Mizut. – Inoue (1967a as *C.viridula*), Mizutani (1970, 1982), Kamimura (1974), Kitagawa and Kodama (1974), Hashimoto et al. (1993), Lee et al. (2013), Pócs et al. (2020), Pesiu et al. (2021). Langkawi, Perak, Pahang, Terengganu, Sabah.

***Cheilolejeuneaventricosa*** (P.Syd.) Xiao L.He – Hoffmann (1935 as *Pycnolejeuneaventricosa*), Mizutani (1970 as *P.ventricosa*), Hattori and Kamimura (1971 as *P.ventricosa*), Zhu and Lai (2005), Pócs et al. (2020). Penang, Sabah.

***Cheilolejeuneaverrucosa*** Steph. – Tixier (1974), Mizutani (1979b). Kedah, Sabah.

***Cheilolejeuneavittata*** (G.Hoffm.) R.M.Schust. et Kachroo – Mizutani (1970, 1980), Kürschner (1990), Wolseley et al. (1996), Pócs et al. (2020). Negeri Sembilan, Sabah.

***Cheilolejeuneaxanthocarpa*** (Lehm. et Lindenb.) Malombe – Verdoorn (1934a as *Leucolejeuneaxanthocarpa*), Inoue (1967a as *L.xanthocarpa*), Tixier (1971 as *L.xanthocarpa*), Kamimura (1975 as *L.xanthocarpa*). Pahang, Sabah.

***Chiastocaulonbraunianum*** (Nees) S.D.F.Patzak, M.A.M Renner, Schäf.-Verw. et Heinrichs – Kitagawa and Kodama (1974 as *Plagiochilionbraunianum*), Akiyama et al. (2001 as *P.braunianum*). Sabah.

***Chiastocaulondendroides*** (Nees) Carl – De Notaris (1874 as *Plagiochiladendroides*), Schiffner (1898 as *P.dendroides*), Herzog (1950 as *P.dendroides*), Kitagawa and Kodama (1974 as *P.dendroides*), Inoue (1967a, 1968b, 1984 as *P.dendroides*), Kürschner (1990 as *P.dendroides*), So (2001 as *P.dendroides*), Akiyama et al. (2001 as *P.dendroides*). Pahang, Sarawak, Sabah.

***Chiastocaulonoppositum*** (Reinw., Blume et Nees) S.D.F.Patzak, M.A.M Renner, Schäf.-Verw. et Heinrichs – Stephani (1904 as *Plagiochilaopposita*), Hattori (1942a as *Plagiochilaopposita*, 1950), Inoue (1964, 1967a both as *Plagiochilionoppositum*), Kitagawa and Kodama (1974 as *Plagiochilionoppositum*), Kamimura (1975 as *Plagiochilionoppositum*), Kürschner (1990 as *Plagiochilionoppositum*). Perak, Pahang, Sarawak, Sabah.

***Chiastocaulonpachycephalum*** (De Not.) Herzog – De Notaris (1874 as *Plagiochilapachycephala*), Schiffner (1898 as *Plagiochilapachycephala*), Herzog (1950 as *Plagiochilionpachycephalum*), Inoue (1964 as *Plagiochilionpachycephalum*), So and Grolle (2000 as *Plagiochilionpachycephalum*), So (2001 as *Plagiochilapachycephala*). Sarawak.

***Chiastocaulontheriotianum*** (Steph.) S.D.F.Patzak, M.A.M Renner, Schäf.-Verw. et Heinrichs – Inoue (1964 as *Plagiochiliontheriotianum*). Sarawak.

***Cololejeuneaabnormis*** Mizut. – Mizutani (1970), Tixier (1985). Penang, Sabah.

***Cololejeuneaacuminata*** Mizut. – Mizutani (1970), Wolseley et al. (1996), Pócs et al. (2020). Negeri Sembilan, Sabah.

***Cololejeuneaaequabilis*** (Sande Lac.) Schiffn. – Benedix (1953 as *C.yulensis*), Mizutani (1966 as *C.yulensis*), Kitagawa and Kodama (1974 as *C.yulensis*), Pesiu et al. (2021). Terengganu, Johor, Sarawak, Sabah.

***Cololejeuneaamphibola*** B.M.Thiers – Eggers et al. (1998), Pócs and Lee (2016). Selangor, Kelantan, Pahang, Sarawak.

***Cololejeuneaangulata*** (Steph.) Mizut. – Kitagawa (1969a, 1969c), Wolseley et al. (1996), Mizutani (1966). Penang, Negeri Sembilan, Sabah.

***Cololejeuneaangustiﬂora*** (Steph.) Mizut. – Mizutani (1966, 1970 both as *C.javanica* and *C.crenulata*), Kitagawa and Kodama (1974 as *C.crenulata*), Tixier (1985 as *C.javanica*), Zhu and So (2000a as *C.mackeeana*, 2002). Penang, Sarawak, Sabah.

***Cololejeuneaappressa*** (A.Evans) Benedix – Mizutani (1966, 1970), Sarimi et al. (2021). Terengganu, Sabah.

***Cololejeuneabidentula*** (Steph.) E.W.Jones – Pócs and Lee (2016). Selangor.

***Cololejeuneaceatocarpa*** (Ångstr.) Steph – Pócs and Lee (2016). Kelantan, Pahang.

***Cololejeuneaceratilobula*** (P.C.Chen) R.M.Schust. – Mizutani (1966, 1970 both as *C.formosana*), Pesiu et al. (in press). Terengganu, Sabah.

***Cololejeuneachamlongiana*** Tixier – Tixier (1985). Perak.

***Cololejeuneachinii*** Tixier – Tixier (1973b), Kitagawa (1981). Selangor, Kelantan.

***Cololejeuneacordiflora*** Steph. – Tixier (1971 as *C.karstenii*, 1985). Perak, Penang, Pahang.

***Cololejeuneadecliviloba*** Steph. – Tixier (1980a). Pahang.

***Cololejeuneadesciscens*** Steph. – Eggers et al. (1998). Sarawak.

***Cololejeuneadiaphana*** A.Evans – Eggers et al. (1998 as *Aphanolejeuneaproboscoidea*), Pócs and Lee (2016). Kelantan, Sarawak.

***Cololejeuneadilatata*** (Steph.) Mizut. – Mizutani (1966, 1970). Sabah.

***Cololejeuneadozyana*** (Sande Lac.) Schiffn. – Benedix (1953), Tixier (1980a), Pócs et al. (2020). Pahang, Sabah.

***Cololejeuneaensifera*** Tixier – Pócs et al. (2020), Sarimi et al. (2021). Sabah.

***Cololejeuneaequialbi*** Tixier – Pócs et al. (2020), Sarimi et al. (2021), Pesiu et al (in press). Terengganu, Sabah.

***Cololejeuneafalcata*** (Horik.) Benedix – Mizutani (1966, 1970 both as *C.falcatoides*), Tixier (1971, 1974 both as *C.falcatoides*, 1978, 1980a), Kitagawa and Kodama (1974 as *C.falcatoides*), Pesiu et al. (2021, in press). Kedah, Perak, Penang, Pahang, Terengganu, Sabah.

***Cololejeuneaﬁlidens*** Benedix – Tixier (1985). Perak, Selangor.

***Cololejeuneafissilobula*** Herzog – Herzog (1950). Sarawak.

***Cololejeuneaﬂoccosa*** (Lehm. et Lindenb.) Schiffn. – Benedix (1953), Kitagawa (1969c), Mizutani (1966, 1970, 1984a), Tixier (1971, 1974, 1978), Kitagawa and Kodama (1974), Wolseley et al. (1996), Pesiu et al. (2021, in press). Kedah, Penang, Perak, Pahang, Negeri Sembilan, Terengganu, Sarawak, Sabah.

***Cololejeuneafloccosa*** var. ***amoena*** (Benedix) Pócs – Benedix (1953 as *C.amoena*), Mizutani (1966, 1970, both as *C.amoena*). Johor, Sabah.

***Cololejeuneafloccosa*** var. ***aurita*** Benedix – Benedix (1953), Tixier (1978). Kedah, Penang, Perak, Johor.

***Cololejeuneafloccosa*** var. ***trivittata*** Tixier – Eggers et al. (1998 as C.floccosavar.vittata), Crosby and Engel (2006). Pahang, Sarawak.

***Cololejeuneafructumarginata*** Tixier – Tixier (1985). Penang.

***Cololejeuneageorgiana*** Tixier – Tixier (1985). Penang.

***Cololejeuneagottschei*** (Steph.) Pandé, K.P.Srivast. et Ahmad – Mizutani (1966 as *C.paroica*), Pócs et al. (2020), Sarimi et al. (2021), Pesiu et al. (in press). Kelantan, Terengganu, Sabah.

***Cololejeuneagradsteinii*** M.J.Lai et R.L.Zhu – Tixier (1971 as *C.pusilla*). Pahang.

***Cololejeuneagynophthalma*** Benedix – Tixier (1971, 1978), Mizutani (1966, 1970). Penang, Pahang, Sabah.

***Cololejeuneahaskarliana*** (Lehm.) Schiffn. – Benedix (1953), Mizutani (1966, 1970, 1986a), Kitagawa (1971), Kitagawa and Kodama (1974), Tixier (1980a, 1985), Wolseley et al. (1996), Pócs et al. (2020). Penang, Perak, Pahang, Negeri Sembilan, Johor, Sarawak, Sabah.

***Cololejeuneahattoriana*** Mizut. et Pócs – Mizutani (1966 as *Campylolejeuneapusilla*). Sabah.

***Cololejeuneahildebrandii*** (Austin) Steph. – Tixier (1980a as *C.filicaulis*, 1985), Pócs et al. (2014, 2020). Penang, Perak, Selangor, Pahang, Sabah.

***Cololejeuneahirta*** Steph. – Tixier (1985). Perak, Pahang.

***Cololejeuneaindica*** Pandé et R.N.Misra – Tixier (1985 as *C.planiflora*). Penang.

***Cololejeuneainﬂata*** Steph. – Benedix (1953 as *C.oshimensis*), Mizutani (1966, 1970 both as *C.oshimensis*), Tixier (1971, 1974 both as *C.oshimensis*, 1978, 1980a), Kitagawa and Kodama (1974 as *C.oshimensis*), Pesiu et al. (2021). Kedah, Pahang, Terengganu, Johor, Sabah.

***Cololejeuneainflectens*** (Mitt.) Benedix – Benedix (1953 as *C.ciliatilobula* and *C.peculiaris*), Mizutani (1970 as *Campylolejeuneapeculiaris*), Tixier (1971 as *C.ciliatilobula*, 1974 as *Campylolejeuneapeculiaris*, 1980a as *C.inflectidens*), Pesiu et al. (2021). Kedah, Pahang, Terengganu, Johor, Sarawak, Sabah.

***Cololejeuneajohannis-winkleri*** (Herzog) R.L.Zhu – Zhu et al. (2004). Sabah.

***Cololejeuneakulenensis*** Tixier – Tixier (1985). Johor.

***Cololejeunealacinulata*** Benedix – Benedix (1953). Sabah.

***Cololejeunealanciloba*** Steph. – Mizutani (1966, 1984b), Kitagawa (1971), Tixier (1971 as *C.pahangiana*, 1985 as *C.bolombensis*), Kitagawa and Kodama (1974), Pócs et al. (2020), Pesiu et al. (2021, in press). Penang, Pahang, Terengganu, Johor, Sabah.

***Cololejeunealatilobula*** (Herzog) Tixier – Pócs and Lee (2016). Kelantan.

***Cololejeunealongifolia*** (Mitt.) Mizut. – Tixier (1984, 1985), Pócs et al. (2020). Penang, Johor, Sabah.

***Cololejeuneamacounii*** (Spruce) A.Evans – Mizutani (1966, 1970 both as *C.kinabalensis*), Pócs et al. (2020). Sabah.

***Cololejeuneamadothecoides*** (Steph.) Benedix. – Benedix (1953). Sarawak.

***Cololejeuneamagnilobula*** (Horik.) S.Hatt. – Pócs et al. (2020). Sabah.

***Cololejeuneamagnilobula*** var. ***falcidentata*** Pócs et G.E.Lee – Pócs and Lee (2016). Kelantan.

***Cololejeuneamalaccensis*** Tixier – Tixier (1985). Johor.

***Cololejeuneamalayana*** Tixier – Tixier (1980a, 1985), Eggers (2006). Perak, Pahang.

***Cololejeuneamaritima*** Tixier – Tixier (1979, 1985). Perak, Johor.

***Cololejeuneametzgeriopsis*** (K.I.Goebel) Gradst., R.Wilson, Ilk.-Borg. et Heinrichs – Mizutani (1966 as *Metzgeriopsispusilla*), Kitagawa (1969b as *M.pusilla*), Kitagawa and Kodama (1974 as *M.pusilla*), Tixier (1980a as *M.pusilla*), Akiyama et al. (2001 as *M.pusilla*), Gradstein et al. (2006), Pócs and Piippo (2011), Pócs et al. (2020), Pesiu et al. (2021). Perak, Pahang, Terengganu, Sabah.

***Cololejeuneamutabilis*** Benedix – Benedix (1953 as C.mutabilisf.borneensis), Tixier (1978 as C.mutabilisf.borneensis,f.floccodoidesandf.subfalcata), Mizutani (1966), Pócs et al. (2020). Perak, Sabah.

***Cololejeuneaobliqua*** (Nees et Mont.) Schiffn. – Benedix (1953 as *C.androgyna*, *C.nymannii*), Mizutani (1966 as *C.jelinekii* and *C.nymannii*, 1970 as *C.nymannii*), Tixier (1971 as *C.jelinekii*, 1980a, 1985 both as *C.scabriflora*), Kitagawa and Kodama (1974 as *C.nymannii*), Asthana and Srivastava (2003 as *C.jelinekii*), Pócs et al. (2020), Pesiu et al. (2021). Penang, Perak, Pahang, Terengganu, Sarawak, Sabah.

***Cololejeuneaocellata*** (Horik.) Benedix – Pócs and Lee (2016). Pahang.

***Cololejeuneaocelloides*** (Horik.) Mizut. – Benedix (1953 as C.leonidensand itsvar.saccata), Mizutani (1966, 1970 both as *C.leonidens*, 1984a), Inoue (1967a as *C.leonidens*), Tixier (1969 as *C.meijeri*, 1971, 1978, 1980a all as *C.leonidens*), Kitagawa and Kodama (1974 as *C.leonidens*), Pesiu et al. (2021). Perak, Pahang, Terengganu, Sabah.

***Cololejeuneapacifica*** Pócs – Pócs and Lee (2016). Kelantan.

***Cololejeuneapapillosa*** (K.I.Goebel) Mizut. – Herzog (1950 as Aphanolejeuneamicroscopicavar.borneensis), Mizutani (1966), Pócs and Piippo (1999 as *A.borneensis*), Pócs and Bernecker (2009), Pócs and Lee (2016), Pócs et al. (2020). Pahang, Sarawak, Sabah.

***Cololejeuneapeponiformis*** Mizut. – Mizutani (1970), Pócs et al. (2020). Sabah.

***Cololejeuneaperaffinis*** (Schiffn.) Schiffn. – Herzog (1952b as *Taeniolejeuneaperaffinis*), Benedix (1953), Mizutani (1966, 1970, 1984a), Kitagawa (1969a), Kitagawa and Kodama (1974), Tixier (1978 as *C.peraffinis*var.elegans, 1980a). Penang, Pahang, Johor, Sarawak, Sabah.

***Cololejeuneaperakensis*** Tixier – Tixier (1985). Perak.

***Cololejeuneaplanissima*** (Mitt.) Abeyw. – Mizutani (1970, 1984b), Kitagawa (1971), Kitagawa and Kodama (1974), Tixier (1985), Asthana and Srivastava (2003), Pócs et al. (2020), Pesiu et al. (2021). Penang, Perak, Terengganu, Johor, Sabah.

***Cololejeuneaplatyneura*** (Spruce) A.Evans – Mizutani (1966 as *C.astyla*), Zhu and So (1998b, 2001), Cheah (2017). Pahang, Sabah

***Cololejeuneapretiosa*** Benedix – Kitagawa and Kodama (1974). Sabah.

***Cololejeunea pseudoﬂoccosa*** (Horik.) Benedix – Benedix (1953), Mizutani (1966, 1970), Kitagawa and Kodama (1974), Tixier (1980a). Pahang, Sabah.

***Cololejeuneapseudoschmidtii*** Tixier – Tixier (1985). Penang.

***Cololejeuneapseudostephanii*** Tixier – Pócs and Lee (2016). Pahang.

***Cololejeuneapseudostipulata*** P.Syd. – Mizutani (1970). Sabah.

***Cololejeunearaduliloba*** Steph. – Mizutani (1966), Pócs et al. (2020). Sabah.

***Cololejeuneareineckeana*** Steph. – Tixier (1985). Perak.

***Cololejeunearosellata*** Mizut. – Mizutani (1966), Kitagawa and Kodama (1974), Tixier (1985). Sabah.

***Cololejeuneaschmidtii*** Steph. – Mizutani (1966, 1970 as *C.benedixii*), Kitagawa and Kodama (1974), Cheah (2017), Sarimi et al. (2021), Pesiu et al. (in press). Pahang, Terengganu, Sabah.

***Cololejeuneaselangorensis*** Tixier – Tixier (1985). Selangor.

***Cololejeuneaserrulata*** Steph. – Kitagawa (1971). Penang.

***Cololejeuneasetosa*** Mizut. – Mizutani (1966), Eggers et al. (1998). Sarawak, Sabah.

***Cololejeuneashimizui*** N.Kitag. – Kitagawa (1969c, 1981). Kelantan.

***Cololejeuneasiamensis*** Steph. – Benedix (1953 as *C.pluriplicata*, orth. var. of *C.pluripunctata*), Mizutani (1966, 1970 both as *C.pluripunctata*), Tixier (1978), Wolseley et al. (1996). Penang, Negeri Sembilan, Johor, Sabah.

***Cololejeuneasigmoidea*** Jovet-Ast et Tixier – Mizutani (1966), Kitagawa (1969c), Tixier (1971, 1980a), Kitagawa and Kodama (1974), Bernecker-Lücking and Morales (1999), Pesiu et al. (2021). Penang, Pahang, Terengganu, Sabah.

***Cololejeuneasigmoidea*** var. ***dubia*** Tixier – Tixier (1985). Perak.

***Cololejeuneasmitinandii*** Tixier – Pócs and Lee (2016). Kelantan.

***Cololejeuneaspathulifolia*** (Steph.) H.A.Mill. – Pócs and Lee (2016). Kelantan.

***Cololejeuneasphaerodonta*** Mizut. – Mizutani (1966), Zhu (1995), Zhu and So (2001), Pócs et al. (2020). Sabah.

***Cololejeuneastephanii*** Benedix – Benedix (1953), Mizutani (1966, 1970), Kitagawa and Kodama (1974), Tixier (1974, 1978, 1980a), Zhu (1995), Eggers et al. (1998), Pócs et al. (2020), Pesiu et al. (2021, in press). Kedah, Perak, Pahang, Terengganu, Johor, Sarawak, Sabah.

***Cololejeuneastoniana*** Tixier – Tixier (1976, 1985), Eggers et al. (1998). Johor, Sarawak.

***Cololejeuneastylosa*** Steph. – Tixier (1985), Pócs and Lee (2016), Pócs et al. (2020). Penang, Kelantan, Sabah.

***Cololejeuneatenella*** Benedix – Benedix (1953), Tixier (1974, 1978, 1985 the latter two as C.tenellavar.vittata), Wolseley et al. (1996), Pócs et al. (2020). Kedah, Penang, Perak, Negeri Sembilan, Johor, Sabah.

***Cololejeuneathailandensis*** Tixier – Tixier (1985). Penang.

***Cololejeuneatriapiculata*** (Herzog) Tixier – Tixier (1971, 1980a, 1985). Pahang.

***Cololejeuneatrichomanis*** (Gottsche) Besch. – Mizutani (1966, 1970 both as *C.goebelii*), Kitagawa (1971 as *C.goebelii*), Kitagawa and Kodama (1974 as *C.goebelii*), Tixier (1974, 1985 as *C.balansae* and *C.goebelii*), Asthana and Srivastava (2003). Kedah, Penang, Perak, Sabah.

***Cololejeuneatridentata*** Tixier – Tixier (1985), Eggers et al. (1998). Johor, Sarawak.

***Cololejeuneauchimae*** Amakawa – Tixier (1985 as *C.pakseana*). Selangor.

***Cololejeuneaveillonii*** Tixier – Pócs and Lee (2016). Pahang.

***Cololejeuneaverrucosa*** Steph. – Benedix (1953), Pesiu et al. (2021, in press). Terengganu, Johor.

***Cololejeuneaverrucosa*** var. ***rectispina*** (Herzog) Benedix – Benedix (1953), Kitagawa and Kodama (1974). Sarawak, Sabah.

***Cololejeuneawightii*** Steph. – Stephani (1895), Kitagawa (1971, 1972), Tixier (1971, 1985). Penang, Pahang.

***Coluraacroloba*** (Prantl) Jovet-Ast – Jovet-Ast (1954, 1967, 1976), Mizutani (1966, 1970), Kitagawa (1969a), Tixier (1971, 1980a), Kitagawa and Kodama (1974), Wolseley et al. (1996), Pócs et al. (2020), Pesiu et al. (2021, in press). Kedah, Selangor, Pahang, Negeri Sembilan, Terengganu, Johor, Sarawak, Sabah.

***Coluraari*** (Steph.) Steph. – Herzog (1952b as *C.javanica*), Jovet-Ast (1976), Mizutani (1966, 1970), Sarimi et al. (2021), Pesiu et al. (in press). Perak, Terengganu, Sarawak, Sabah.

***Colurabisvoluta*** Herzog et Jovet-Ast – Jovet-Ast (1954). Malacca.

***Colurabrevistyla*** Herzog – Pócs and Lee (2016). Kelantan.

***Coluraclementis*** Grolle – Jovet-Ast (1967). Sabah.

***Coluraconica*** (Sande Lac.) K.I.Goebel – Jovet-Ast (1958, 1967), Mizutani (1966, 1970 both as *C.acutifolia*), Kitagawa (1969a as *C.acutifolia*), Kitagawa and Kodama (1974), Wolseley et al. (1996), Pócs et al. (2020), Pesiu et al. (2021, in press). Penang, Negeri Sembilan, Terengganu, Sarawak, Sabah.

***Coluracorynophora*** (Nees, Lindenb. et Gottsche) Trevis. – Herzog (1952b as *C.trialata*), Mizutani (1966, 1970), Jovet-Ast (1954, 1958, 1967, 1976), Kitagawa (1969c), Wolseley et al. (1996), Pócs et al. (2020), Pesiu et al. (2021, in press). Penang, Perak, Negeri Sembilan, Terengganu, Johor, Sarawak, Sabah.

***Coluracrenulata*** Grolle – Jovet-Ast (1967). Sabah.

***Coluracristata*** Jovet-Ast – Jovet-Ast (1976), Sangrattanaprasert et al. (2019), Pócs et al. (2020). Penang, Sabah.

***Coluracymbalifera*** Herzog et Jovet-Ast – Jovet-Ast (1954). Peninsular Malaysia (type locality).

***Coluragaleata*** Jovet-Ast – Jovet-Ast (1967). Sabah.

***Coluraimperfecta*** Steph. – Jovet-Ast (1954), Eggers et al. (1998). Pahang, Sarawak.

***Colurainflata*** K.I.Goebel – Müller and Schäfer-Verwimp (1999). Perak.

***Colurainuii*** Horik. – Cheah (2017), Sarimi et al. (2021), Pesiu et al. (in press). Terengganu, Pahang.

***Colurakarstenii*** K.I.Goebel – Jovet-Ast (1954), Lee et al. (2013), Sangrattanaprasert et al. (2018). Kedah, Pahang, Johor, Sabah.

***Coluraleratii*** (Steph.) Steph. – Jovet-Ast (1967 as *C.apiculata*). Sabah.

***Coluramaxima*** Jovet-Ast – Pócs et al. (2020). Sabah.

***Coluramosenii*** Steph. – Pócs and Lee (2016). Kelantan.

***Coluraornata*** K.I.Goebel – Schiffner (1893 as *Lejeuneaornata*,1898 as *Colurolejeuneaornata*), Jovet-Ast (1954, 1967), Mizutani (1966), Kitagawa and Kodama (1974), Wolseley et al. (1996), Pócs et al. (2020). Penang, Pahang, Negeri Sembilan, Sarawak, Sabah.

***Colurapluridentata*** Jovet-Ast – Mizutani (1970). Sabah.

***Colurasigmoidea*** Sangratt., Chantanaorr. et R.L.Zhu – Sangrattanaprasert et al. (2019). Pahang.

***Coluraspeciosa*** Jovet-Ast – Pócs and Lee (2016). Kelantan.

***Colurastrophiolata*** Jovet-Ast – Jovet-Ast (1976). Kedah.

***Colurasuperba*** (Mont.) Steph. – Jovet-Ast (1967), Pócs et al. (2020). Sabah.

***Coluratenuicornis*** (A.Evans) Steph. – Jovet-Ast (1954, 1976), Mizutani (1970), Tixier (1980a), Pócs et al. (2020). Pahang, Johor, Sabah.

***Coluravalida*** Jovet-Ast – Sangrattanaprasert et al. (2019). Pahang.

***Coluraverdoornii*** Herzog et Jovet-Ast – Jovet-Ast (1954, 1976), Tixier (1980a), Sangrattanaprasert et al. (2019), Pócs et al. (2020). Kedah, Pahang, Johor, Sabah.

***Conoscyphustrapezioides*** (Sande Lac.) Schiffn. – De Notaris (1874 as *Diploscyphusborneensis*), Herzog (1950 as *C.inflexifolius*), Inoue (1967a), Kitagawa and Kodama (1974), Piippo (1989), Piippo et al. (2014). Pahang, Malacca, Sarawak, Sabah.

***Cryptolophocoleaciliolata*** (Nees) L.Söderstr., Crand.-Stotl., Stotler et Váňa – Kitagawa and Kodama (1974 as *Lophocoleaciliolata*), Piippo (1989 as *Chiloscyphusciliolatus*), Inoue (1967a, 1968b both as *L.ciliolata*), Cheah (2017 as *C.ciliolatus*). Pahang, Sabah.

***Cryptolophocoleacostata*** (Nees) L.Söderstr. – Herzog (1950 as *Lophocoleacostata*), Inoue (1967a as *L.costata*), Kitagawa and Kodama (1974 as *L.costata*), Piippo (1989 as *Chiloscyphuscostatus*), Akiyama et al. (2001 as *L.costata*). Pahang, Sarawak, Sabah.

***Cryptolophocolealevieri*** (Schiffn.) L.Söderstr. – Piippo (1989 as *Chiloscyphusparoicus*). Sabah.

***Cryptolophocoleamassalongoana*** (Schiffn.) L.Söderstr. – Pocock et al. (1984 as *Lophocoleamassalongoana*). Pahang.

***Cyathodiumfoetidissimum*** Schiffn. – Lang (1905). Perak.

***Cyathodiumcavernarum*** Kunze – Lee and Gradstein (2021). Kelantan.

***Cylindrocoleatagawae*** (N.Kitag.) R.M.Schust. – Kitagawa (1971 as *Cephaloziellatagawae*). Penang.

***Dactylophorellamuricata*** (Gottsche) R.M.Schust. – Mizutani (1970 as *Drepanolejeuneamuricata*), Yong and Chan (2017). Perak, Sabah.

***Denotarisialinguifolia*** (De Not.) Grolle – De Notaris (1874 as *Plagiochilalinguifolia*), Inoue (1968b as *Jamesoniellaovifolia*), Amakawa (1969a as J.flexicaulisf.affinis), Kitagawa (1970 as *J.pulchra*), Grolle (1971), Kitagawa and Kodama (1973), Kürschner (1990), Váňa (1991c), Akiyama et al. (2001). Pahang, Sarawak, Sabah.

***Dinckleriasingularis*** (Schiffn.) M.A.M.Renner, Schäf.-Verw. et Heinrichs – Inoue (1989 as *Plagiochilasingularis*), Cheah (2017 as *P.singularis*). Pahang, Sabah.

***Diplasiolejeuneacavifolia*** Steph. – Tixier (1971 as *D.brachyclada*, 1980a as *D.javanica*), Mizutani (1966 as *D.javanica*, 1970 as *D.brachyclada*), Kitagawa and Kodama (1974), Dong et al. (2012), Pócs et al. (2020). Perak, Pahang, Sabah.

***Diplasiolejeuneacobrensis*** Steph. – Mizutani (1970 as *D.incurvata*). Sabah.

***Diplasiolejeuneajovet-astiae*** Grolle – Grolle (1966), Kitagawa and Kodama (1974), Tixier (1980a), Dong et al. (2012), Pócs et al. (2020). Pahang, Sarawak, Sabah.

***Diplasiolejeunealongilobula*** Herzog – Herzog (1950). Sarawak.

***Diplasiolejeuneapatelligera*** Herzog – Mizutani (1970 as *D.neobrachyclada*), Tixier (1980a, also as *D.neobrachyclada*), Dong et al. (2012). Pahang, Sabah.

***Diplasiolejeuneaunidentata*** (Lehm. et Lindenb.) Schiffn. – Pócs and Lee (2016 as *D.rudolphiana*). Kelantan.

***Diplophyllumkinabaluense*** Furuki et Suleiman – Furuki and Suleiman (2016). Sabah.

***Diplophyllumnanum*** Herzog – Müller and Schäfer-Verwimp (1999). Pahang.

***Drepanolejeuneaangustifolia*** (Mitt.) Grolle – Herzog (1950 as *D.tenuis*), Tixier (1971 as *D.tenuis*), Kamimura (1974 as *D.tenuis*). Pahang, Sarawak, Sabah.

***Drepanolejeuneabischlerae*** (Grolle) Grolle et R.L.Zhu – Grolle and Zhu (2000). Sabah.

***Drepanolejeuneaciliata*** Mizut. – Mizutani (1970), Kamimura (1974). Sabah.

***Drepanolejeuneacyclops*** (Sande Lac.) Grolle et R.L.Zhu – Herzog (1943 as *Rhaphidolejeuneacyclops*), Mizutani (1966, 1970 both as *R.cyclops*), Bischler (1968 as *R.cyclops*). Selangor, Sarawak, Sabah.

***Drepanolejeuneadactylophora*** (Nees, Lindenb. et Gottsche) J.B.Jack et Steph. – Herzog (1934, 1950), Mizutani (1966, 1970), Kamimura (1974), Kitagawa and Kodama (1974), Pócs et al. (2020), Pesiu et al. (2021). Terengganu, Johor, Sarawak, Sabah.

***Drepanolejeuneaelegans*** Herzog – Herzog (1936). Sabah.

***Drepanolejeuneafissicornua*** Steph. – Mizutani (1990), Pócs et al. (2014, 2020), Pócs and Lee (2016). Kelantan, Pahang, Selangor, Sabah.

***Drepanolejeuneafoliicola*** Horik. – Eggers (2006). Pahang.

***Drepanolejeuneaintermedia*** Zwickel – Grolle (1976). Sabah.

***Drepanolejeunealevicornua*** Steph. – Tixier (1971 [‘*laevicornua*’]), Mizutani (1990), Eggers et al. (1998), Pócs et al. (2014), Pesiu et al. (2021, in press). Selangor, Pahang, Terengganu, Sarawak.

***Drepanolejeunealongicornua*** (Herzog) Mizut. – Mizutani (1970 as D.levicornuavar.longicornua, 1990), Kitagawa and Kodama (1974 as D.levicornuavar.longicornua), Pócs et al. (2020), Pesiu et al. (2021). Penang, Terengganu, Sarawak, Sabah.

***Drepanolejeunealongicruris*** (Steph.) Grolle et R.L.Zhu – Herzog (1943 as *Rhaphidolejeunealongicruris*), Bischler (1968 as *R.longicruris*), Kitagawa and Kodama (1974 as *R.longicruris*), Grolle and Zhu (2000). Sabah.

***Drepanolejeuneanymanii*** Steph. – Mizutani (1990). Sabah.

***Drepanolejeuneaobliqua*** Steph. – Herzog (1939), Mizutani (1970). Johor, Sabah.

***Drepanolejeuneaobtriangulata*** T.Kodama – Kodama (1976), Pócs et al. (2014). Selangor, Sabah.

***Drepanolejeuneapentadactyla*** (Mont.) Steph. – Herzog (1934 as D.micholitziiand itsvar.angustissima), Tixier (1974 as *D.micholitzii*, 1980a as D.micholitziivar.brevifolia), Mizutani (1966, 1970 both as *D.micholitzii*), Kitagawa and Kodama (1974 as *D.micholitzii*), Pócs et al. (2014, 2020), Pesiu et al. (2021, in press). Kedah, Selangor, Pahang, Terengganu, Johor, Sarawak, Sabah.

***Drepanolejeuneapentadactyla*** var. ***dactylophoroides*** (Herzog) Pócs – Herzog (1934 as D.micholitziivar.dactylophoroides). Sabah.

***Drepanolejeuneapleiodictya*** Herzog – Tixier (1980a). Pahang.

***Drepanolejeuneaserricalyx*** Herzog – Pócs et al. (2020). Sabah.

***Drepanolejeuneaspicata*** (Steph.) Grolle et R.L.Zhu – Mizutani (1966 as *Rhaphidolejeuneaspicata*), Bischler (1968 as *R.spicata*), Grolle and Zhu (2000), Pócs et al. (2014), Pesiu et al. (2021, in press). Pahang, Terengganu, Sarawak, Sabah.

***Drepanolejeuneaspinosocornuta*** Steph. – Tixier (1980a). Pahang.

***Drepanolejeuneatenera*** K.I.Goebel – Herzog (1934 as D.teneravar.genuinaandf.goebelii, 1952b), Mizutani (1966, 1970), Tixier (1971, 1974, 1980a as D.teneraf.goebelii). Kitagawa and Kodama (1974), Pócs et al. (2020). Kedah, Pahang, Sarawak, Sabah.

***Drepanolejeuneaternatensis*** (Gottsche) Schiffn. – Herzog (1950, also as D.ternatensisvar.lancispina), Mizutani (1966, 1970), Kamimura (1974), Cheah (2017), Pócs et al. (2020), Pesiu et al. (2021, in press). Pahang, Terengganu, Sarawak, Sabah.

***Drepanolejeuneateysmannii*** (Gottsche) Steph. – Herzog (1950), Mizutani (1966, 1970), Inoue (1968b), Kamimura (1974, 1975), Kitagawa and Kodama (1974), Kürschner (1990), Pócs et al. (2020). Pahang, Sarawak, Sabah.

***Drepanolejeuneathwaitesiana*** (Mitt.) Steph. – Goebel (1930 [‘*Thwaitesii*’]), Mizutani (1966, 1970, 1990), Tixier (1971, 1974, 1980a), Kitagawa and Kodama (1974), Pócs et al. (2020), Pesiu et al. (2021, in press). Kedah, Perak, Selangor, Pahang, Terengganu, Sabah.

***Drepanolejeuneathwaitesiana*** var. ***zhengii*** R.L.Zhu – Zhu and So (2001). Pahang.

***Drepanolejeuneatricornua*** Herzog – Mizutani (1970, 1990), Pócs et al. (2014, 2020). Selangor, Pahang, Sarawak, Sabah.

***Drepanolejeuneavesiculosa*** (Mitt.) Steph. – Herzog (1950 as D.vesiculosaf.robusta), Inoue (1967a, 1968b), Mizutani (1966, 1970), Kamimura (1974), Kitagawa and Kodama (1974), Kürschner (1990), Lee et al. (2013), Pócs et al. (2020). Perak, Pahang, Sarawak, Sabah.

***Dumortierahirsuta*** (Sw.) Nees – Johnson (1958), Akiyama et al. (2001), Ludwiczuk and Asakawa (2010). Pahang, Sabah.

***Dumortierahirsuta*** subsp. ***nepalensis*** (Taylor) R.M.Schust. – Campbell (1915 as *D.trichocephala*, 1918 as *D.calcicola*), Evans (1919 as *D.nepalensis*), Kitagawa and Kodama (1974 as *D.nepalensis*). Perak, Selangor, Sarawak, Sabah.

***Eotrichocoleafurukii*** T.Katag. – Katagiri et al. (2012). Sabah.

***Frullaniaalstonii*** Verd. – Verdoorn (1932b), Hattori (1976). Johor, Sabah.

***Frullaniaapiculata*** (Reinw., Blume et Nees) Nees – Verdoorn (1932a), Herzog (1950), Inoue (1967a, 1968b), Tixier (1974, 1980a), Kamimura (1974, 1975), Kitagawa and Kodama (1974), Hattori (1975c, 1976), Kodama (1976), Pócs et al. (2020), Pesiu et al. (2021). Kedah, Terengganu, Pahang, Selangor, Sarawak, Sabah.

***Frullaniaapiculata*** var. ***goebelii*** Schiffn. – Verdoorn (1934b), Herzog (1950), Tixier (1971). Pahang, Sarawak, Sabah.

***Frullaniaarmatifolia*** Verd. – Hattori (1976 as *F.altemammillata*). Sabah.

***Frullaniaarmitiana*** Steph. – Kamimura (1974). Sabah.

***Frullaniabenjaminiana*** Inoue – Inoue (1967a,b), Hattori (1975a). Pahang.

***Frullaniaberthoumieui*** Steph. – Hattori (1976). Sabah.

***Frullaniabrotheri*** Steph. – Stephani (1894), Schiffner (1898), Bonner (1965), Hattori (1975a,c, 1976, 1980), Kürschner (1990), Sukkharak (2018). Perlis, Perak, Selangor, Pahang, Malacca, Johor, Sabah.

***Frullaniaclaviloba*** Steph. – Verdoorn (1930, 1932b), Herzog (1950), Kamimura (1974), Kitagawa and Kodama (1974), Hattori (1974b, 1975b,c, 1976). Selangor, Johor, Sarawak, Sabah.

***Frullaniaclemensiana*** Verd. – Verdoorn (1932a), Hattori and Kamimura (1971 as *Steereamastigophoroides*), Hattori (1976 as *S.clemensiana*). Sabah.

***Frullaniacordistipula*** (Reinw., Blume et Nees) Nees – Kamimura (1974, 1975). Sabah.

***Frullaniaericoides*** (Nees) Mont. – Verdoorn (1929 as *F.squarrosa*), Hattori (1975a, 1976). Penang, Pahang, Sabah.

***Frullaniafallax*** Gottsche – Verdoorn (1930). Pahang.

***Frullaniagaudichaudii*** (Nees et Mont.) Nees et Mont. – Verdoorn (1930, 1932a), Herzog (1950), Kitagawa and Kodama (1974), Hattori (1975c, 1976), Cheah (2017). Selangor, Pahang, Sarawak, Sabah.

***Frullaniagracilis*** (Reinw., Blume et Nees) Nees – Verdoorn (1932a as *F.minor*), Herzog (1950), Kamimura (1974, 1975), Kitagawa and Kodama (1974), Hattori (1975a, 1975c as *F.minor*, 1976), Kürschner (1990). Selangor, Pahang, Malacca, Johor, Sarawak, Sabah.

***Frullaniagrandistipula*** Lindenb. – Verdoorn (1932a), Tixier (1971), Hattori (1976). Pahang, Sabah.

***Frullaniahasskarliana*** Lindenb. – Verdoorn (1932a), Hattori (1973, 1975a, 1976 as F.hasskarlianavar.integribracteata), Kitagawa and Kodama (1974). Pahang, Sabah.

***Frullaniahottana*** S.Hatt. – Hattori (1976, 1986). Sarawak.

***Frullaniahypoleuca*** Nees – Hattori (1976), Kamimura (1974). Sarawak, Sabah.

***Frullaniaintegristipula*** (Nees) Nees – Verdoorn (1930, 1932a), Herzog (1950), Kamimura (1974), Kitagawa and Kodama (1974), Tixier (1974, 1980a), Hattori (1976, 1980). Kedah, Pahang, Johor, Sarawak, Sabah.

***Frullaniaintermedia*** (Reinw., Blume et Nees) Nees – Verdoorn (1930, 1934b), Herzog (1950), Kitagawa and Kodama (1974), Hattori (1975c, 1976 as F.intermediavar.submorokensis and F.intermediaf.billardieriana, 1980), Wolseley et al. (1996). Penang, Perak, Kelantan, Negeri Sembilan, Johor, Sarawak, Sabah.

***Frullaniajunghuhniana*** Gottsche – Verdoorn (1932a, 1934b), Hattori (1974c, 1976), Kürschner (1990). Sabah.

***Frullaniajunghuhniana*** var. ***bisexualis*** S.Hatt. – Hattori (1976), Kodama (1976). Sabah.

***Frullaniajunghuhniana*** var. ***tenella*** (Sande Lac.) Grolle et S.Hatt. – Kamimura (1974 as *F.perversa*), Tixier (1974 as *Neohattoriaperversa*), Hattori (1975a, 1976 as F.junghuhnianavar.minutissima and *F.perminuta*). Kedah, Pahang, Sabah.

***Frullaniameijeri*** S.Hatt. – Hattori (1974c, 1976). Sabah.

***Frullaniameyeniana*** Lindenb. – Verdoorn (1934b), Tixier (1971), Hattori (1975c). Pahang, Sabah.

***Frullaniamizutanii*** Kamim. et S.Hatt. – Hattori and Kamimura (1973), Kamimura (1974), Hattori (1976, 1978), Kodama (1976). Sabah.

***Frullaniamoniliata*** (Reinw., Blume et Nees) Mont. – Verdoorn (1932a as F.moniliatasubsp.breviramea), Hattori (1972, 1976 both as F.tamariscivar.breviramea). Sabah.

***Frullaniamonocera*** (Hook.f. et Taylor) Gottsche, Lindenb. et Nees – Hattori (1976 as *F.hampeana*). Sabah.

***Frullanianepalensis*** (Spreng.) Lehm. et Lindenb. – Verdoorn (1932b). Pahang.

***Frullanianigricaulis*** (Reinw., Blume et Nees) Nees – Verdoorn (1930, 1932a), Hattori (1975a). Pahang, Sabah.

***Frullanianodulosa*** (Reinw., Blume et Nees) Nees – Verdoorn (1930, 1932a, 1934b), Herzog (1950), Inoue (1968b), Kitagawa and Kodama (1974), Hattori (1975c, 1980). Perak, Selangor, Pahang, Johor, Sarawak, Sabah.

***Frullanianotarisii*** Steph. – Verdoorn (1934b), Hattori (1974c, 1976), Kitagawa and Kodama (1974), Tixier (1974). Kedah, Sarawak, Sabah.

***Frullaniaobscura*** (Sw.) Mont. – Hattori (1975a as *F.wallichiana*). Pahang.

***Frullaniaocellata*** S.Hatt. et Kamim. – Hattori and Kamimura (1973), Kamimura (1974). Sabah.

***Frullaniaorientalis*** Sande Lac. – Verdoorn (1937), Hattori (1976). Pahang, Sabah.

***Frullaniaornithocephala*** (Reinw., Blume et Nees) Nees – Verdoorn (1934b), Hattori (1975a, 1976 as F.ornithocephalaf.magnilobulaandf.retusa, 1980). Pahang, Sabah.

***Frullaniapapillata*** Steph. – Kamimura (1974, 1975). Sabah.

***Frullaniapapillilobula*** S.Hatt. – Hattori (1975b, 1976). Sabah.

***Frullaniapolyptera*** Taylor – Hattori (1975a). Pahang.

***Frullaniapullei*** Verd. – Tixier (1974). Kedah.

***Frullaniaramuligera*** (Nees) Mont. – Verdoorn (1932a, 1934b), Hattori (1972), Pócs et al. (2020). Sabah.

***Frullaniarecurvistipula*** S.Hatt. – Hattori (1975a, 1986). Pahang.

***Frullaniareﬂexistipula*** Sande Lac. – Verdoorn (1932a), Hattori (1975a as *F.philippinensis*, 1976). Pahang, Sabah.

***Frullaniarepandistipula*** Sande Lac. subsp. ***spinibractea*** S.Hatt. – Hattori (1975c, 1976 as F.repandistipulasubsp.spinifera), Pesiu et al. (in press). Terengganu, Sabah.

***Frullaniasabahana*** S.Hatt. – Hattori (1976, 1980). Sarawak, Sabah.

***Frullaniasarawakensis*** S.Hatt. – Hattori (1976, 1980). Sarawak.

***Frullaniaserrata*** Gottsche – Verdoorn (1932b, 1934b), Herzog (1950), Kamimura (1974, 1975), Kitagawa and Kodama (1974), Hattori (1975a, 1976 as F.serrataf.crispulodentata), Ludwiczuk and Asakawa (2010). Pahang, Johor, Sarawak, Sabah.

***Frullaniasinuata*** Sande Lac. – Verdoorn (1932b, 1934b), Hattori (1975a, 1980). Johor, Pahang.

***Frullaniasublignosa*** Steph. – Stephani (1910 as *F.borneensis*), Verdoorn (1930), Hattori (1976). Sarawak.

***Frullaniasubnigricaulis*** S.Hatt. – Hattori (1974b, 1975a, 1976). Pahang, Sabah.

***Frullaniasubocellata*** S.Hatt. – Pócs et al. (2014). Pahang.

***Frullaniaternatensis*** Gottsche – Verdoorn (1930, 1932a, 1934b), Herzog (1950), Tixier (1971, 1974), Kamimura (1974, 1975), Kitagawa and Kodama (1974), Hattori (1975c). Kedah, Pahang, Sarawak, Sabah.

***Frullaniaternatensis*** var. ***nonappendiculata*** S.Hatt. – Hattori (1976). Sarawak, Sabah.

***Frullaniatogashiana*** S.Hatt. – Hattori (1975a). Pahang.

***Frullaniatricarinata*** Sande Lac. – Kamimura (1974). Sabah.

***Frullaniatrichodes*** Mitt. – Verdoorn (1930, 1934b as *F.picta*, *F.tenuicaulis* and *F.vethii*), Herzog (1950 as *F.picta*, *F.tenuicaulis* and *F.vethii*), Kitagawa (1969c as *F.picta*), Kamimura (1974, 1975 both as *F.tenuicaulis*), Kitagawa and Kodama (1974 as *F.tenuicaulis*), Tixier (1974 as *F.picta*), Hattori (1976 as F.vethiif.acutiloba), Pócs et al. (2014), Cheah (2017). Kedah, Penang, Pahang, Johor, Sarawak, Sabah.

***Frullaniavaginata*** (Sw.) Nees – Hattori (1975a). Pahang.

***Frullaniavenusta*** S.Hatt. – Hattori (1974b, 1976). Sabah.

***Gottscheliaschizopleura*** (Spruce) Grolle – Inoue (1967a as *Jamesoniellamicrophylla*), Amakawa (1969a as *J.microphylla*), Váňa (1991c). Pahang, Sabah.

***Gymnomitrionincompletum*** (Gottsche) Váňa – Kitagawa (1973 as G.laceratumvar.borneense), Kitagawa and Kodama (1973 as G.laceratumvar.borneense), Váňa (1991a). Sabah.

***Gymnomitrionrevolutum*** (Nees) H.Philib. – Kitagawa (1967 as *Marsupellarevoluta*), Kitagawa and Kodama (1973 as *M.revoluta*), Váňa (1991a as *M.revoluta*), Akiyama et al. (2001 as *M.revoluta*). Sabah.

***Gymnomitrionsubintegrum*** (S.W.Arnell) Váňa – Kitagawa (1967 as *Marsupellaintegra*), Váňa (1991a*M.subintegra*). Sabah.

***Haplomitriumblumei*** (Nees) R.M.Schust. – Bartholomew-Began (1991). Pahang.

***Herbertusaduncus*** (Dicks.) Gray – Kürschner (1990 as *H.dicranus* [‘*dicrananus*’]), Juslén (2006 as *H.dicranus* and *H.longispinus*), Cheah (2017 as *H.dicranus*). Pahang, Sabah.

***Herbertusceylanicus*** (Steph.) Abeyw. – Juslén (2006). Pahang.

***Herbertuspilifer*** (Steph.) H.A.Mill. – Juslén (2006). Sarawak.

***Herbertusramosus*** (Steph.) H.A.Mill. – Kamimura (1975 [‘*Herbertaramosa*’]). Sabah.

***Herbertussendtneri*** (Nees) Lindb. – Kürschner (1990 as *H.armitanus* and *H.circinatus*), Akiyama et al. (2001 as *H.armitanus*), Juslén (2006 as *H.circinatus*), Cheah (2017 as *H.armitanus*). Pahang, Sabah.

***Heteroscyphusacutangulus*** (Schiffn.) Schiffn. – Inoue (1968b as *Chiloscyphusacutangulus*). Pahang.

***Heteroscyphusargutus*** (Reinw., Blume et Nees) Schiffn. – Herzog (1950 as *Chiloscyphusargutus*), Kitagawa (1969c), Tixier (1971 as *C.argutus*), Kitagawa and Kodama (1974 as *C.argutus*), Inoue (1981), Piippo (1989), Wolseley et al. (1996). Penang, Pahang, Negeri Sembilan, Sarawak, Sabah.

***Heteroscyphusaselliformis*** (Reinw., Blume et Nees) Schiffn. – Mitten and Wright (1894 as *Chiloscyphusaselliformis*), Herzog (1950 as C.aselliformisvar.neesii), Inoue (1967a as *C.aselliformis*), Kitagawa and Kodama (1974), Piippo (1989), Kürschner (1990 as *C.aselliformis*), Ludwiczuk and Asakawa (2010). Pahang, Sarawak, Sabah.

***Heteroscyphusbalnetii*** (Herzog) Grolle – Piippo (1989). Sabah.

***Heteroscyphuscoalitus*** (Hook.) Schiffn. – Herzog (1950 as *Chiloscyphuscommunis*), Inoue (1967a, 1968b both as *C.communis*), Kitagawa (1971 as *H.communis*), Tixier (1971 as *C.communis*), Kitagawa and Kodama (1974 as *C.communis*), Kamimura (1975 as *H.bescherellei*), Piippo (1989). Penang, Pahang, Sarawak, Sabah.

***Heteroscyphusdiestianus*** (Sande Lac.) Piippo – Kitagawa and Kodama (1974 as *Chiloscyphusdiestianus*), Piippo (1989), Akiyama et al. (2001). Sarawak, Sabah.

***Heteroscyphusintegerrimus*** (Schiffn.) Gradst. et G.E.Lee – Pócs et al. (2014 as *Chiloscyphusintegerrimus*). Pahang.

***Heteroscyphusiwatsukii*** (S.Hatt.) Piippo – Hattori (1964 as *Saccogynidiumiwatsukii*), Piippo (1989). Sabah.

***Heteroscyphusparvulus*** (Schiffn.) Schiffn. – Kitagawa and Kodama (1974 as *Chiloscyphusparvulus*). Sabah.

***Heteroscyphussplendens*** (Lehm. et Lindenb.) Grolle – De Notaris (1874 as *Chiloscyphusdecurrens* and *C.densifolius*), Herzog (1950, 1952b both as *C.decurrens*), Inoue (1967a, 1968b, both as *C.decurrens*), Kitagawa (1969c as *C.decurrens*), Kitagawa and Kodama (1974 as *C.decurrens*), Tixier (1974 as *C.decurrens*), Piippo (1989), Kürschner (1990), Akiyama et al. (2001). Kedah, Penang, Pahang, Sarawak, Sabah.

***Heteroscyphussucculentus*** (Gottsche) Schiffn. – De Notaris (1874 as *Chiloscyphusconcinnus*), Stephani (1907 as *C.succulentus*), Herzog (1950 as *C.succulentus*), Kitagawa (1973 as *C.succulentus*), Kitagawa and Kodama (1974 as *C.succulentus*), Piippo (1989). Penang, Sarawak, Sabah.

***Heteroscyphustridentatus*** (Sande Lac.) Grolle – Cheah (2017). Pahang.

***Heteroscyphuswettsteinii*** (Schiffn.) Schiffn. – Kitagawa and Kodama (1974 as *Chiloscyphuswettsteinii*), Müller and Schäfer-Verwimp (1999), Cheah (2017). Perak, Pahang, Sabah.

***Hygrolembidiumboschianum*** (Sande Lac.) R.M.Schust. – Grolle (1967). Malacca.

***Isotachisjaponica*** Steph. – Inoue (1967a). Pahang.

***Jackiellaangustifolia*** Herzog – Herzog (1950, 1952a), Váňa (1992). Sarawak, Sabah.

***Jackiellajavanica*** Schiffn. – Herzog (1950), Inoue (1967a), Kitagawa (1971), Váňa (1992). Penang, Pahang, Sarawak, Sabah.

***Jackiellasingapurensis*** Schiffn. – Kitagawa (1969c, 1981). Penang.

***Jenseniadecipiens*** (Mitt.) Grolle – Herzog (1950 as *Makednothallusobtusidens*), Kitagawa and Kodama (1974 as *J.zollingeri*), Grolle and Piippo (1986), Akiyama et al. (2001), Forrest et al. (2005). Sarawak, Sabah.

***Jubulajavanica*** Steph. – Herzog (1950 as J.hutchinsiaesubsp.javanica), Kitagawa and Kodama (1974 as J.hutchinsiaesubsp.javanica), Váňa and Piippo (1989b as J.hutchinsiaesubsp.javanica), Pätsch et al. (2010 as J.hutchinsiaesubsp.javanica). Pahang, Sarawak, Sabah.

***Kurziaabbreviata*** Mizut. – Mizutani (1974), Hürlimann (1985), Cheah (2017). Pahang, Sarawak, Sabah.

***Kurziaabietinella*** (Herzog) Grolle – Herzog (1950 as *Lepidoziaabietinella*), Kitagawa and Kodama (1973), Mizutani (1974), Kürschner (1990), Cheah (2017). Pahang, Sarawak, Sabah.

***Kurziaborneensis*** Mizut. – Mizutani (1974), Kamimura (1975), Akiyama et al. (2001), Cheah (2017). Pahang, Sabah.

***Kurziageniculata*** Mizut. – Mizutani (1974), Cheah (2017). Pahang, Sabah.

***Kurziagonyotricha*** (Sande Lac.) Grolle – Herzog (1952b as *Lepidoziagonyotricha*), Kitagawa (1969c), Mizutani (1974), Akiyama et al. (2001). Penang, Sarawak, Sabah.

***Kurzialineariloba*** Mizut. – Mizutani (1974), Kürschner (1990), Cheah (2017). Pahang, Sabah.

***Kymatocalyxrhizomaticus*** (Herzog) Gradst. et Váňa – Herzog (1950, 1952a both as *Stenorrhipisrhizomatica*), Váňa (1991c as *S.rhizomatica*). Sarawak.

***Leiomitramerrillana*** (Steph.) T.Katag. – Kitagawa (1973 as *Trichocoleamerrillana*), Akiyama et al. (2001 as *T.merrillana*), Katagiri and Deguchi (2012). Pahang, Sabah.

***Lejeuneaadpressa*** Nees – Inoue (1968b as *L.boninensis*), Kitagawa (1971 as *L.borneensis*), Mizutani (1978 as *L.borneensis*), Wolseley et al. (1996 as *L.caespitosa*), Zhu and So (2001 as *L.anisophylla*), Lee (2013 as *L.anisophylla*), Pesiu et al. (2021, in press). Perlis, Langkawi, Perak, Penang, Selangor, Kuala Lumpur, Pahang, Negeri Sembilan, Terengganu, Johor, Sarawak, Sabah.

***Lejeuneaalata*** Gottsche – Mizutani (1970 as *L.mitracalyx*), Kitagawa and Kodama (1974 as *L.mitracalyx*), Lee (2013). Pahang, Sabah.

***Lejeuneaalbescens*** (Steph.) Mizut. – Eifrig (1937 as *Taxilejeuneaalbescens*), Inoue (1967a as *T.albescens*), Mizutani (1970), Hattori and Kamimura (1971), Lee (2013). Pahang, Sabah.

***Lejeuneaapiculata*** Sande Lac. – Mizutani (1966 as *Prionolejeuneaungulata*, 1970 as *Stenolejeuneaapiculata*), Tixier (1971 as *S.apiculata*), Kitagawa and Kodama (1974 as *P.ungulata*), Lee (2013), Cheah (2017). Pahang, Sabah.

***Lejeuneacocoes*** Mitt. – Mizutani (1963), Kamimura (1974), Lee (2013), Pesiu et al. (in press). Perlis, Pahang, Terengganu, Sarawak, Sabah.

***Lejeuneacompacta*** (Steph.) Steph. – Lee (2013). Sabah.

***Lejeuneacompressiuscula*** (Steph.) G.E.Lee et Heinrichs – Eifrig (1937 as *Taxilejeuneacompressiuscula*), Tixier (1971 as *T.compressiuscula*). Pahang, Johor.

***Lejeuneacontracta*** Mizut. – Mizutani (1970), Kitagawa and Kodama (1974), Lee (2013). Sabah.

***Lejeuneaconvexiloba*** M.L.So et R.L.Zhu – Sarimi et al. (2021). Sabah.

***Lejeuneadimorpha*** Kodama – Kodama (1976), Lee (2013), Pócs et al. (2014). Selangor, Kelantan, Pahang, Sabah.

***Lejeuneadipterota*** (Eifrig) G.E.Lee – Lee (2013). Sabah.

***Lejeuneadiscreta*** Lindenb. – Mizutani (1970), Kitagawa and Kodama (1974), Tixier (1980a as *Xylolejeunealongiloba*), Lee et al. (2011b), Lee (2013). Perlis, Perak, Pahang, Sabah.

***Lejeuneaeifrigii*** Mizut. – Mizutani (1970), Lee et al. (2011b), Lee (2013). Pahang, Sabah.

***Lejeuneaexilis*** (Reinw., Blume et Nees) Grolle – Mizutani (1970), Pócs et al. (2020). Sabah.

***Lejeuneaexilis*** var. ***abnormis*** (Herzog) G.E.Lee – Lee (2013). Kelantan, Pahang.

***Lejeuneaﬂava*** (Sw.) Nees – Inoue (1967a), Mizutani (1970), Kitagawa and Kodama (1974), Lee (2013), Pócs et al. (2020). Kedah, Penang, Perak, Selangor, Kelantan, Pahang, Sabah.

***Lejeunea ﬂavida*** Mitt. – Kamimura (1974 as *Pycnolejeuneaflavida*), Inoue (1967a as *P.flavida*). Pahang, Sabah.

***Lejeuneafleischeri*** (Steph.) Mizut. – Lee (2013). Perak, Pahang.

***Lejeuneagradsteinii*** G.E.Lee, Damanhuri et Latiff – Lee et al. (2011a), Lee (2013), Pócs and Lee (2016). Pahang, Sabah.

***Lejeuneakinabalensis*** Mizut. – Mizutani (1970), Lee (2013). Sabah.

***Lejeunealeratii*** (Steph.) Mizut. – Eifrig (1937 as *Taxilejeuneapatersonii*). Sabah.

***Lejeunealumbricoides*** (Nees) Nees – Eifrig (1937 as *Taxilejeunealumbricoides*), Mizutani (1970), Kitagawa and Kodama (1974), Tixier (1980a), Lee (2013). Pahang, Sabah.

***Lejeuneamalaysiana*** G.E.Lee et Pócs - Lee et al. (in press). Pahang.

***Lejeuneamicholitzii*** Mizut. – Mizutani (1970), Lee (2013), Pócs et al. (2014, 2020), Pesiu et al. (2021). Langkawi, Selangor, Pahang, Negeri Sembilan, Kelantan, Terengganu, Sarawak, Sabah.

***Lejeuneamicroloba*** Taylor – Mizutani (1970 as *L.chalmersii*), Lee (2013). Sabah.

***Lejeuneamimula*** Hürl. – Tixier (1971 as *Taxilejeunealuteola*), Mizutani (1970 as *L.luteola*), Kitagawa and Kodama (1974 as *L.luteola*), Lee (2013). Pahang, Sabah.

***Lejeuneamizutanii*** Grolle – Mizutani (1970 as *L.zollingeri*), Lee (2013). Sabah.

***Lejeuneapapilionacea*** Prantl – Mizutani (1966 as *L.infestans* and *L.pterota*, 1970 as *L.herzogii* and *L.infestans*, 1972b as *L.diversitexta*), Kitagawa and Kodama (1974 as *L.diversitexta*), Zhu and Grolle (2001), Lee (2013), Pócs et al. (2020). Kedah, Langkawi, Selangor, Kelantan, Pahang, Sarawak, Sabah.

***Lejeuneapatersonii*** (Steph.) Steph. – Inoue (1967a), Lee (2013). Kuala Lumpur, Kelantan, Pahang, Johor.

***Lejeuneapatriciae*** Schäf.-Verw. – Tixier (1971 as *L.pilifera*), Schäfer-Verwimp (2001, 2006), Lee (2013). Penang, Perak, Kelantan, Pahang.

***Lejeuneapectinella*** Mizut. – Mizutani (1970), Lee (2013). Sabah.

***Lejeuneapulchriflora*** (Pearson) G.E.Lee, Bechteler, Pócs, Schäf.-Verw. et Heinrichs – Lee (2013 as *L.tamaspocsii*), Lee and Gradstein (2013 as *L.tamaspocsii*), Lee et al. (2016). Kelantan, Pahang, Sabah.

***Lejeuneasordida*** (Nees) Nees – Eifrig (1937 as *Taxilejeuneasordida*), Mizutani (1970), Lee et al. (2011a, 2011b), Lee (2013). Perlis, Langkawi, Kedah, Penang, Perak, Selangor, Kelantan, Pahang, Johor, Sarawak, Sabah.

***Lejeuneastenodentata*** M.A.M.Renner et Pócs – Herzog (1950 as *Crossotolejeuneaborneensis*). Mizutani (1966, 1970 both as *C.borneensis*, 1972a as *Drepanolejeuneadentata*). Sarawak, Sabah.

***Lejeuneastephaniana*** Mizut. – Mizutani (1966, 1970), Lee (2013). Sabah.

***Lejeuneathallophora*** (Eifrig) Gradst. – Zhu et al. (2018b). Sabah.

***Lejeuneatrinitensis*** Lindenb. – Reiner-Drehwald and Grolle (2012). Johor.

***Lejeuneatuberculosa*** Steph. – Lee (2013). Kedah, Penang, Perak, Selangor, Pahang, Sabah.

***Lejeuneaumbilicata*** (Nees) Nees – Herzog (1950 as *Taxilejeuneaumbilicata*), Mizutani (1970), Kitagawa and Kodama (1974), Tixier (1980a), Grolle (1988 as *L.cuculliflora*), Lee (2013). Perak, Selangor, Kelantan, Pahang, Johor, Sarawak, Sabah.

***Lejeuneautriculata*** (Steph.) Mizut. – Mizutani (1966 as *Pycnolejeuneautriculata*, 1970), Lee (2013). Sabah.

***Lejeuneawightii*** Steph. – Mizutani (1966, 1970). Sabah.

***Lepicolearara*** (Steph.) Grolle – Inoue (1967a, 1968b both as *L.loriana*), Kitagawa (1978 as *L.loriana*), Pocock et al. (1984 as *L.loriana*), Kürschner (1990), Cheah (2017). Pahang, Sabah.

***Lepicoleayakusimensis*** (S.Hatt.) S.Hatt. – Inoue (1967a), Kitagawa and Kodama (1973), Akiyama et al. (2001). Pahang, Sabah.

***Lepidolejeuneabidentula*** (Steph.) R.M.Schust – Hoffmann (1935 as *Pycnolejeuneacorticola* and *P.decurvifolia*), Herzog (1950 as P.badiaf.parvistipa, 1952b as *P.corticola* and *P.nicobarica*), Mizutani (1966, 1970 as *P.bidentula*, *P.punctata* and *P.nicobarica*), Inoue (1967a as *P.bidentula*), Kitagawa (1969c as *P.nicobarica*), Kitagawa and Kodama (1974 as *P.bidentula* and *P.nicobarica*), Piippo (1986), Wolseley et al. (1996), Pócs et al. (2020), Pesiu et al. (2021). Penang, Selangor, Pahang, Negeri Sembilan, Terengganu, Johor, Sarawak, Sabah.

***Lepidolejeuneaintegristipula*** (J.B.Jack et Steph.) R.M.Schust. – Mizutani (1970, 1972b both as *Pycnolejeuneaintegristipula*), Kitagawa and Kodama (1974 as *P.integristipula*), Piippo (1986), Cheah (2017). Pahang, Sabah.

***Lepidoziaambigua*** De Not. – De Notaris (1874), Schiffner (1898), Stephani (1909). Sarawak.

***Lepidoziaborneensis*** Steph. – Herzog (1950), Kitagawa and Kodama (1973), Mizutani (1974), Kamimura (1975), Ludwiczuk and Asakawa (2010). Sarawak, Sabah.

***Lepidoziabrotheri*** Steph. – Kürschner (1990). Sabah.

***Lepidoziacladorhiza*** (Reinw., Blume et Nees) Nees – Mitten and Wright (1894), Herzog (1950 as L.cladorhizavar.macgregorii), Kitagawa and Kodama (1973), Mizutani (1974), Kürschner (1990), Akiyama et al. (2001). Sarawak, Sabah.

***Lepidoziaeverettii*** Steph. – Stephani (1909), Herzog (1950), Mizutani (1968). Sarawak.

***Lepidoziafauriana*** Steph. – Mizutani (1974), Ludwiczuk and Asakawa (2010). Sabah.

***Lepidoziaferdinandi-muelleri*** Steph. – Kitagawa and Kodama (1973), Mizutani (1974). Sabah.

***Lepidoziahaskarliana*** (Gottsche, Lindenb. et Nees) Steph. – Mizutani (1974). Sabah.

***Lepidoziaholorhiza*** (Reinw., Blume et Nees) Nees – Mitten and Wright (1894), Inoue (1968b). Pahang, Sabah.

***Lepidoziakinabaluensis*** Mizut. – Mizutani (1974). Sabah.

***Lepidozialacerifolia*** Steph. – Herzog (1950). Sarawak.

***Lepidoziamiqueliana*** Sande Lac. – De Notaris (1874), Schiffner (1898), Herzog (1950). Sarawak.

***Lepidoziareptans*** (L.) Dumort. – Mizutani (1974). Sabah.

***Lepidoziarichardsii*** Herzog – Herzog (1950). Sarawak.

***Lepidoziasquamifolia*** Steph. – Mizutani (1968, 1974). Sarawak, Sabah.

***Lepidoziasubintegra*** Lindenb. – Mitten and Wright (1894), Herzog (1950). Sarawak, Sabah.

***Lepidoziasupradecomposita*** Lindenb. – Kitagawa and Kodama (1973), Mizutani (1974), Akiyama et al. (2001). Sabah.

***Lepidoziatrichodes*** (Reinw., Blume et Nees) Nees – Mitten and Wright (1894), Herzog (1950), Inoue (1967a), Kitagawa (1969c), Kitagawa and Kodama (1973), Mizutani (1974), Kamimura (1975), Pocock et al. (1984), Kürschner (1990). Penang, Pahang, Sarawak, Sabah.

***Leptolejeuneaamphiophthalma*** Zwickel – Mizutani (1966 as *L.picta*), Kitagawa and Kodama (1974 as *L.picta*), Tixier (1974 as *L.picta*), Grolle (1988), Pócs et al. (2020), Pesiu et al. (2021). Kedah, Terengganu, Sarawak, Sabah.

***Leptolejeuneaapiculata*** (Horik.) S.Hatt. – Pócs and Lee (2016). Pahang.

***Leptolejeuneabalansae*** Steph. – Dürhammer and Schäfer-Verwimp (1995). Perak.

***Leptolejeuneaborneensis*** Herzog – Herzog (1942), Mizutani (1966). Sabah.

***Leptolejeuneadentistipula*** Steph. – Herzog (1942). Sarawak.

***Leptolejeuneaepiphylla*** (Mitt.) Steph. – Herzog (1942), Mizutani (1966), Kitagawa (1969c), Kitagawa and Kodama (1974), Tixier (1974), Thiers (1986), Wolseley et al. (1996), Pócs et al. (2020), Pesiu et al. (2021, in press). Kedah, Penang, Selangor, Pahang, Negeri Sembilan, Terengganu, Sabah.

***Leptolejeuneafoliicola*** Steph. – Herzog (1942), Tixier (1971, 1974). Kedah, Pahang, Johor.

***Leptolejeunealigulata*** Herzog – Herzog (1942), Pócs et al. (2020). Pahang, Sabah.

***Leptolejeuneamaculata*** (Mitt.) Schiffn. – Herzog (1942 as L.schiffnerif.latifolia,f.angustifoliaandvar.subintegerrima), Tixier (1974, 1980a both as *L.radiata*), Kitagawa and Kodama (1974 as *L.schiffneri*), Mizutani (1966, 1970 both as *L.schiffneri*, 1975), Dürhammer and Schäfer-Verwimp (1995), Wolseley et al. (1996 as *L.schiffneri*), Pócs et al. (2020), Pesiu et al. (2021, in press). Kedah, Perak, Pahang, Negeri Sembilan, Terengganu, Johor, Sarawak, Sabah.

***Leptolejeuneamassartiana*** Herzog – Tixier (1971). Pahang.

***Leptolejeunearenneri*** Herzog – Shu et al. (2021). Kelantan.

***Leptolejeuneasubacuta*** A.Evans – Herzog (1942), Mizutani (1966, also as *L.elliptica*), Tixier (1971), Kitagawa and Kodama (1974 as *L.elliptica*), Wolseley et al. (1996 as L.ellipticasubsp.subacuta), Pócs et al. (2020 as *L.elliptica*), Pesiu et al. (2021, in press). Perak, Pahang, Negeri Sembilan, Terengganu, Johor, Sabah.

***Leptolejeuneasubdentata*** Herzog – Herzog (1942), Mizutani (1966), Pócs et al. (2020). Johor, Sarawak, Sabah.

***Leptolejeuneasubrotundifolia*** Herzog – Shu et al. (2021). Kelantan, Pahang.

***Leptolejeuneatripuncta*** (Mitt.) Steph. – Herzog (1942 as *L.serrulata*), Inoue (1967a as *L.serrulata*), Kitagawa (1969c as *L.serrulata*), Thiers (1986 as *L.serrulata*), Pócs et al. (2020), Shu et al. (2021). Penang, Selangor, Pahang, Sabah.

***Leptolejeuneavitrea*** (Nees) Schiffn. – Herzog (1942), Mizutani (1966), Kitagawa (1969c), Tixier (1971, 1974, 1980a), Kitagawa and Kodama (1974), Wolseley et al. (1996), Pócs et al. (2020), Pesiu et al. (2021, in press). Kedah, Penang, Perak, Pahang, Negeri Sembilan, Terengganu, Johor, Sabah.

***Lobatiriccardiacoronopus*** (De Not.) Furuki – Herzog (1950 as Riccardialobataandf.gigantea), Furuki (1996 as *L.lobata*). Pahang, Sarawak, Sabah.

***Lophocoleabidentata*** (L.) Dumort. – Piippo (1989 as *Chiloscyphuslatifolius*), Cheah (2017 as *C.coadunatus* and *C.cuspidatus*). Pahang, Sabah.

***Lophocoleakurzii*** Sande Lac. – Kitagawa and Kodama (1974), Piippo (1989 as *Chiloscyphuskurzii*). Sabah.

***Lophocoleamollis*** (Nees) Nees – Inoue (1967a). Pahang.

***Lophocoleamuricata*** (Lehm.) Nees – Kitagawa and Kodama (1974), Piippo (1989 as *Chiloscyphusmuricatus*), Müller and Schäfer-Verwimp (1999), Cheah (2017 as *C.muricatus*). Pahang, Sabah.

***Lophocoleasikkimensis*** (Steph.) Herzog et Grolle – Piippo (1989 as *Chiloscyphussikkimensis*). Sabah.

***Lophocoleasteetziae*** De Not. – De Notaris (1874), Schiffner (1898), Piippo (1989 as *Chiloscyphussteetziae*).

***Lopholejeuneaapplanata*** (Reinw., Blume et Nees) Schiffn. – Verdoorn (1934a), Mizutani (1969), Kitagawa and Kodama (1974), Zhu and Gradstein (2005). Perak, Sabah.

***Lopholejeuneaborneensis*** (Steph.) Verd. – Verdoorn (1934a), Zhu and Gradstein (2005). Sarawak, Sabah.

***Lopholejeuneaceylanica*** Steph. – Mizutani (1985), Zhu and Gradstein (2005), Cheah (2017), Pesiu et al. (in press). Pahang, Terengganu, Sabah.

***Lopholejeuneaeulopha*** (Taylor) Schiffn. – Stephani (1912 as *L.cranstonii*), Verdoorn (1934a), Kitagawa (1969a), Mizutani (1969, 1979b), Kitagawa and Kodama (1974), Zhu and Gradstein (2005), Lee et al. (2013), Cheah (2017 as *L.nicobarica*), Pócs et al. (2020), Pesiu et al. (in press). Penang, Perak, Pahang, Terengganu, Johor, Sarawak, Sabah.

***Lopholejeuneaherzogiana*** Verd. – Verdoorn (1934a), Mizutani (1969, 1985), Kitagawa and Kodama (1974), Tixier (1974), Thiers (1983), Zhu and Gradstein (2005). Kedah, Pahang, Sabah.

***Lopholejeuneahorticola*** Schiffn. – Mizutani (1985). Sabah.

***Lopholejeuneamagna*** Mizut. – Mizutani (1969), Kitagawa and Kodama (1974), Zhu and Gradstein (2005). Sabah.

***Lopholejeuneanigricans*** (Lindenb.) Schiffn. – Mizutani (1969, 1985 as *L.javanica*), Kitagawa and Kodama (1974), Zhu and Gradstein (2005), Lee et al. (2013), Cheah (2017), Pócs et al. (2020), Pesiu et al. (in press). Perak, Pahang, Terengganu, Sarawak, Sabah.

***Lopholejeunearecurvata*** Mizut. – Zhu and Gradstein (2005). Pahang.

***Lopholejeuneasubfusca*** (Nees) Schiffn. – Verdoorn (1934a), Herzog (1950), Mizutani (1966, 1969), Kitagawa (1969a), Tixier (1971, 1980a), Kamimura (1974), Kitagawa and Kodama (1974), Kürschner (1990), Wolseley et al. (1996), Zhu and Gradstein (2005), Lee et al. (2013), Pócs et al. (2020). Perak, Penang, Pahang, Negeri Sembilan, Sarawak, Sabah.

***Lopholejeuneawiltensii*** Steph. – Mizutani (1979a), Akiyama et al. (2001), Zhu and Gradstein (2005). Pahang, Sabah.

***Lopholejeuneazollingeri*** (Steph.) Schiffn. – Verdoorn (1934a), Mizutani (1969, also as *L.nipponica*, 1981b), Kitagawa and Kodama (1974), Zhu and Gradstein (2005), Cheah (2017 as *L.latialata*). Pahang, Sabah.

***Marchantiaacaulis*** Steph. – Tixier (1971), Bischler-Causse (1989). Perak, Pahang, Sarawak, Sabah.

***Marchantiaemarginata*** Reinw., Blume et Nees – Bischler-Causse (1989), Gepp (1914). Selangor, Sarawak, Sabah.

***Marchantiageminata*** Reinw., Blume et Nees – Johnson (1958), Bischler-Causse (1989). Perak, Pahang, Selangor.

***Marchantianovoguineensis*** Bischl. – Bischler-Causse (1989). Sabah.

***Marchantiapolymorpha*** L. – Johnson (1958), Kitagawa and Kodama (1974), Bischler-Causse (1989). Perak, Sabah.

***Marchantiastreimannii*** Bischl. – Bischler-Causse (1989). Sabah.

***Marchantiasubgeminata*** Steph. – Bischler-Causse (1989). Sabah.

***Marsupellaemarginata*** (Ehrh.) Dumort. – Kitagawa (1967), Gradstein and Váňa (1987), Váňa (1991a). Sabah.

***Marsupellastoloniformis*** N.Kitag. – Kitagawa (1967), Váňa (1991a). Sabah.

***Marsupellavermiformis*** (R.M.Schust.) Bakalin et Fedosov. – Bakalin et al. (2021). Sabah.

***Mastigopelmapulvinulatum*** (De Not.) Grolle – De Notaris (1874 as *Mastigobryumpulvinulatum*), Schiffner (1898 as *Bazzaniapulvinulata*), Herzog (1950 as *M.bilobum*). Sarawak.

***Mastigophoradiclados*** (F.Weber) Nees – De Notaris (1874 as Sendtneradicladosvar.borneensis), Verdoorn (1932c), Herzog (1950, 1952b), Johnson (1958), Inoue (1967a, 1968b), Tixier (1971), Kitagawa and Kodama (1973), Kamimura (1975), Kodama (1976), Kürschner (1990), Ludwiczuk and Asakawa (2010), Ng et al. (2017). Kedah, Pahang, Johor, Sarawak, Sabah.

***Mastigophoradiclados*** var. ***borneensis*** (De Not.) Schiffn. – Schiffner (1898). Sarawak.

***Mastigophoradiclados*** var. ***ramentiﬁssa*** Herzog – Herzog (1950). Sarawak.

***Mastigophoradiclados*** var. ***villosa*** Herzog – Herzog (1950). Sarawak.

***Mesoptychiasubcrispa*** (Herzog) L.Söderstr. et Váňa – Cheah (2017 as *Hattoriellasubcrispa*). Pahang.

***Metalejeuneacucullata*** (Reinw., Blume et Nees) Grolle – Herzog (1950 as *Microlejeuneasundaica*), Mizutani (1966, 1970 both as *Lejeuneacucullata*), Hattori and Kamimura (1971), Tixier (1971, 1974, 1980a as *M.cucullata*), Kitagawa and Kodama (1974 as *L.cucullata*), Kürschner (1990 as *L.cucullata*), Wolseley et al. (1996 as *M.cucullata*), Pócs et al. (2020), Pesiu et al. (2021). Kedah, Pahang, Negeri Sembilan, Terengganu, Sarawak, Sabah.

***Metzgeriaciliata*** Raddi – Kuwahara (1965 as *M.decipiens*), Kürschner (1990 as *M.decipiens*), Cheah (2017). Pahang, Sabah.

***Metzgeriafoliicola*** Schiffn. – Kuwahara (1965), Kitagawa and Kodama (1974). Sabah.

***Metzgeriafrancana*** Steph. – Kuwahara (1965 as *Austrometzgeriafrancana*). Sabah.

***Metzgeriafurcata*** (L.) Corda – Kuwahara (1965 as *M.molokaiensis*), Tixier (1971). Pahang, Sabah.

***Metzgeriakinabaluensis*** (Kuwah.) Masuzaki – Kuwahara (1965 as Apometzgeriapubescensvar.kinabaluensis), Kitagawa and Kodama (1974 as A.pubescensvar.kinabaluensis), Kürschner (1990 as A.pubescensvar.kinabaluensis), Akiyama et al. (2001 as A.pubescensvar.kinabaluensis), Masuzaki (2011). Sabah.

***Metzgerialeptoneura*** Spruce – Herzog (1950 as *M.hamata*), Kuwahara (1965 as *M.borneensis*, *M.hamata* and *M.sandei*, 1976 as *M.thomeensis* and *M.papulosa*, 1984 as *M.sharpii*), Johnson (1972 as *M.hamata*), Kitagawa and Kodama (1974 as *M.hamata*), So (2003a). Pahang, Sarawak, Sabah.

***Metzgerialindbergii*** Schiffn. – Kuwahara (1965 as M.conjugatasubsp.japonica and *M.pectinata*), So (2003a), Cheah (2017). Pahang, Sabah.

***Metzgeriascobina*** Mitt. – Mitten (1886), Schiffner (1898), Kitagawa (1969c), Thiers (1983), So (2003a). Penang, Selangor, Sarawak.

***Microlejeuneaconstricta*** (Grolle) Grolle – Mizutani (1973 as *Harpalejeuneaconstricta*), Pócs et al. (2020). Sabah.

***Microlejeuneafilicuspis*** (Steph.) Heinrichs, Schäf.-Verw., Pócs et S.Dong – Mizutani (1970 as *Drepanolejeuneafilicuspis*, 1973 as *Harpalejeuneafilicuspis* and *H.riddleana*), Pócs and Lee (2016), Cheah (2017 as *H.filicuspis*), Pócs et al. (2020). Kelantan, Pahang, Sabah.

***Microlejeuneakinabaluensis*** (Mizut.) Grolle – Mizutani (1973 as *Harpalejeuneakinabaluensis*), Grolle and Reiner-Drehwald (1999). Sabah.

***Microlejeunealunulatiloba*** Horik. – Bischler et al. (1962 as *M.gracillima*), Pócs et al. (2020). Malacca, Sabah.

***Microlejeuneamammillosa*** (Mizut.) Grolle – Mizutani (1973 as *Harpalejeuneamammillosa*), Grolle and Reiner-Drehwald (1999). Sabah.

***Microlejeuneaminutissima*** (Mizut.) Grolle – Mizutani (1973 as *Harpalejeuneaminutissima*), Grolle and Reiner-Drehwald (1999). Sabah.

***Microlejeuneapunctiformis*** (Taylor) Steph. – Kamimura (1974 as *Lejeuneapunctiformis*), Pócs and Lee (2016), Pócs et al. (2020), Pesiu et al. (2021, in press). Kelantan, Terengganu, Sabah.

***Microlejeuneaspinosa*** (Mizut.) Grolle – Mizutani (1973 as *Harpalejeuneaspinosa*), Grolle and Reiner-Drehwald (1999). Sabah.

***Mizutaniariccardioides*** Furuki et Z.Iwats. – Furuki and Iwatsuki (1989), Masuzaki et al. (2010). Pahang, Johor, Sarawak, Sabah.

***Mniolomafuscum*** (Lehm.) R.M.Schust. – Grolle (1965 as *Calypogeiabaldwinii*), Bisang et al. (2003 as *C.fusca*), Cheah (2017). Pahang, Sarawak.

***Mohamediaborneensis*** (Steph.) R.L.Zhu et L.Shu – Mizutani (1972b as *Pycnolejeuneaborneensis*), Piippo (1986 as *Lepidolejeuneaborneensis*), Zhu et al. (2019). Sarawak, Sabah.

***Mohamediabrunnea*** (Mizut.) R.L.Zhu et L.Shu – Mizutani (1970 as *Drepanolejeuneabrunnea*), Pócs and Lee (2016 as *D.brunnea*), Zhu et al. (2019). Pahang, Sabah.

***Myriocoleopsisminutissima*** (Sm.) R.L.Zhu, Y.Yu et Pócs – Pócs et al. (2020). Sabah.

***Nardiaassamica*** (Mitt.) Amakawa – Váňa (1991b). Sabah.

***Neolepidozialongitudinalis*** (Herzog) E.D.Cooper – Herzog (1950 as *Lepidozialongitudinalis*), Engel and Merrill (2004 as *Telaranealongitudinalis*). Sarawak.

***Neolepidoziamamillosa*** (Schiffn.) E.D.Cooper – Grolle (1967 as *Lepidoziamamillosa*), Mizutani (1974b as *L.mamillosa*). Sabah.

***Neolepidoziaophiria*** (Gottsche) E.D.Cooper – Stephani (1909 as *Lepidoziaophiria*), Mizutani (1974b as *L.ophiria*), Pocock et al. (1984 as *L.ophiria*), Engel and Merrill (2004 as *Telaraneaophiria*). Pahang, Johor (“Mt. Ophir” (Gunung Ledang) was mistakenly listed as in Malacca), Sabah.

***Neolepidoziapapulosa*** (Steph.) Fulford et J.Taylor – Kitagawa (1973 as *Lepidoziapapulosa*), Kitagawa and Kodama (1973 as *L.papulosa*). Sarawak, Sabah.

***Neolepidoziawallichiana*** (Gottsche) Fulford et J.Taylor – De Notaris (1874 as *Lepidoziawallichiana*), Mitten and Wright (1894 as *L.wallichiana*), Herzog (1950 as *L.wallichiana*), Kitagawa and Kodama (1973 as *L.wallichiana*), Mizutani (1974 as *L.wallichiana*), Kamimura (1975 as *L.wallichiana*), Pocock et al. (1984 as *L.wallichiana*), Kürschner (1990 as *L.wallichiana*), Wolseley et al. (1996 as *L.wallichiana*), Bisang et al. (2003 as *L.wallichiana*). Penang, Pahang, Negeri Sembilan, Sarawak, Sabah.

***Notoscyphuslutescens*** (Lehm. et Lindenb.) Mitt. – Herzog (1950 as *N.paroicus*), Inoue (1967a), Kitagawa (1969c as *N.paroicus*), Kitagawa and Kodama (1973 as *N.paroicus*). Penang, Pahang, Sarawak, Sabah.

***Nowelliaborneensis*** (De Not.) Schiffn. – De Notaris (1874 as Jungermanniacurvifoliavar.borneensis), Schiffner (1898), Herzog (1950), Grolle (1968), Kitagawa and Kodama (1974), Akiyama et al. (2001), Cheah (2017). Pahang, Sarawak, Sabah.

***Nowelliacurvifolia*** (Dicks.) Mitt. – Grolle (1968), Váňa (1993). Johor, Sabah.

***Nowellialangii*** Pearson – Pearson (1922), Grolle (1968), Kitagawa and Kodama (1974), Váňa (1993). Perak, Johor, Sabah.

***Odontoschismadenudatum*** (Mart.) Dumort. subsp. ***naviculare*** (Steph.) Gradst., S.C.Aranda et Vanderp. – Kitagawa and Kodama (1974 as *O.denudatum*), Gradstein and Váňa (1987 as *O.denudatum*), So (2004 as *O.denudatum*), Gradstein and Ilkiu-Borges (2015). Pahang, Johor, Sabah.

***Odontoschismajishibae*** (Steph.) L.Söderstr. et Váňa – Kitagawa (1964 as *Iwatsukiaexigua*), Kürschner (1990 as *I.exigua*), Váňa (1993 as *I.exigua*), Gradstein and Ilkiu-Borges (2015). Sabah.

***Odontoschismapurpuratum*** Herzog – Herzog (1950, 1952b), So (2004), Gradstein and Ilkiu-Borges (2015). Pahang, Sarawak, Sabah.

***Pallaviciniaambigua*** (Mitt.) Steph. – Kitagawa and Kodama (1974 as *P.fistulosa*), Grolle and Piippo (1986). Sabah.

***Pallaviciniaindica*** Schiffn. – Herzog (1950), Grolle (1969), Kitagawa (1971), Johnson (1972). Penang, Pahang, Sarawak, Sabah.

***Pallavicinialevieri*** Schiffn. – Johnson (1972), Grolle and Piippo (1986). Pahang, Sabah.

***Pallavicinialyellii*** (Hook.) Gray – Grolle and Piippo (1986). Sarawak.

***Pedinophyllumautoicum*** (Steph.) Inoue – Kamimura (1975). Sabah.

***Pictolejeuneamizutanii*** Grolle – Mizutani (1970 as *Cheilolejeuneapicta*), Kitagawa and Kodama (1974 as *C.picta*), Grolle (1977), Thiers (1986), Wolseley et al. (1996), Bi et al. (2019). Negeri Sembilan, Sarawak, Sabah.

***Plagiochilaabietina*** (Nees) Mont. et Nees – Herzog (1950), Inoue (1984). Sarawak, Sabah.

***Plagiochilaarbuscula*** (Lehm. et Lindenb.) Lindenb. – Kitagawa and Kodama (1974), Kamimura (1975), Inoue (1984, 1989), Kürschner (1990). Pahang, Terengganu, Sabah.

***Plagiochilabantamensis*** (Reinw., Blume et Nees) Mont. – De Notaris (1874 as *P.mutabilis*), Herzog (1950 as *P.mutabilis* and *P.richardsii*), Inoue (1967a as *P.lobulata*, 1984, 1989), Kitagawa and Kodama (1974), Wolseley et al. (1996), So and Grolle (2000), So (2001), Pesiu et al. (in press). Perak, Pahang, Negeri Sembilan, Terengganu, Sarawak, Sabah.

***Plagiochilabicornuta*** Steph. – Stephani (1903 as *P.laxissima*), Inoue (1989 as *P.laxissima*). Cheah (2017). Pahang, Sarawak, Sabah.

***Plagiochilablepharophora*** (Nees) Lindenb. – Herzog (1950 as *P.estipulata* and *P.elegantissima*), Inoue (1984, 1989), So and Grolle (2000), So (2001), Cheah (2017). Pahang, Sarawak, Sabah.

***Plagiochilaborneensis*** Steph. – Stephani (1918). Sarawak.

***Plagiochilachauviniana*** Mont. – Herzog (1950 as *P.vanikorensis*). Sarawak.

***Plagiochilaclavatosaccata*** Steph. – Inoue (1984), So and Grolle (2000 as *P.longifolia*), So (2001). Perak, Sabah.

***Plagiochiladenigrata*** Inoue – Inoue (1989), So and Grolle (2000). Sabah.

***Plagiochiladolichoblasta*** – Herzog (1950), So and Grolle (2000). Sarawak.

***Plagiochilafrondescens*** (Nees) Lindenb. – De Notaris (1874), Herzog (1950), Kitagawa and Kodama (1974), Kamimura (1975), Inoue (1967a, 1984, 1989). Pahang, Sarawak, Sabah.

***Plagiochilagymnoclada*** Sande Lac. – Inoue (1984, 1989), Kürschner (1990), So and Grolle (2000), So (2001). Sabah.

***Plagiochilahampeana*** Gottsche – Inoue (1968b, 1969, 1984 all as *P.cameronensis*, 1989), Kürschner (1990). Pahang, Sabah.

***Plagiochilaintegrilobula*** Schiffn. – Inoue (1989). Sabah.

***Plagiochilajavanica*** (Sw.) Nees et Mont – Inoue (1989, also as *P. inﬁrma*), Kamimura (1975), Cheah (2017). Pahang, Sabah.

***Plagiochilajunghuhniana*** Sande Lac. – Kitagawa and Kodama (1974), Kamimura (1975), Cheah (2017). Pahang, Sabah.

***Plagiochilakorthalsiana*** Molk. – Kitagawa and Kodama (1974 as *P.accedens*), Inoue (1989), So (2000). Sabah.

***Plagiochilakuhliana*** Sande Lac. – Stephani (1903, 1906). Sarawak.

***Plagiochilakurzii*** Steph. – Kitagawa and Kodama (1974 as *P.sambusana*), Inoue (1984), Wolseley et al. (1996), So and Grolle (2000), So (2001). Negeri Sembilan, Sarawak, Sabah.

***Plagiochilamassalongoana*** Schiffn. – Kitagawa and Kodama (1974), Inoue (1984). Pahang, Sabah.

***Plagiochilanitens*** Inoue – Inoue (1989), So and Grolle (2000), So (2001). Sabah.

***Plagiochilanobilis*** Gottsche – Inoue (1989). Sabah.

***Plagiochilaobtusa*** Lindenb. – Inoue (1968b). Pahang.

***Plagiochilapeculiaris*** Schiffn. – Inoue (1984, 1989), Kürschner (1990), Renner (2018). Pahang, Sarawak, Sabah.

***Plagiochilaperserrata*** Herzog – Inoue (1975, 1984 both as *P.hottae*), So and Grolle (2000), So (2001). Sabah.

***Plagiochilaphilippinensis*** Steph. – Inoue (1989). Sabah.

***Plagiochilapropinqua*** Sande Lac. – De Notaris (1874), Schiffner (1898), Stephani (1903), Herzog (1950), Inoue (1984, 1989). Perak, Sarawak, Sabah.

***Plagiochilarenitens*** (Nees) Lindenb. – Inoue (1967a, 1984, 1989), Kürschner (1990). Pahang, Sabah.

***Plagiochilasandei*** Dozy – De Notaris (1874), Schiffner (1898), Herzog (1950 as P.sandeif.remotidens), Kitagawa and Kodama (1974), Kamimura (1975), Inoue (1984, 1989), Müller and Schäfer-Verwimp (1999), Cheah (2017), Renner (2018). Pahang, Sarawak, Sabah.

***Plagiochilasciophila*** Nees – Inoue (1984, 1989), So (2001), Cheah (2017). Pahang, Sarawak, Sabah.

***Plagiochilaspathulifolia*** Mitt. – Kitagawa and Kodama (1974), Inoue (1984, 1989). Sarawak, Sabah.

***Plagiochilasumatrana*** Schiffn. – Inoue (1989). Sabah.

***Plagiochilateysmannii*** Sande Lac. – Kitagawa and Kodama (1974), Inoue (1984, 1989), Grolle and So (1999 as *P.fraseri*). Perak, Sabah.

***Plagiochilatrapezoidea*** Lindenb. – Inoue (1989). Sabah.

***Plagiochilaungarangana*** Sande Lac. – Stephani (1917 as *P.tridens*), Inoue (1975 as *P.tsutomui*, 1984), So and Grolle (2000). Perak, Sabah.

***Pleuroziaacinosa*** (Mitt.) Trevis. – De Notaris (1874 as *Physiotiummyriangium*), Schiffner (1898), Inoue (1967a), Kitagawa (1979b), Thiers (1993). Selangor, Pahang, Sarawak, Sabah.

***Pleuroziaarticulata*** (Lindb.) Lindb. et Lackström – Kürschner (1990). Sabah.

***Pleuroziaconchifolia*** (Hook. et Arn.) Austin – Herzog (1950), Kitagawa and Kodama (1974), Kürschner (1990), Thiers (1993), Akiyama et al. (2001). Sarawak, Sabah.

***Pleuroziagigantea*** (F.Weber) Lindb. – De Notaris (1874 as P.giganteavar.borneensis), Herzog (1950 as *Eopleuroziasimplicissima*), Inoue (1967a, 1968b), Kitagawa and Kodama (1974 also as *E.simplicissima*), Tixier (1974), Kürschner (1990), Thiers (1993), Akiyama et al. (2001), Ludwiczuk and Asakawa (2010). Kedah, Pahang, Malacca, Johor, Sarawak, Sabah.

***Pleuroziajohannis-winkleri*** Herzog – Thiers (1993), Akiyama et al. (2001). Sabah.

***Pleuroziasubinflata*** (Austin) Austin – Kamada et al. (2020). Sabah.

***Plicanthushirtellus*** (F.Weber) R.M.Schust. – Herzog (1950 as *Chandonanthushirtellus*), Kitagawa (1970 as *C.hirtellus*), Kitagawa and Kodama (1973 as *C.hirtellus*), Kürschner (1990 as *C.hirtellus*), Akiyama et al. (2001 as *C.hirtellus*), Ludwiczuk and Asakawa (2010 as *C.hirtellus*), Cheah (2017 as *C.hirtellus*). Pahang, Sarawak, Sabah.

***Podomitriummalaccense*** (Steph.) Campb. – Kitagawa (1969c), Kitagawa and Kodama (1974), Grolle and Piippo (1986), Campbell (1915), Wolseley et al. (1996). Penang, Negeri Sembilan, Johor, Sarawak, Sabah.

***Porellaacutifolia*** (Lehm. et Lindenb.) Trevis. – Kitagawa and Kodama (1974 as P.acutifoliavar.elbertii), Hattori (1967 as *P.johannis-winkleri*, 1969 as P.acutifoliavar.johannis-winkleri). Sarawak, Sabah.

***Porellacaespitans*** (Steph.) S.Hatt. subsp. ***latior*** (S.Hatt.) S.Hatt. – Hattori (1969 as P.acutifoliasubsp.latior). Pahang.

***Porellajaponica*** (Sande Lac.) Mitt. – Hattori (1967). Sabah.

***Porellajavanica*** (Gottsche) Inoue – Inoue (1967a). Pahang.

***Pseudolepicoleaandoi*** (R.M.Schust.) Inoue – Hattori and Mizutani (1968 as P.trolliisubsp.andoi). Sabah.

***Pseudolepicoleatrollii*** (Herzog) Grolle et Ando – Hattori (1966). Sabah.

***Pseudomarsupidiumborneensis*** (Grolle) Váňa, L.Söderstr., A.Hagborg et von Konrat – Grolle (1972 as *Adelanthusborneensis*), Kitagawa and Kodama (1974 as *A.borneensis*). Sabah.

***Psilocladaclandestina*** Mitt. – Herzog (1950), Kitagawa and Kodama (1973), Mizutani (1974), Pocock et al. (1984), Kürschner (1990), Akiyama et al. (2001), Cheah (2017). Pahang, Sarawak, Sabah.

***Ptychanthusstriatus*** (Lehm. et Lindenb.) Nees – Verdoorn (1934a), Herzog (1950), Mizutani (1969), Tixier (1971), Kamimura (1974, 1975), Kitagawa and Kodama (1974). Pahang, Sarawak, Sabah.

***Pycnolejeuneacavistipula*** (Steph.) Mizut. – Mizutani (1970 as *P.molaris*), Kitagawa and Kodama (1974), Kürschner (1990). Sabah.

***Pycnolejeuneacontigua*** (Nees) Grolle – Mizutani (1970 as *P.bancana*), Kürschner (1990 as *P.bancana*), He (1999). Sabah.

***Pycnolejeuneagrandiocellata*** Steph. – Kürschner (1990), Wolseley et al. (1996), He (1999), Schäfer-Verwimp (2006). Pahang, Negeri Sembilan, Sarawak.

***Pycnolejeuneagrossiloba*** Steph. – Hoffmann (1935). Johor.

***Pycnolejeuneasphaeroides*** (Sande Lac.) J.B.Jack et Steph. – Kodama (1976 as *P.kinabaluensis*), He (1999), Schäfer-Verwimp (2006). Pahang, Sabah.

***Radulaacuminata*** Steph. – Yamada (1973, 1979, 1989), Wolseley et al. (1996), Pesiu et al. (2021, in press). Selangor, Negeri Sembilan, Terengganu, Sabah.

***Radulaamentulosa*** Mitt. – Herzog (1950), Yamada (1979, 1989), Cheah (2017). Pahang, Sarawak, Sabah.

***Radulaamoena*** Herzog – Yamada (1979), Yamada and Piippo (1989). Sarawak, Sabah.

***Radulaanceps*** Sande Lac. – De Notaris (1874), Schiffner (1898), Herzog (1950 as *R.laciniata*), Castle (1961), Inoue and Miller (1965), Yamada (1973, also as *R.apiculata*, 1979, 1989), Kitagawa and Kodama (1974), Wolseley et al. (1996), Cheah (2017 as *R.apiculata*). Perak, Pahang, Negeri Sembilan, Sarawak, Sabah.

***Radulaassamica*** Steph. – Pócs et al. (2014), Pesiu et al. (2021, in press). Selangor, Pahang, Terengganu.

***Radulaborneensis*** Steph. – Inoue (1967a), Castle (1936), Yamada (1979, 1989). Pahang, Sarawak, Sabah.

***Radulacaduca*** K.Yamada – Cheah (2017). Pahang.

***Radulacampanigera*** Mont. – Castle (1936), Yamada (1973, 1979, 1989). Perak, Sabah.

***Radulacavifolia*** Gottsche, Lindenb. et Nees – Yamada (1973, 1979, 1989). Sabah.

***Radulaceylanica*** K.Yamada – Yamada (1979). Pahang.

***Raduladensifolia*** Castle – Yamada (1973, 1979, 1989). Sabah.

***Radulafalcata*** Steph. – Cheah (2017). Pahang.

***Radulaformosa*** (Spreng.) Nees – Inoue (1967a as *R.novae-guineae*), Yamada (1973 as *R.novae-guineae*, 1979, 1989), Kitagawa and Kodama (1974), Tixier (1974). Kedah, Pahang, Sabah.

***Radulagedena*** Gottsche – Yamada (1973, 1979, 1989). Sabah.

***Radulagrandilobula*** Promma et Chantanaorr. – Sarimi et al. (2021), Zhang et al. (2021), Pesiu et al. (in press). Terengganu, Sarawak.

***Radulaiwatsukii*** K.Yamada – Yamada (1979, 1989), Yamada and Piippo (1989). Sabah.

***Radulajavanica*** Gottsche – De Notaris (1874), Schiffner (1898), Herzog (1950), Yamada (1973, 1979 also as *R.yangii*, 1989), Kitagawa and Kodama (1974), Wolseley et al. (1996), Pesiu et al. (in press). Selangor, Pahang, Negeri Sembilan, Terengganu, Sarawak, Sabah.

***Radulakinabaluensis*** K.Yamada – Yamada (1979, 1989). Sabah.

***Radulalacerata*** Steph. – Castle (1961), Yamada (1979, 1989), Wolseley et al. (1996). Negeri Sembilan, Sarawak, Sabah.

***Radulalingulata*** Gottsche – Cheah (2017). Pahang.

***Radulamadagascariensis*** Gottsche – Yamada (1979, 1989). Sabah.

***Radulamizutanii*** K.Yamada – Yamada (1973, 1979, 1989), Yamada and Piippo (1989). Sabah.

***Radulamorobeana*** K.Yamada et Piippo – Kürschner (1990). Sabah.

***Radulamultiﬂora*** Schiffn. – Yamada (1979, 1989). Sabah.

***Radulanymannii*** Steph. – Tixier (1971, 1980a), Yamada (1973, 1979, 1989), Kitagawa and Kodama (1974), Pesiu et al. (2021, in press). Pahang, Terengganu, Sabah.

***Radulaobscura*** Mitt. – Yamada (1973, 1979, 1989). Sabah.

***Radulaphilippinensis*** K.Yamada – Yamada (1979). Selangor.

***Radulaprotensa*** Lindenb. – Herzog (1952b), Wolseley et al. (1996). Negeri Sembilan, Sarawak.

***Radularetroﬂexa*** Taylor – Inoue (1968b as *R.miqueliana*), Tixier (1974 as *R.miqueliana*). Yamada (1979, also as R.retroﬂexavar.fauciloba, 1989). Kedah, Selangor, Pahang, Terengganu, Sabah.

***Radulasumatrana*** Steph. – Kamimura (1975), Yamada (1979, 1989). Sarawak, Sabah.

***Radulatabularis*** Steph. – Yamada (1973 as *R.indica*, 1979, 1989), Cheah (2017). Pahang, Sabah.

***Radulatjibodensis*** K.I.Goebel – Kitagawa (1969c), Yamada (1973, 1979, 1989), Kamimura (1975), Kitagawa and Kodama (1974), Pócs et al. (2020), Pesiu et al. (2021, in press). Penang, Pahang, Terengganu, Sabah.

***Radulaventricosa*** Steph. – Yamada (1973, 1979, 1989 all as *R.subpallens*), Kitagawa and Kodama (1974 as *R.subpallens*). Sarawak, Sabah.

***Radulavrieseana*** Sande Lac. – Kitagawa and Kodama (1974), Yamada (1979, 1989), Wolseley et al. (1996). Negeri Sembilan, Sarawak, Sabah.

***Rebouliahemisphaerica*** (L.) Raddi subsp. ***australis*** R.M.Schust. – Gepp (1914 as R.hemisphaericavar.javanica). Sabah.

***Riccardiaalbomarginata*** (Steph.) Schiffn. – Furuki (1995), Cheah (2017). Pahang, Sabah.

***Riccardiaaspera*** (Steph.) Grolle – Furuki (1998). Sabah.

***Riccardiabaumannii*** Hürl. – Furuki (1994). Sabah.

***Riccardiacrassa*** (Schwägr.) C.Massal. – Herzog (1950 as *R.scabra*), Johnson (1958 as *R.scabra*), Kitagawa and Kodama (1974 as *R.scabra*). Pahang, Sarawak, Sabah.

***Riccardiacrassiretis*** Schiffn. – Furuki (2001). Pahang, Sabah.

***Riccardiacrenulata*** Schiffn. – Furuki (2001). Sabah.

***Riccardiadeguchii*** Furuki et K.T.Yong – Furuki et al. (2013). Pahang.

***Riccardiaelata*** (Steph.) Schiffn. – Herzog (1950 also as *R.ridleyi*), Cheah (2017). Pahang, Sarawak.

***Riccardiafruticosa*** (Steph.) Furuki – Furuki (1998), Cheah (2017). Pahang, Sabah.

***Riccardiagrollei*** Furuki – Furuki (1999), Cheah (2017). Pahang, Sabah.

***Riccardiagrossitexta*** (Steph.) Furuki – Furuki (1994). Johor, Sarawak, Sabah.

***Riccardiahattorii*** Furuki – Furuki (1994). Sabah.

***Riccardiaheteroclada*** Schiffn. – Herzog (1950), Furuki (1998), Cheah (2017). Pahang, Sarawak.

***Riccardiainconspicua*** (Steph.) Reeb et Bardat – Furuki (1994 as *R.tenuicostata*). Johor, Sarawak, Sabah.

***Riccardiajackii*** Schiffn. – Johnson (1958). Pahang.

***Riccardianobilis*** (Steph.) Schiffn. – Stephani (1893, 1900), Schiffner (1898). Sarawak.

***Riccardiaparvula*** Schiffn. – Johnson (1958, 1972), Furuki (2001 as *Aneurasubcrenulata*). Perak, Pahang, Sarawak, Sabah.

***Riccardiapindensis*** Hewson – Furuki (1995). Sabah.

***Riccardiaplaniflora*** (Steph.) S.Hatt. var. ***aequatorialis*** Furuki – Furuki (1997), Cheah (2017). Pahang, Sabah.

***Riccardiasumatrana*** Schiffn. – Herzog (1950). Sarawak.

***Ricciafluitans*** L. – Gepp (1914). Sabah.

***Ricciocarposnatans*** (L.) Corda – Gepp (1914). Sabah.

***Saccogynidiumgoebelii*** (Herzog) Grolle – Inoue (1967a, 1968b). Pahang.

***Saccogynidiummuricellum*** (De Not.) Grolle – De Notaris (1874 as *Chiloscyphusmuricellus*), Schiffner (1898), Mitten and Wright (1894 as *Saccogynamuricella*), Schiffner (1898 as *C.muricellus*), Kitagawa and Kodama (1974), Piippo (1989). Sarawak, Sabah.

***Saccogynidiumrigidulum*** (Nees) Grolle – Herzog (1950 as *Saccogynarigidula*), Grolle (1960, 1963 both as *Saccgynidiumjugatum*), Inoue (1967a). Perak, Pahang, Johor, Sarawak, Sabah.

***Sandeothallusradiculosus*** (Schiffn.) R.M.Schust. – Campbell and Williams (1914), Pocock et al. (1984), Crandall-Stotler and Stotler (2007). Perak, Pahang, Sabah.

***Scapaniajavanica*** Gottsche – Herzog (1950 as S.javanicaf.amplifolia), Schäfer-Verwimp (2009), Ludwiczuk and Asakawa (2010). Pahang, Sarawak, Sabah.

***Scapanialepida*** Mitt. – Mitten and Wright (1894), Hattori (1964), Kitagawa and Kodama (1973), Akiyama et al. (2001). Sabah.

***Scapaniaornithopodioides*** (With.) Waddell. – Kodama and Narita (1974). Sabah.

***Schiffneriahyalina*** Steph. – Kitagawa (1973), Kitagawa and Kodama (1974), Grolle and Piippo (1984). Pahang, Sabah.

***Schiffneriolejeuneacumingiana*** (Mont.) Gradst. – Lee et al. (2013). Perak.

***Schiffneriolejeuneanymannii*** (Steph.) Gradst. et Terken. – Stephani (1912 as *Ptychocoleuslongispicus*), Verdoorn (1934a as *P.longispicus*), Gradstein and Terken (1981). Sarawak.

***Schiffneriolejeuneaomphalanthoides*** Verd. – Gradstein (2015). Sabah.

***Schiffneriolejeuneapulopenangensis*** (Gottsche) Gradst. – Gottsche et al. (1845 as *Phragmicomapulopenangensis*), Stephani (1890 as *P.pulopenangensis*, Phragmicomatumida as *Ptychocoleuscranstonii* and *P.tridens*), Schiffner (1898 as *Acrolejeuneapulopenangensis*), Verdoorn (1934a as *Ptychocoleuspulopenangensis*), Herzog (1950 as *Ptychocoleuspulopenangensis*), Kitagawa (1969a as *P.pulopenangensis*), Mizutani (1969 as *Ptychocoleuspulopenangensis*), Thiers and Gradstein (1989), Haerida et al. (2010), Lee et al. (2013). Penang, Pahang, Sarawak, Sabah.

***Schiffneriolejeuneatumida*** (Nees) Gradst. – Nees (1838 as *Ptychanthustumidus*), Stephani (1890 as *Phragmicomatumida*, 1912 as *Ptychocoleussarawakensis* and *P.squarrosifolius*), Schiffner (1898 as *Acrolejeuneatumida*), Verdoorn (1933 as *Phragmicomatumida*, 1934a as, *Ptychocoleussarawakensis*), Inoue (1967a as *Ptychocoleussarawakensis*), Mizutani (1969 as *Ptychocoleussarawakensis* and *P.haskarlianus*), Kitagawa (1971 as *Ptychocoleussarawakensis*), Gradstein and Terken (1981), Lee et al. (2013), Gradstein (2015), Pócs et al. (2020). Penang, Perak, Pahang, Sarawak, Sabah.

***Schistochilaacuminata*** Steph. – Herzog (1950), Kitagawa and Kodama (1973 as *S.wrayana*), So (2003b), Ng et al. (2016). Perak, Sarawak, Sabah.

***Schistochilaaligera*** (Nees et Blume) J.B.Jack et Steph. – De Notaris (1874 as *Gottscheaaligera*, *G.aligeriformis* and *G.philippinensis*), Schiffner (1898 as *G.gaudichaudii* and *S.aligeriformis*), Herzog (1950 also as *S.philippinensis*), Kitagawa (1969c as *S.philippinensis*, 1973), Kitagawa and Kodama (1973, also as *S.philippinensis*), So (2003b). Penang, Selangor, Pahang, Johor, Sarawak, Sabah.

***Schistochilabeccariana*** (De Not.) Trevis. – De Notaris (1874 as *Gottscheabeccariana*), Schiffner (1898), So (2003b). Sarawak, Sabah.

***Schistochilablumei*** (Nees) Trevis. – Gepp (1914 as *S.wallisii*), Inoue (1967a), Kitagawa and Kodama (1973), So (2003b). Perak, Kelantan, Pahang, Sarawak, Sabah.

***Schistochiladoriae*** (De Not) Trevis. – De Notaris (1874 as *Gottscheadoriae*), Schiffner (1898), So (2003b). Pahang, Sarawak, Sabah.

***Schistochilareinwardtii*** (Nees) Schiffn. – So (2003b), Cheah (2017). Pahang, Sabah.

***Schistochilarubriseta*** Steph. – Kürschner (1990). Sabah.

***Schistochilasciurea*** (Nees) Schiffn. – De Notaris (1874 as *Gottscheasciurea*), Schiffner (1898), Herzog (1950), Inoue (1967a, 1968b), Kitagawa and Kodama (1973), Kürschner (1990), So (2003b). Perak, Pahang, Sarawak, Sabah.

***Soellaspinistipula*** (Mizut.) R.L.Zhu et L.Shu – Mizutani (1970 as *Pycnolejeuneaspinistipula*), Bi et al. (2019 as *Leptolejeuneaspinistipula*), Shu et al. (2021). Sarawak, Sabah.

***Solenostomaappressifolium*** (Mitt.) Váňa et D.G.Long – Amakawa (1969b as *Jungermanniakinabalensis* and *J.decolyana*), Váňa (1991b as *J.appressifolia*). Sabah.

***Solenostomaariadne*** (Taylor) Váňa et D.G.Long – Inoue (1967a as *Jungermanniaariadne*), Amakawa (1968 as *J.ariadne*), Kitagawa and Kodama (1973 as *J.ariadne*), Váňa (1991b, 1972 both as *J.ariadne*). Penang, Perak, Pahang, Negeri Sembilan, Sabah.

***Solenostomaborneense*** (Amakawa) Váňa, Hentschel et Heinrichs – Amakawa (1970 as *Jungermanniaborneensis*), Váňa (1991b as *J.borneensis*). Sabah.

***Solenostomacomatum*** (Nees) C.Gao – Inoue (1967a as *Jungermanniacomata*). Pahang.

***Solenostomahaskarlianum*** (Nees) Váňa et D.G.Long – Váňa (1972, 1975, 1991b all as *J.haskarliana*). Perak, Pahang, Sarawak, Sabah.

***Solenostomajavanicum*** (Schiffn.) Steph. – Váňa (1991b as *Jungermanniaherzogiana*). Sabah.

***Solenostomapseudocyclops*** (Inoue) Váňa et D.G.Long – Amakawa (1969b as *Jungermanniapseudocyclops*). Sabah.

***Solenostomariclefii*** Váňa et D.G.Long – Amakawa (1969b as *Jungermanniagrollei*), Váňa (1991b as *J.grollei*). Sabah.

***Solenostomasphaerocarpum*** (Hook.) Steph. – Gradstein and Váňa (1987 as *Jungermanniasphaerocarpa*), Váňa (1991b as *J.sphaerocarpa*). Sabah.

***Solenostomastephanii*** (Schiffn.) Steph. – Amakawa (1969b as *Jungermanniastephanii* and *J.mizutanii*), Váňa (1991b as *J.stephanii*). Sabah.

***Solenostomastrictum*** (Schiffn.) Váňa, Hentschel et Heinrichs – Amakawa (1969b as *Jungermanniastricta*), Váňa (1991b as *J.stricta*). Sabah.

***Solenostomatetragonum*** (Lindenb.) Váňa et D.G.Long – Herzog (1950 as *Plectocoleaparabolica*), Váňa (1991b as *Jungermanniatetragona*). Sarawak, Sabah.

***Solenostomatruncatum*** (Nees) Váňa et D.G.Long – Stephani (1901 as *Jungermanniapolyrhiza*), Horikawa (1943 as *Aploziapolyrhiza*), Herzog (1950 as *Plectocoleatruncata*), Amakawa (1972 as *J.truncata*), Váňa (1991 as *J.truncata*). Penang, Sarawak, Sabah.

***Solenostomatuberculiferum*** (Herzog) Váňa, Hentschel et Heinrichs – Váňa (1991b as *Jungermanniatuberculifera*). Sabah.

***Solenostomavirgatum*** (Mitt.) Váňa et D.G.Long – Váňa (1991b as *Jungermanniavirgata*). Sabah.

***Southbyaorganensis*** Herzog – Kodama and Narita (1974 as *S.grollei*). Sabah.

***Sphenolobopsispearsonii*** (Spruce) R.M.Schust. – Kitagawa (1970 as *Cephaloziapearsonii*), Kodama and Narita (1974), Kürschner (1990 as *S.kitagawae*), Váňa (1991c). Sabah.

***Sphenolobusminutus*** (Schreb.) Berggr. – Kitagawa (1970 as *Anastrophyllumminutum*), Kitagawa and Kodama (1973 as *A.minutum*), Váňa (1991c as *A.minutum*). Sabah.

***Spruceanthusplaniusculus*** (Mitt.) X.Q.Shi, Gradst. et R.L.Zhu – Verdoorn (1934a as *Archilejeuneamariana* and *Spruceanthusmarianus*), Mizutani (1969 as *S.marianus*), Kitagawa and Kodama (1974 as *S.marianus*), Lee et al. (2013 as *A.planiuscula*). Penang, Sarawak, Sabah.

***Spruceanthuspolymorphus*** (Sande Lac.) Verd. – Verdoorn (1934a), Mizutani (1969), Kitagawa and Kodama (1974), Wolseley et al. (1996), Lee et al. (2013). Perak, Pahang, Negeri Sembilan, Sabah.

***Spruceanthussemirepandus*** (Nees) Verd. – Verdoorn (1934a), Sun et al. (2018). Kedah, Sabah.

***Spruceanthussulcatus*** (Nees) Gradst. – Verdoorn (1934a as *Ptychanthussulcatus*), Mizutani (1969 as *P.sulcatus*). Sabah.

***Stictolejeuneabalfourii*** (Mitt.) E.W.Jones – Herzog (1950 as *S.richardsii*), Mizutani (1969 as *S.richardsii*), Gradstein (1985), Thiers and Gradstein (1989). Sarawak, Sabah.

***Symphyogynopsisgottscheana*** (Mont. et Nees) Grolle – Grolle and Piippo (1986 as *S.filicum*), Cheah (2017). Perak, Pahang.

***Syzygiellaautumnalis*** (DC.) K.Feldberg, Váňa, Hentschel et Heinrichs – Akiyama et al. (2001 as *Jamesoniellaautumnalis*). Sabah.

***Syzygiellacontracta*** (Reinw., Blume et Nees) Gradst. et G.E.Lee – Kitagawa (1970 as *Cuspidatulacontracta*), Váňa (1991c as *Jamesoniellacontracta*). Sabah.

***Syzygiellaflaccida*** (Steph.) Gradst. et G.E.Lee – Grolle (1971 as *Anomacaulisflaccidus*), Váňa (1991c as *A.flaccidus*). Sabah.

***Syzygiellaflexicaulis*** (Nees) Gradst. et G.E.Lee – Inoue (1967a as *Jamesoniella ﬂexicaulis*), Amakawa (1969a as J.flexicaulisvar.mizutanii), Grolle (1971 as *J. ﬂexicaulis*), Kürschner (1990 as *J.flexicaulis*), Váňa (1991c as *J. ﬂexicaulis*). Pahang, Sabah.

***Syzygiellanipponica*** (S.Hatt.) K.Feldberg, Váňa, Hentschel et Heinrichs – Amakawa (1969a as *Jamesoniellaperverrucosa*), Grolle (1971 as *J.nipponica*), Kürschner (1990 as *J.nipponica*), Váňa (1991c as *J.nipponica*). Sabah.

***Syzygiellaovalifolia*** Inoue – Váňa (1991c). Sabah.

***Syzygiellasecurifolia*** (Nees) Inoue – Inoue (1966, 1967a, 1968b all as *S.variegata*), Kitagawa and Kodama (1974 as *S.variegata*). Pahang, Sabah.

***Syzygiellasonderi*** (Gottsche) K.Feldberg, Váňa, Hentschel et Heinrichs – Amakawa (1969b as *Jungermanniaiwatsukii*), Kitagawa and Kodama (1973 as *J.iwatsukii*), Váňa (1991c as *Cryptochilagrandiﬂora*). Sabah.

***Syzygiellasubintegerrima*** (Reinw., Blume et Nees) Spruce – Inoue (1966, 1967a, 1968a), Kitagawa and Kodama (1974), Váňa (1991c). Pahang, Sabah.

***Telaraneamajor*** (Herzog) J.J.Engel et G.L.Merr – Herzog (1950 as *Arachniopsismajor*), Kitagawa and Kodama (1973 as *A.major*), Mizutani (1974b as *A.major*), Manuel (1981 as *A.major*), Engel and Merrill (2004). Pahang, Sarawak, Sabah.

***Telaraneamonocera*** (R.M.Schust. et Grolle) J.J.Engel et G.L.Merr. – Piippo (1985 as *Arachniopsismonocera*), Engel and Merrill (2004). Johor [“Mt. Ophir” (Gunung Ledang)].

***Telaraneapapulosa*** (Steph.) J.J.Engel et G.L.Merr. – Cheah (2017). Pahang.

***Temnomasetigerum*** (Lindenb.) R.M.Schust. – Kitagawa and Kodama (1973). Sabah.

***Tetralophoziafiliformis*** (Steph.) Urmi – Kitagawa (1973 as *Chandonanthusfiliformis*), Kitagawa and Kodama (1973 as *C. ﬁliformis*), Kodama and Narita (1974 as *C. ﬁliformis*). Sabah.

***Tetralophoziapilifera*** (Steph.) R.M.Schust. – Kürschner (1990 as *Chandonanthuspilifer* [‘*piliferus*’]). Sabah.

***Thiersianthussilamensis*** R.L.Zhu et L.Shu – Zhu et al. (2017). Sabah.

***Thysananthusaculeatus*** Herzog – Verdoorn (1934a as *T.richardsianus*), Herzog (1950 as *T.richardsianus*), Inoue (1967a, 1968b), Mizutani (1969), Kamimura (1974), Thiers (1985), Sukkharak (2015). Pahang, Sarawak, Sabah.

***Thysananthusciliaris*** (Sande Lac.) Sukkharak – Sukkharak (2015). Perak.

***Thysananthuscomosus*** Lindenb. – Lehmann (1844), Stephani (1890), Schiffner (1898), Mizutani (1977), Sukkharak et al. (2011), Sukkharak (2015). Kedah, Penang, Perak, Selangor, Johor, Sarawak, Sabah.

***Thysananthusconvolutus*** Lindenb. – Stephani (1912 as *T.laceratus*), Verdoorn (1934a), Herzog (1950), Kamimura (1974), Kitagawa and Kodama (1974), Sukkharak et al. (2011), Sukkharak (2015). Penang, Selangor, Kelantan, Pahang, Johor, Sarawak, Sabah.

***Thysananthusconvolutus*** var. ***laceratus*** (Steph.) Sukkharak – Sukkharak (2015). Sabah.

***Thysananthusfruticosus*** (Lindenb. et Gottsche) Schiffn. – Verdoorn (1934a), Herzog (1950 as T.fruticosusf.pendulus), Mizutani (1969, 1987), Kitagawa and Kodama (1974), Kürschner (1990), Wolseley et al. (1996), Sukkharak (2015). Pahang, Selangor, Negeri Sembilan, Johor, Sarawak, Sabah.

***Thysananthusgottschei*** (J.B.Jack et Steph.) Steph. – Stephani (1912 as *T.borneensis*), Verdoorn (1934a), Kamimura (1974), Kitagawa and Kodama (1974), Thiers (1985), Sukkharak (2015). Penang, Pahang, Sarawak, Sabah.

***Thysananthusgottschei*** var. ***continuus*** Sukkharak – Sukkharak (2015). Sarawak.

***Thysananthusgradsteinii*** (Sukkharak) Sukkharak et Gradst. – Sukkharak and Gradstein (2014 as *Mastigolejeuneagradsteinii*). Kelantan, Pahang.

***Thysananthushumilis*** (Gottsche) Sukkharak et Gradst. – Mizutani (1969 as *Mastigolejeuneahumilis*, 1986b as *M.recurvifolia*), Kitagawa (1971 as *M.humilis*), Kamimura (1974 as *M.humilis*), Sukkharak and Gradstein (2014 as *M.humilis*), Cheah (2017 as *M.humilis*). Penang, Pahang, Sarawak, Sabah.

***Thysananthusindicus*** (Steph.) Sukkharak et Gradst. – Sukkharak and Gradstein (2014 as *M.indica*). Kedah.

***Thysananthusligulatus*** (Lehm. et Lindenb.) Sukkharak et Gradst. – Stephani (1890 as *Phragmicomaligulata*), Schiffner (1898 as *Mastigolejeunealigulata*), Verdoorn (1934a as *M.ligulata*), Mizutani (1969 as *M.ligulata*), Kitagawa and Kodama (1974 as *M.ligulata*), Thiers and Gradstein (1989 as *M.ligulata*), Kürschner (1990), Gradstein et al. (2002 as *M.ligulata*), Lee et al. (2013 as *M.ligulata*), Sukkharak and Gradstein (2014 as *M.ligulata*). Penang, Pahang, Sarawak, Sabah.

***Thysananthusreconditus*** (Steph.) Sukkharak et Gradst. – Mizutani (1969 as *Mastigolejeunearecondita*), Sukkharak and Gradstein (2014 as *M.recondita*), Sun et al. (2018). Pahang, Sarawak, Sabah.

***Thysananthusrepletus*** (Taylor) Sukkharak et Gradst. – Mizutani (1969 as *Mastigolejeuneaatypos*, 1986b as *M.repleta*), Sukkharak and Gradstein (2014 as *M.repleta*). Kedah, Selangor, Sabah.

***Thysananthusretusus*** (Reinw., Blume et Nees) B.M.Thiers et Gradst. – Mizutani (1969, 1987 both as *T.planus*), Tixier (1971). Pahang, Sabah.

***Thysananthusspathulistipus*** (Reinw., Blume et Nees) Lindenb. – Verdoorn (1934a), Herzog (1950 as T.spathulistipusf.borneensis), Inoue (1967a, 1968b), Tixier (1971, 1974), Kamimura (1974), Kitagawa and Kodama (1974), Kodama (1976), Thiers (1985), Kürschner (1990), Sukkharak (2015), Cheah (2017 as *T.minor*). Kedah, Penang, Perak, Kelantan, Pahang, Negeri Sembilan, Malacca, Johor, Sarawak, Sabah.

***Thysananthustruncatus*** (Mizut.) Sukkharak et Gradst. – Mizutani (1986b as *Mastigolejeuneatruncata*), Sukkharak and Gradstein (2014 as *M.truncata*). Sabah.

***Thysananthusvirens*** Ångstr. – Mizutani (1986b as *Mastigolejeuneavirens*), Sukkharak and Gradstein (2014 as *M.virens*). Penang, Sabah.

***Treubiainsignis*** K.I.Goebel – Akiyama et al. (2001). Sabah.

***Triandrophyllumheterophyllum*** (Steph.) Grolle – Cheah (2017). Pahang.

***Trichocoleamagna*** T.Katag. – Katagiri et al. (2013). Sarawak.

***Trichocoleamollissima*** (Hook.f et Taylor) Gottsche – Katagiri et al. (2013). Sabah.

***Trichocoleapluma*** (Reinw., Blume et Nees) Mont. – De Notaris (1874), Herzog (1950), Inoue (1965), Kitagawa (1971, 1973), Kitagawa and Kodama (1973), Tixier (1974), Pocock et al. (1984), Kürschner (1990), Akiyama et al. (2001), Ludwiczuk and Asakawa (2010), Katagiri et al. (2013). Kedah, Penang, Pahang, Sarawak, Sabah.

***Trichocolearudimentaris*** Steph. – Katagiri et al. (2013). Pahang.

***Trichocoleatomentella*** (Ehrh.) Dumort. – Gottsche et al. (1845 as T.tomentellaf.javanica), Johnson (1972), Katagiri et al. (2013). Penang, Pahang, Sabah.

***Tricholepidozianeesii*** (Lindenb.) E.D.Cooper – De Notaris (1874), Herzog (1950 as *Lepidozianeesii*), Inoue (1967a, 1968b both as *Telaraneaneesii*), Kitagawa and Kodama (1973), Mizutani (1974 as *Telaraneaneesii*), Akiyama et al. (2001 as *Telaraneaneesii*), Engel and Merrill (2004 as *Telaraneaneesii*). Pahang, Sarawak, Sabah.

***Tricholepidoziaoctoloba*** (Del Ros.) E.D.Cooper – Shi and Zhu (2008 as *Telaraneaoctoloba*). Johor.

***Tricholepidoziaquadriseta*** (Steph.) E.D.Cooper – Pócs et al. (2014 as *Telaraneaquadriseta*). Pahang.

***Tricholepidoziasemperiana*** (Steph.) E.D.Cooper – Mizutani (1974b as *Telaraneasemperiana*). Sabah.

***Tricholepidoziatrichocoleoides*** (Herzog) E.D.Cooper – Herzog (1950 as *Lepidoziatrichocoleoides*), Kitagawa and Kodama (1973 as *L.trichocoleoides*), Engel and Merrill (2004 as *Telaraneatrichocoleoides*). Sarawak, Sabah.

***Tritomariaexsecta*** (Schmidel) Loeske – Kitagawa (1970). Sabah.

***Tuyamaellaangulistipa*** (Steph.) R.M.Schust. et Kachroo – Stephani (1896 as *Pycnolejeuneaangulistipa*), Schiffner (1898 as *P.angulistipa*), Mizutani (1966, 1970), Tixier (1971, 1973a), Zhu and So (2000b). Perak, Pahang, Johor, Sabah.

***Tuyamaellaborneensis*** Tixier – Tixier (1973a). Sabah.

***Tuyamaellamolischii*** (Schiffn.) S.Hatt. – Tixier (1973a), Pesiu et al. (2021). Kedah, Terengganu.

***Tuyamaellaserratistipa*** S.Hatt. – Herzog (1951 as *Pycnolejeuneaappendiculata*), Mizutani (1966), Tixier (1973a), Kitagawa and Kodama (1974), Zhu and So (1998a, 2000c), Pócs et al. (2020). Sarawak, Sabah.

***Wiesnerelladenudata*** (Mitt.) Steph. – Akiyama et al. (2001), Ludwiczuk and Asakawa (2010). Sabah.

***Zoopsisliukiuensis*** Horik. – Herzog (1950 as *Z.argentea*), Kitagawa and Kodama (1973), Manuel (1981), Grolle and Piippo (1984), Pocock et al. (1984), Mizutani and Chang (1986), Bisang et al. (2003). Pahang, Sarawak, Sabah.

***Zoopsissetigera*** K.I.Goebel – Kitagawa and Kodama (1973 as *Z.rigida*), Schuster (1999). Sarawak, Sabah.

### Hornworts (Anthocerotophyta)

***Anthocerosangustus*** Steph. – Mohamed and Yong (2005 as *A.formosae*), Mohamed et al. (2005 as *A.formosae*), Lee and Gradstein (2021). Langkawi.

***Dendroceroscavernosus*** J.Haseg. – Hasegawa (1980). Sabah.

***Dendrocerosdifficilis*** Steph. – Chantanaorrapint et al. (2014). Pahang.

***Foliocerosglandulosus*** (Lehm. et Lindenb.) D.C.Bharadwaj – Meijer (1957 as *Anthocerosgrandulosus*), Tixier (1971 as *A.grandulosus*). Pahang, Sarawak.

***Megaceros ﬂagellaris*** (Mitt.) Steph. – Herzog (1950 as *M.celebensis*), Hasegawa (1983), Lee and Gradstein (2021). Pahang, Sarawak, Sabah.

***Phaeoceroscarolinianus*** (Michx.) Prosk. – Johnson (1958 as *Anthocerosvalidus*). Perak, Pahang.

***Phaeomegacerosfoveatus*** (J.Haseg.) J.C.Villarreal – Hasegawa (2001 as *Phaeocerosfoveatus*). Sabah.

### Further records

The following records from Malaysia (Genting Highlands, Pahang; Cheah 2017) need verification.

*Cololejeuneadenticulata* (Horik.) S.Hatt.

*Cololejeuneadinghuiana* R.L.Zhu et Y.F.Wang

*Cololejeuneadrepanolejeuneoides* (Horik.) R.M.Schust.

*Cololejeuneagrossepapillosa* (Horik.) N.Kitag.

*Cololejeuneaplagiophylla* Benedix

*Cololejeuneaspinosa* (Horik.) Pandé et R.N.Misra

*Plagiochilafusca* Sande Lac.

*Plagiochilagracilis* Lindenb. et Gottsche

*Plagiochilasalacensis* Gottsche

*Plagiochilastephanii* Schiffn.

*Riccardiadiminuta* Schiffn.

*Riccardiahattorii* Furuki

*Riccardiamultifida* (L.) Gray

*Riccardiamultifidoides* Schiffn.

*Riccardiapumila* Furuki

*Riccardiasingapurensis* Schiffn.

### Doubtful records

Anastrophyllumpiligerumf.denticalyx – Reported from Sarawak (Herzog 1950); status unclear.

Aspiromitusvesiculosusvar.latifrons Herzog – Reported from Sarawak (Herzog 1950), but its status is unclear. *Aspiromitus* is now a synonym of *Anthoceros*.

*Bazzaniaeverettii* (Steph.) Meijer nom. inval. (ICN Art. 41.5) – Reported from Sarawak (Stephani 1908 as *Mastigobryumeverettii*); status unclear.

*Bazzaniakarcharias* Herzog, nom. inval. (ICN 38.1) – Sarawak (Herzog 1950).

*Bazzaniastonii* Inoue nom. inval. (ICN Art. 38.1(a) – Reported from Pahang (Inoue 1968b); the record should be checked.

*Bazzaniasubhyalina* (Steph.) Chuah-Pet. nom. inval. (ICN Art. 41.5) – Reported from Sarawak (Stephani 1924 as *Mastigobryumsubhyalinum*); status unclear.

*Cephaloziellastephanii* Douin – Reported from Pahang (Cheah 2017); the record should be checked.

*Chiloscyphusceylanicus* Tixier nom. inval. (ICN Art. 38.1a) – Reported from Kedah (Tixier 1974); the record should be checked.

*Chiloscyphusschiffneri* J.J.Engel et R.M.Schust. nom illeg. – Synonym of *Lophocoleajavanica* Schiffn., its occurrence in Pahang (Cheah 2017) is doubtful.

*Chiloscyphus ﬂaccidens* Steph. – Reported from Kedah (Tixier 1974); the record should be checked.

Drepanolejeuneablumeivar.angulistipa Herzog nom inval. (ICN Art. 39.1) – Reported from Sabah (Herzog 1936); the record should be checked.

*Drepanolejeuneamalayana* Tixier (“Grolle”) nom. inval. – Reported from Kedah (Tixier 1974); the record should be checked.

Drepanolejeuneavesiculosasubsp.propagulifera Herzog nom. inval. (ICN Art. 39.1) – Reported from Negeri Sembilan (Wolseley et al. 1996) and Pahang (Inoue 1967a); the record should be checked.

Frullaniaornithocephalaf.magnilobulaandf.retusa – Reported from Sabah (Hattori 1976), but their status is unclear.

*Gottscheaintegerrima* (Steph.) Grolle et Zijlstra – Accepted name: *Schistochilaintegerrima* Steph., only known from Vanuatu and New Caledonia (Thouvenot 2021). Its occurrence in Pahang (Cheah 2017) is doubtful and the record is most likely *S.nuda* Horik., known from Thailand and Southeast Asia.

*Radulakurzii* Steph. – Reported from Pahang (Cheah 2017); not known in tropical Southeast Asia and very similar to the widespread Asian *R.javanica* Gottsche.

*Symphyogynasimilis* Grolle – Reported from Pahang (Cheah 2017); the record should be checked, only known from the Huon Peninsula and Java (Grolle and Piippo 1986).

Telaraneawallichianavar.remotifolia Herzog – Reported from Sarawak (Herzog 1950), but its status is unclear.

### Excluded and erroneous records (See Menzel 1988 for further excluded records)

*Acrolejeuneaaulacophora* (Mont.) Steph. – The record from Sabah (Mizutani 1969 as *Ptychocoleusaulacophorus*) is doubtful.

*Acrolejeuneaborneensis* Bonner, nom. inval. from Sarawak (Bonner 1962) is a *Schiffneriolejeunea* sp. fide Gradstein (1975).

*Acrolejeuneasandvicensis* (Gottsche) J.Wang et Gradst. – The record from Malaysia (Tixier (1975 as *Brachiolejeuneasandvicensis*) belongs to *Thysananthusreconditus* (Sun et al. 2018).

*Bazzaniatricrenata* (Wahlenb.) Lindb. – This is a Holarctic species, not known in tropical Southeast Asia, and its occurence in Malaysia (Pahang; Cheah 2017) is considered erroneous. Also reported from Sabah by Sande Lacoste (1864 as *Mastigobryumdeflexum*).

*Cheilolejeuneaparvidens* B.M.Thiers – The record from Sabah (Pócs et al. 2020) belongs to *C.intertexta*.

*Chiloscyphusminor* (Nees) J.J.Engel et R.M.Schust. – This is a Holarctic species, not known in tropical Southeast Asia, and its occurence in Malaysia (Pahang; Cheah 2017) is considered erroneous.

Frullaniaintegristipulavar.emarginata Verd. – Reported from Sabah by Chuah-Petiot (2011) based on Hattori (1982), but the latter author did not report this variety from Malaysia.

*Frullaniapulogensis* Steph. – Reported from Sabah by Verdoorn (1932a) but it was rejected by Hattori (1978) due to the scarcity of the specimen. No material of *F.pulogensis* but fragmental shoots of *F.mizutanii* are present.

*Kurziamauiensis* (H.A.Mill.) H.A.Mill. – Reported from Pahang (Cheah 2017). Only known from the Pacific Islands and its occurrence in Malaysia needs verification.

*Lejeuneadentata* Mitt. – Reported from Pahang (Cheah 2017). Synonym of *Radulacuspidata* Steph., only known in New Zealand.

*Lejeuneaulicina* (Taylor) Gottsche, Lindenb. et Nees – Accepted name: *Microlejeuneaulicina* (Taylor) Steph., a Holarctic species, not known in tropical Southeast Asia. Its occurrence in Malaysia (Pahang; Cheah 2017) is considered erroneous.

*Metacalypogeiaalternifolia* (Nees) Grolle – Reported from Sarawak by Herzog (1950 as *Calypogeiaalternifolia*). According to Grolle (1964), this record is erroneous.

*Metzgeriaalbinea* Spruce – This is a Neotropical species according to So (2002), and its occurrence in Malaysia (Pahang; Cheah 2017) needs verification.

*Mniolomastamatotonum* M.A.M.Renner et E.A.Br. – Reported from Pahang (Cheah 2017); an Australasian species, not known in tropical Southeast Asia.

*Ricciatreubiana* Steph. – Reported from Peninsular Malaysia by Chuah Petiot (2011) based on Meijer (1958) but the latter author reported this species from Singapore. *Ricciatreubiana* is a common species and often found in gardens, recreational parks and secondary forests. Its occurrence in Malaysia is to be expected.

*Odontoschismadenudatum* (Nees) Dumort. – Reported from Sabah by various authors (see Chuah-Petiot 2011), but the material belongs to O.denudatumsubsp.naviculare (see Gradstein and Ilkiu-Borges 2015).

*Odontoschismasphagni* (Dicks.) Dumort. – Reported from Sabah by Mitten and Wright (1894), but the record is considered erroneous (Váňa 1993). According to Gradstein and Ilkiu-Borges (2015)*O.sphagni* is a species from Europe and North America that does not occur in Asia.

*Saccogynidiumirregularospinum* C.Gao, T.Cao et M.J.Lai – Reported from Pahang (Cheah 2017); a temperate Asiatic species from Sikkim and China, not known in tropical Southeast Asia.

*Schistochilanyamanii* – Reported from Pahang (Cheah 2017), but the species name does not exist.

*Solenostomaobliquifolium* (Schiffn.) Váňa, Hentschel et Heinrichs – Reported from Sabah by Chuah-Petiot (2011) based on Váňa (1991b as *Jungermanniaobliquifolia*) but the latter author did not report this species in Sabah.

*Telaraneagranulata* J.J.Engel et G.L.Merr. – Reported from Pahang (Cheah 2017); endemic to New Zealand, not known in tropical Southeast Asia.

*Telaraneapatentissima* (Hook.f et Taylor) E.A.Hodgs. – Reported from Pahang (Cheah 2017); an Australasian species from Australia and New Zealand, not known in tropical Southeast Asia.

*Thysananthusauriculatus* (Wilson et Hook.) Sukkharak et Gradst. – Reported from Pahang by Lee et al. (2013 as *Mastigolejeuneaauriculata*). According to Sukkharak and Gradstein (2014, 2017) *T.auriculatus* is an Afro-American species; Asiatic records of this species belong to *T.humilis*.

*Wettsteiniainversa* (Sande Lac.) Schiffn. – Reported from Sabah by Frahm et al. (1990) and Chuah-Petiot (2011) based on Menzel (1988), but the latter author reported this species only from Kalimantan Selatan (leg. Korthals, paratype of *Plagiochilascabra* Sande Lac.). *Wettsteiniainversa* is a widespread Malesian species (see Piippo 1984) and its occurrence in Malaysia is to be expected.

### Synonyms

New synonymy published after the appearance of World Checklist (Söderström et al. 2016) is provided with a reference.

*Acantholejeuneaspinistipula* (Herzog) R.M.Schust. ≡ *Ceratolejeuneaspinistipula* (Herzog) R.L.Zhu, L.Shu, Qiong He et Y.M.Wei

*Acrolejeuneapulopenangensis* (Gottsche) Schiffn. ≡ *Schiffneriolejeuneapulopenangensis* (Gottsche) Gradst.

*Acrolejeuneatumida* (Nees) Steph. ≡ *Schiffneriolejeuneatumida* (Nees) Gradst.

*Acromastigumbidenticulatum* A.Evans = *A.bancanum* (Sande Lac.) A.Evans

*Acromastigumdenticulatum* A.Evans = *A.bifidum* (Steph.) A.Evans

*Acromastigumemarginatum* Herzog ≡ *Bazzaniamenzelii* E.D.Cooper

*Acromastigumgemmiferum* N.Kitag. et T.Kodama = *A.obliquatum* (Mitt.) A.Evans

*Adelanthusborneensis* Grolle ≡ *Pseudomarsupidiumborneensis* (Grolle) Váňa, L.Söderstr., A.Hagborg et von Konrat

*Allorgellachangiana* Tixier = *Otolejeuneasemperiana* (Steph.) Grolle

*Anastrophyllumappendiculatum* N.Kitag = *A.auritum* (Lehm.) Steph.

*Anastrophyllumdivergens* Herzog = *A.fissum* Steph.

*Anastrophyllumimbricatum* (Gottsche, Lindenb. et Nees) Steph. = *A.piligerum* (Nees) Steph.

*Anastrophyllumminutum* (Schreb.) R.M.Schust. ≡ *Sphenolobusminutus* (Schreb.) Berggr.

*Aneurasubcrenulata* Herzog = *Riccardiaparvula* Schiffn.

*Anomacaulisflaccidus* (Steph.) Grolle ≡ *Syzygiellaflaccida* (Steph.) Gradst. et G.E.Lee (Lee and Gradstein 2021)

*Anthocerosformosae* Steph. = *A.angustus* Steph.

*Anthocerosglandulosus* Lehm. et Lindenb. ≡ *Foliocerosglandulosus* (Lehm. et Lindenb.) Bhardwaj

*Anthocerosvalidus* Steph. = *Phaeoceroscarolinianus* (Michx.) Prosk.

*Aploziapolyrhiza* (Hook.) Horik. = *Solenostomatruncatum* (Nees) Váňa et D.G.Long

Apometzgeriapubescensvar.kinabaluensis Kuwah. = *Metzgeriakinabaluensis* (Kuwah.) Masuzaki

*Aphanolejeuneaborneensis* (Herzog) Pócs = *Cololejeuneapapillosa* (K.I.Goebel) Mizut.

Aphanolejeuneamicroscopicavar.borneensis Herzog = *Cololejeuneapapillosa* (K.I.Goebel) Mizut.

*Aphanolejeuneaproboscoidea* (K.I.Goebel) R.M.Schust. = *Cololejeuneadiaphana* A.Evans

*Arachniopsismajor* Herzog ≡ *Telaraneamajor* (Herzog) J.J.Engel et G.L.Merr.

*Arachniopsismonocera* R.M.Schust. et Grolle ≡ *Telaraneamonocera* J.J.Engel et G.L.Merr.

*Archilejeuneamariana* auct. (non (Gottsche) Steph.) = *Spruceanthusplaniusculus* (Mitt.) X.Q.Shi, R.L.Zhu et Gradst. (Shi et al. 2015)

*Archilejeuneaplaniuscula* (Mitt.) Steph. ≡ *Spruceanthusplaniusculus* (Mitt.) X.Q.Shi, R.L.Zhu et Gradst. (Shi et al. 2015)

*Austrometzgeriafrancana* (Steph.) Kuwah. ≡ *Metzgeriafrancana* Steph.

*Bazzaniaassamica* (Steph.) S.Hatt. = B.tridensvar.assamica (Steph.) Pócs

*Bazzaniaaustralis* Gottsche, Lindenb. et Nees = *B.tridens* (Reinw., Blume et Nees) Trevis.

*Bazzaniabancana* (Sande Lac.) Trevis. ≡ *Acromastigumbancanum* (Sande Lac.) A.Evans

*Bazzaniaconcinna* (De Not.) Trevis. = *B.intermedia* (Gottsche et Lindenb.) Trevis.

*Bazzaniacoreana* Herzog = *B.tridens* (Reinw., Blume et Nees) Trevis.

*Bazzaniaechinatiformis* (De Not.) Trevis. ≡ *Acromastigumechinatiforme* (De Not.) A Evans

*Bazzaniaferox* (De Not.) Trevis. = *B.harpago* (De Not.) Schiffn.

*Bazzaniaremotifolia* Herzog nom. illeg. ≡ *B.herzogiana* Meijer

*Bazzaniapalmatifida* (Steph.) Grolle = *B.subtilis* (Sande Lac.) Trevis.

Bazzaniafallaxf.fissa Herzog = *B.fallax* (Sande Lac.) Schiffn.

*Bazzanialingana* (De Not.) Trevis. ≡ *Acromastigumlinganum* (De Not.) A.Evans

*Bazzanianotarisii* (Steph.) Schiffn. = *Acromastiguminaequilaterum* (Lehm. et Lindenb.) A.Evans

*Bazzaniapulvinulata* (De Not.) Schiffn. ≡ *Mastigopelmapulvinulatum* (De Not.) Grolle

Bazzaniarecurvavar.pallens (De Not.) Schiffn. = *B.recurva* (Mont.) Trevis.

*Bazzaniarecurvolimbata* (Steph.) N.Kitag. = *B.revoluta* (Steph.) N.Kitag.

*Bazzaniarichardsii* Herzog = *B.calcarata* (Sande Lac.) Schiffn.

*Bazzaniaserrulata* (Mitt.) Steph. = *B.erosa* (Reinw., Blume et Nees) Trevis.

Bazzaniatridensf.minutissima (Kamim.) Pócs = *B.tridens* (Reinw., Blume et Nees) Trevis.

*Bazzaniavaga* (De Not.) Trevis. = *B.erosa* (Reinw., Blume et Nees) Trevis.

*Calypogeiabaldwinii* Austin = *Mniolomafuscum* (Lehm.) R.M.Schust.

*Calypogeiafusca* (Lehm.) Steph. ≡ *Mniolomafuscum* (Lehm.) R.M.Schust.

*Campylolejeuneapeculiaris* (Herzog) Amakawa = *Cololejeuneainflectens* (Mitt.) Benedix

*Campylolejeuneapusilla* Mizut. ≡ *Cololejeuneahattoriana* Mizut. et Pócs

*Caudalejeuneacircinata* Steph. = *C.cristiloba* (Steph.) Gradst.

*Caudalejeuneaserrata* Steph. = *C.reniloba* (Gottsche) Steph.

*Caudalejeunearecurvistipula* (Gottsche) Schiffn. = *C.reniloba* (Gottsche) Steph.

*Caudalejeuneastephanii* Steph. nom. inval. = *C.reniloba* (Gottsche) Steph.

*Cephaloziapearsonii* (Spruce) Steph. ≡ *Sphenolobopsispearsonii* (Spruce) R.M.Schust.

*Cephaloziellatagawae* N.Kitag. = *Cylindrocoleatagawae* (N.Kitag.) R.M.Schust.

*Cephaloziellawillisana* (Steph.) N.Kitag. = *Cylindrocoleakiaeri* (Austin) Douin

*Ceratolejeuneaoceanica* (Mitt.) Steph. = *C.belangeriana* (Gottsche) Steph.

*Ceratolejeunearyukyuensis* Amakawa = *C.belangeriana* (Gottsche) Steph.

*Ceratolejeuneatahitensis* Steph. = *C.cornuta* (Lindenb.) Steph.

*Chandonanthusfiliformis* Steph. ≡ *Tetralophoziafiliformis* (Steph.) Urmi

*Chandonanthushirtellus* (F.Weber) Mitt. ≡ *Plicanthushirtellus* (F.Weber) R.M.Schust.

*Chandonanthuspilifer* Steph. ≡ *Tetralophoziapilifera* (Steph.) R.M.Schust.

*Cheilolejeuneaexcisula* (Steph.) Mizut. = *C.incisa* (Gottsche) R.M.Schust.

*Cheilolejeuneaimbricata* (Nees) S.Hatt. = *C.trapezia* (Nees) R.M.Schust. et Kachroo

*Cheilolejeunealarsenii* Mizut. = C.adnatavar.autoica Gradst. et Ilk.-Borg. (Bastos and Gradstein 2020).

*Cheilolejeunealongiloba* (G.Hoffm.) R.M.Schust. = *C.trapezia* (Nees) R.M.Schust. et Kachroo

*Cheilolejeunealuerssenii* (Steph.) Mizut. = *C.lindenbergii* (Gottsche) Mizut.

*Cheilolejeuneameyeniana* (Nees, Lindenb. et Gottsche) R.M.Schust. et Kachroo = *C.trapezia* (Nees) R.M.Schust. et Kachroo

*Cheilolejeuneapicta* Mizut. ≡ *Pictolejeuneamizutanii* Grolle

*Cheilolejeuneaspathulata* Mizut. = *C.decursiva* (Sande Lac.) R.M.Schust.

*Cheilolejeuneaviridula* (Herzog) Mizut. = *C.trifaria* (Reinw., Blume et Nees) Mizut.

*Chiloscyphusacutangulus* Schiffn. ≡ *Heteroscyphusacutangulus* (Schiffn.) Schiffn.

*Chiloscyphusargutus* (Reinw., Blume et Nees) Nees ≡ *Heteroscyphusargutus* (Reinw., Blume et Nees) Schiffn.

*Chiloscyphusaselliformis* (Reinw., Blume et Nees) Nees ≡ *Heteroscyphusaselliformis* (Reinw., Blume et Nees) Schiffn.

Chiloscyphusaselliformisvar.neesii Schiffn. = *Heteroscyphusaselliformis* (Reinw., Blume et Nees) Schiffn.

*Chiloscyphusciliolatus* (Nees) J.J.Engel et R.M.Schust. ≡ *Cryptolophocoleaciliolata* (Nees) L.Söderstr., Crand.-Stotl., Stotler et Váňa

*Chiloscyphuscoadunatus* (Sw.) J.J.Engel et R.M.Schust. = *Lophocoleabidentata* (L.) Dumort.

*Chiloscyphuscommunis* Steph. = *Heteroscyphuscoalitus* (Hook.) Schiffn.

*Chiloscyphusconcinnus* De Not. = *Heteroscyphussucculentus* (Gottsche) Schiffn.

*Chiloscyphuscostatus* (Nees) J.J.Engel et R.M.Schust. ≡ *Cryptolophocoleacostata* (Nees) L.Söderstr.

*Chiloscyphuscuspidatus* (Nees) J.J.Engel et R.M.Schust. = *Lophocoleabidentata* (L.) Dumort.

*Chiloscyphusdecurrens* (Reinw., Blume et Nees) Nees = *Heteroscyphussplendens* (Lehm. et Lindenb.) Grolle

*Chiloscyphusdensifolius* De Not. = *Heteroscyphussplendens* (Lehm. et Lindenb.) Grolle

*Chiloscyphusdiestianus* Sande Lac. ≡ *Heteroscyphusdiestianus* (Sande Lac.) Piippo

*Chiloscyphusintegerrimus* Schiffn. ≡ *Heteroscyphusintegerrimus* (Schiffn.) Gradst. et G.E.Lee (Lee and Gradstein 2021).

*Chiloscyphuskurzii* (Sande Lac.) J.J.Engel et R.M.Schust. ≡ *Lophocoleakurzii* Sande Lac.

*Chiloscyphuslatifolius* (Nees) J.J.Engel et R.M.Schust. = *Lophocoleabidentata* (L.) Dumort.

*Chiloscyphusmuricatus* (Lehm.) J.J.Engel et R.M.Schust. ≡ *Lophocoleamuricata* (Lehm.) Nees

*Chiloscyphusmuricellus* De Not. ≡ *Saccogynidiummuricellum* (De Not.) Grolle

*Chiloscyphusparoicus* J.J.Engel et R.M.Schust. ≡ *Cryptolophocolealevieri* (Schiffn.) L.Söderstr.

*Chiloscyphusparvulus* Schiffn. ≡ *Heteroscyphusparvulus* (Schiffn.) Schiffn.

*Chiloscyphussikkimensis* (Steph.) J.J.Engel et R.M.Schust. ≡ *Lophocoleasikkimensis* (Steph.) Herzog et Grolle

*Chiloscyphussteetziae* (De Not.) J.J.Engel et R.M.Schust. ≡ *Lophocoleasteetziae* De Not.

*Chiloscyphussucculentus* Gottsche ≡ *Heteroscyphussucculentus* (Gottsche) Schiffn.

*Chiloscyphuswettsteinii* Schiffn. ≡ *Heteroscyphuswettsteinii* (Schiffn.) Schiffn.

Chiloscyphuszollingerivar.borneensis Herzog = *Heteroscyphuszollingeri* (Gottsche) Schiffn.

*Cololejeuneaandrogyna* Benedix = *C.obliqua* (Nees et Mont.) Schiffn.

*Cololejeuneaamoena* Benedix ≡ C.floccosavar.amoena (Benedix) Pócs

*Cololejeuneaastyla* Mizut. = *C.platyneura* (Spruce) A.Evans

*Cololejeuneabalansae* (Steph.) Mizut. = *C.trichomanis* (Gottsche) Steph.

*Cololejeuneabenedixii* Tixier = *C.schmidtii* Steph.

*Cololejeuneabolombensis* (Steph.) Vanden Berghen = *C.lanciloba* Steph.

*Cololejeuneaciliatilobula* (Schiffn.) Schiffn. = *C.inﬂectens* (Mitt.) Benedix

*Cololejeuneacrenulata* (Pearson) H.A.Mill. = *C.angustiﬂora* (Steph.) Mizut.

*Cololejeuneafalcatoides* Benedix = *C.falcata* (Horik.) Benedix

*Cololejeuneafilicaulis* Steph. = *C.hildebrandii* (Austin) Steph.

*Cololejeuneaformosana* Mizut. = *C.ceratilobula* (P.C.Chen) R.M.Schust.

*Cololejeuneagoebelii* (Schiffn.) Schiffn. = *C.trichomanis* (Gottsche) Steph.

*Cololejeuneainflectidens* (Mitt.) Benedix nom. inval. ≡ *C.inflectens* (Mitt.) Benedix

*Cololejeuneajavanica* (Steph.) Mizut. = *C.angustiﬂora* (Steph.) Mizut.

*Cololejeuneajelinekii* Steph. = *C.obliqua* (Nees et Mont.) Schiffn.

*Cololejeuneakarstenii* K.I.Goebel = *C.cordiflora* Steph.

*Cololejeuneakinabalensis* Mizut. = *C.macounii* (Underw.) A.Evans

*Cololejeunealeonidens* Benedix = *C.ocelloides* (Horik.) Mizut.

Cololejeunealeonidensvar.saccata Benedix = *C.ocelloides* (Horik.) Mizut.

*Cololejeuneamackeeana* Tixier = *C.angustiﬂora* (Steph.) Mizut.

*Cololejeuneameijeri* Tixier = *C.ocelloides* (Horik.) Mizut.

Cololejeuneamutabilisf.borneensis Benedix = *C.mutabilis* Benedix

Cololejeuneamutabilisf.floccodoides Tixier = *C.mutabilis* Benedix

Cololejeuneamutabilisf.subfalcata Tixier = *C.mutabilis* Benedix

*Cololejeuneanymannii* (Steph.) Benedix = *C.obliqua* (Nees et Mont.) Schiffn.

*Cololejeuneaoshimensis* (Horik.) Benedix = *C.inﬂata* Steph.

*Cololejeuneapahangiana* Tixier = *C.lanciloba* Steph.

*Cololejeuneapakseana* Tixier = *C.uchimae* Amakawa

*Cololejeuneaparoica* Mizut. = *C.gottschei* (Steph.) Mizut.

*Cololejeuneapeculiaris* (Herzog) Benedix = *C.inflectens* (Mitt.) Benedix

Cololejeuneaperaffinisvar.elegans Benedix = *C.perafﬁnis* (Schiffn.) Schiffn.

*Cololejeuneaplaniflora* Benedix = *Cololejeuneaindica* Pandé et R.N.Misra

*Cololejeuneapluripunctata* Benedix = *C.siamensis* Steph.

*Cololejeuneapusilla* Steph. = *C.gradsteinii* M.J.Lai et R.L.Zhu

*Cololejeuneascabriflora* Steph = *C.obliqua* (Nees et Mont.) Schiffn.

Cololejeuneatenellavar.vittata Tixier = *C.tenella* Benedix

Cololejeuneatrichomanissubsp.cordiflora (Steph.) Pócs ≡ *C.cordiflora* Steph.

*Cololejeuneayulensis* (Steph.) Benedix = *C.aequabilis* (Sande Lac) Schiffn.

*Coluraacutifolia* Jovet-Ast = *C.conica* (Sande Lac.) K.I.Goebel

*Coluraapiculata* (Schiffn.) Steph. = *C.leratii* (Steph.) Steph.

*Colurajavanica* Steph. = *C.ari* (Steph.) Steph.

*Coluratrialata* (Steph.) Herzog et Zwickel = *C.corynophora* (Nees, Lindenb. et Gottsche) Trevis.

*Colurolejeuneaornata* A.Evans ≡ *Coluraornata* K.I.Goebel

*Conoscyphusinflexifolius* Mitt. = *C.trapezioides* (Sande Lac.) Schiffn.

*Crossotolejeuneaborneensis* Herzog = *Lejeuneastenodentata* M.A.M.Renner et Pócs

*Cryptochilagrandiﬂora* (Lindenb. et Gottsche) Grolle = *Syzygiellasonderi* (Gottsche) K.Feldberg, Váňa, Hentschel et Heinrichs

*Cuspidatulacontracta* (Reinw., Blume et Nees) Steph. ≡ *Syzygiellacontracta* (Reinw., Blume et Nees) Gradst. et G.E.Lee (Lee and Gradstein 2021)

*Cylindrocoleakiaeri* (Austin) Váňa ≡ *Cephaloziellakiaeri* (Austin) Douin (Lee and Gradstein 2021)

*Diplasiolejeuneabrachyclada* A.Evans = *D.cavifolia* Steph.

*Diplasiolejeuneaincurvata* Ast et Tixier = *D.cobrensis* Steph.

*Diplasiolejeuneajavanica* Steph. = *D.cavifolia* Steph.

*Diplasiolejeuneaneobrachyclada* S.Hatt = *D.patelligera* Herzog

*Diplasiolejeunearudolphiana* Steph. = *D.unidentata* (Lehm. et Lindenb.) Schiffn.

*Diploscyphusborneensis* De Not. = *Conoscyphustrapezioides* (Sande Lac.) Schiffn.

*Drepanolejeuneabrunnea* Mizut. ≡ *Mohamediabrunnea* (Mizut.) R.L.Zhu et L.Shu

*Drepanolejeuneadentata* Steph. ≡ *Lejeuneastenodentata* M.A.M.Renner et Pócs

*Drepanolejeuneafilicuspis* (Steph.) Mizut. ≡ *Microlejeuneafilicuspis* (Steph.) Heinrichs, Schäf.-Verw., Pócs et S.Dong

Drepanolejeunealevicornuavar.longicornua Herzog = *D.longicornua* (Herzog) Mizut.

*Drepanolejeuneamicholitzii* Steph. = *D.pentadactyla* (Mont.) Steph.

Drepanolejeuneamicholitziivar.angustissima Herzog = *D.pentadactyla* (Mont.) Steph.

Drepanolejeuneamicholitziivar.brevifolia Herzog = *D.pentadactyla* (Mont.) Steph.

Drepanolejeuneamicholitziivar.dactylophoroides Herzog ≡ D.pentadactylavar.dactylophoroides (Herzog) Pócs

*Drepanolejeuneamuricata* (Gottsche) Schiffn. ≡ *Dactylophorellamuricata* (Gottsche) R.M.Schust.

*Drepanolejeuneaspinistipula* Herzog ≡ *Ceratolejeuneaspinistipula* (Herzog) R.L.Zhu, L.Shu, Qiong He et Y.M.Wei

Drepanolejeuneateneraf.goebelii Herzog = *D.tenera* K.I.Goebel

Drepanolejeuneateneravar.genuina Herzog nom. illeg. = *D.tenera* K.I.Goebel

*Drepanolejeuneatenuis* (Nees) Schiffn. = *D.angustifolia* (Mitt.) Grolle

Drepanolejeuneaternatensisvar.lancispina Herzog = *D.ternatensis* (Gottsche) Schiffn.

Drepanolejeuneavesiculosaf.robusta Herzog = *D.vesiculosa* (Mitt.) Steph.

*Dumortieracalcicola* Campb. = D.hirsutasubsp.nepalensis (Taylor) R.M.Schust.

*Dumortieranepalensis* (Taylor) Nees ≡ D.hirsutasubsp.nepalensis (Taylor) R.M.Schust.

*Dumortieratrichocephala* (Hook.) Nees = D.hirsutasubsp.nepalensis (Taylor) R.M.Schust.

*Eopleuroziasimplicissima* (Herzog) R.M.Schust. = *Pleuroziagigantea* (F.Weber) Lindb.

*Euosmolejeunealuerssenii* Steph. = *Cheilolejeunealindenbergii* (Gottsche) Mizut.

*Frullaniaaltemammillata* S.Hatt = *F.armatifolia* Verd.

*Frullaniaborneensis* Steph. = *F.sublignosa* Steph.

*Frullaniahampeana* Nees = *F.monocera* (Hook.f. et Taylor) Gottsche, Lindenb. et Nees

Frullaniahasskarlianavar.integribracteata (Verd.) S.Hatt. = *F.hasskarliana* Lindenb.

Frullaniaintermediaf.billardieriana (Nees et Mont.) Verd. = *F.intermedia* (Reinw., Blume et Nees) Nees

Frullaniaintermediavar.submorokensis S.Hatt = *F.intermedia* (Reinw., Blume et Nees) Nees

Frullaniajunghuhnianavar.minutissima Verd. = F.junghuhnianavar.tenella (Sande Lac.) Grolle et S.Hatt.

*Frullaniaminor* Sande Lac. = *F.gracilis* (Reinw., Blume et Nees) Nees

Frullaniamoniliatasubsp.breviramea (Steph.) Verd. = *F.moniliata* (Reinw., Blume et Nees) Mont.

Frullaniaornithocephalaf.magnilobula S.Hatt. = *F.ornithocephala* (Reinw., Blume et Nees) Nees

Frullaniaornithocephalaf.retusa S.Hatt. = *F.ornithocephala* (Reinw., Blume et Nees) Nees

*Frullaniaperminuta* S.Hatt. = F.junghuhnianavar.tenella (Sande Lac.) Grolle et S.Hatt.

*Frullaniaperversa* Steph. = F.junghuhnianavar.tenella (Sande Lac.) Grolle et S.Hatt.

*Frullaniaphilippinensis* Steph. = *F.reﬂexistipula* Sande Lac.

*Frullaniapicta* Steph. = *F.trichodes* Mitt.

Frullaniarepandistipulasubsp.spinifera S.Hatt. nom. inval. = F.repandistipulasubsp.spinibractea S.Hatt.

Frullaniaserrataf.crispulodentata Verd. = *F.serrata* Gottsche

*Frullaniasquarrosa* (Mont.) Nees = *F.ericoides* (Nees) Mont.

Frullaniatamariscivar.breviramea (Steph.) S.Hatt. = *F.moniliata* (Reinw., Blume et Nees) Mont.

*Frullaniatenuicaulis* Mitt. = *F.trichodes* Mitt.

*Frullaniavethii* Sande Lac. = *F.trichodes* Mitt.

Frullaniavethiif.acutiloba S.Hatt. = *F.trichodes* Mitt.

*Frullaniawallichiana* Mitt = *F.obscura* (Sw.) Mont.

*Gottscheaaligera* (Nees et Blume) Nees ≡ *Schistochilaaligera* (Nees et Blume) J.B.Jack et Steph.

*Gottscheaaligeriformis* De Not. = *Schistochilaaligera* (Nees et Blume) J.B.Jack et Steph.

*Gottscheabeccariana* De Not. ≡ *Schistochilabeccariana* (De Not.) Trevis.

*Gottscheadoriae* De Not. ≡ *Schistochiladoriae* De Not) Trevis.

*Gottscheagaudichaudii* Gottsche = *Schistochilaaligera* (Nees et Blume) J.B.Jack et Steph.

*Gottscheaphilippinensis* Mont. = *Schistochilaaligera* (Nees et Blume) J.B.Jack et Steph.

*Gottscheasciurea* (Nees) Sande Lac. ≡ *Schistochilasciurea* (Nees) Schiffn.

Gymnomitrionlaceratumvar.borneense N.Kitag. = *G.incompletum* (Gottsche) Váňa

*Harpalejeuneaconstricta* Grolle ≡ *Microlejeuneaconstricta* (Grolle) Grolle

*Harpalejeuneafilicuspis* (Steph.) Mizut. ≡ *Microlejeuneafilicuspis* (Steph.) Heinrichs, Schäf.-Verw., Pócs et S.Dong

*Harpalejeuneakinabaluensis* Mizut. ≡ *Microlejeuneakinabaluensis* (Mizut.) Grolle

*Harpalejeuneamammillosa* Mizut. ≡ *Microlejeuneamammillosa* (Mizut.) Grolle

*Harpalejeuneaminutissima* Mizut. ≡ *Microlejeuneaminutissima* (Mizut.) Grolle

*Harpalejeuneariddleana* (Steph.) Mizut. = *Microlejeuneafilicuspis* (Steph.) Heinrichs, Schäf.-Verw., Pócs et S.Dong

*Harpalejeuneaspinosa* Mizut. ≡ *Microlejeuneaspinosa* (Mizut.) Grolle

*Hattoriellasubcrispa* (Herzog) Bakalin ≡ *Mesoptychiasubcrispa* (Herzog) L.Söderstr. et Váňa

*Herbertusarmitanus* (Steph.) H.A.Mill. = *H.sendtneri* (Nees) Lindb.

*Herbertuscircinatus* (Steph.) H.A.Mill. = *H.sendtneri* (Nees) Lindb.

*Herbertusdicranus* (Taylor) Trevis. = *H.aduncus* (Dicks.) Gray

*Herbertuslongispinus* J.B.Jack et Steph. = *H.aduncus* (Dicks.) Gray

*Herpetiumrecurvum* Mont. ≡ *Bazzaniarecurva* (Mont.) Trevis.

*Heteroscyphusbescherellei* (Steph.) S.Hatt. = *H.coalitus* (Hook.) Schiffn.

*Heteroscyphuscommunis* (Steph.) Schiffn. = *H.coalitus* (Hook.) Schiffn.

*Hygrobiellamollusca* (De Not.) Steph. ≡ *Cephaloziamollusca* (De Not.) Váňa

Hygrobiellamolluscaf.subintegerrima Herzog = *Cephaloziastolonacea* (Herzog) R.M.Schust.

*Hygrolejeunealindenbergii* (Gottsche) Steph. ≡ *Cheilolejeunealindenbergii* (Gottsche) Mizut.

*Iwatsukiaexigua* N.Kitag. = *Odontoschismajishibae* (Steph.) L.Söderstr. et Váňa

*Jamesoniellaautumnalis* (DC.) Steph. ≡ *Syzygiellaautumnalis* (DC.) K.Feldberg, Váňa, Hentschel et Heinrichs

*Jamesoniellacontracta* (Reinw., Blume et Nees) N.Kitag. ≡ *Syzygiellacontracta* (Reinw., Blume et Nees) Gradst. et G.E.Lee (Lee and Gradstein 2021)

*Jamesoniella ﬂexicaulis* (Nees) Schiffn. ≡ *Syzygiellaflexicaulis* (Nees) Gradst. et G.E.Lee (Lee and Gradstein 2021)

Jamesoniellaflexicaulisf.affinis (Schiffn.) Amakawa = *Denotarisialinguifolia* (De Not.) Grolle

Jamesoniellaflexicaulisvar.mizutanii Amakawa = *Syzygiellaflexicaulis* (Nees) Gradst. et G.E.Lee (Lee and Gradstein 2021)

*Jamesoniellamicrophylla* (Gottsche, Lindenb. et Nees) Schiffn. = *Gottscheliaschizopleura* (Spruce) Grolle

*Jamesoniellaminutissima* Amakawa = *Andrewsianthusmizutanii* N.Kitag.

*Jamesoniellanipponica* S.Hatt. ≡ *Syzygiellanipponica* (S.Hatt.) K.Feldberg, Váňa, Hentschel et Heinrichs

*Jamesoniellaovifolia* (Schiffn.) Schiffn. = *Denotarisialinguifolia* (De Not.) Grolle

*Jamesoniellaperverrucosa* Amakawa = *Syzygiellanipponica* (S.Hatt.) K.Feldberg, Váňa, Hentschel et Heinrichs

*Jamesoniellapulchra* N.Kitag. = *Denotarisialinguifolia* (De Not.) Grolle

*Jenseniazollingeri* (Gottsche) Grolle = *J.decipiens* (Mitt.) Grolle

Jubulahutchinsiaesubsp.javanica (Steph.) Verd. = *J.javanica* Steph.

*Jungermanniaappressifolia* Mitt. ≡ *Solenostomaappressifolium* (Mitt.) Váňa

*Jungermanniaariadne* Taylor ≡ *Solenostomaariadne* (Taylor) Váňa et D.G.Long

*Jungermanniaborneensis* Amakawa ≡ *Solenostomaborneense* (Amakawa) Váňa, Hentschel et Heinrichs

*Jungermanniacomata* Nees ≡ *Solenostomacomatum* (Nees) C.Gao

Jungermanniacurvifoliavar.borneensis De Not. ≡ *Nowelliaborneensis* (De Not.) Schiffn.

*Jungermanniadecolyana* Steph. = *Solenostomaappressifolium* (Mitt.) Váňa

*Jungermanniagrollei* Amakawa = *Solenostomariclefii* Váňa et D.G.Long

*Jungermanniahaskarliana* (Nees) Mitt. ≡ *Solenostomahaskarlianum* (Nees) Váňa et D.G.Long

*Jungermanniaherzogiana* Váňa ≡ *Solenostomajavanicum* (Schiffn.) Steph.

*Jungermanniaiwatsukii* Amakawa = *Syzygiellasonderi* (Gottsche) K.Feldberg, Váňa, Hentschel et Heinrichs

*Jungermanniakinabalensis* Amakawa = *Solenostomaappressifolium* (Mitt.) Váňa

*Jungermanniamizutanii* Amakawa = *Solenostomastephanii* (Schiffn.) Steph.

*Jungermanniamollusca* De Not. ≡ *Cephaloziamollusca* (De Not.) Váňa

*Jungermanniapiligera* Nees ≡ *Anastrophyllumpiligerum* (Nees) Steph.

*Jungermanniapolyrhiza* Lehm. et Lindenb. = *Solenostomatruncatum* (Nees) Váňa et D.G.Long

*Jungermanniapseudocyclops* Inoue ≡ *Solenostomapseudocyclops* (Inoue) Váňa et D.G.Long

*Jungermanniasphaerocarpa* Hook. ≡ *Solenostomasphaerocarpum* (Hook.) Steph.

*Jungermanniastephanii* (Schiffn.) Amakawa ≡ *Solenostomastephanii* (Schiffn.) Steph.

*Jungermanniastricta* (Schiffn.) Steph. ≡ *Solenostomastrictum* (Schiffn.) Váňa, Hentschel et Heinrichs

*Jungermanniatetragona* Lindenb. ≡ *Solenostomatetragonum* (Lindenb.) Váňa et D.G.Long

*Jungermanniatruncata* Nees ≡ *Solenostomatruncatum* (Nees) Váňa et D.G.Long

*Jungermanniatuberculifera* (Herzog) Váňa ≡ *Solenostomatuberculiferum* (Herzog) Váňa, Hentschel et Heinrichs

*Jungermanniavirgata* (Mitt.) Steph. ≡ *Solenostomavirgatum* (Mitt.) Váňa et D.G.Long

*Lejeuneaanisophylla* Mont. = *L.adpressa* Nees (Gradstein 2021)

*Lejeuneaboninensis* Horik. = *L.adpressa* Nees (Gradstein 2021)

*Lejeuneaborneensis* Steph. = *L.adpressa* Nees (Gradstein 2021)

*Lejeuneacaespitosa* Lindenb. = *L.adpressa* Nees (Gradstein 2021)

*Lejeuneachalmersii* (Steph.) Mizut. = *L.microloba* Taylor

*Lejeuneacomosa* (Lindenb.) Mitt. = *Thysananthuscomosus* Lindenb.

*Lejeuneacucullata* (Reinw., Blume et Nees) Nees ≡ *Metalejeuneacucullata* (Reinw., Blume et Nees) Grolle

*Lejeuneacuculliflora* (Steph.) Mizut. = *L.umbilicata* (Nees) Nees

*Lejeuneadiversitexta* (Steph.) Mizut. nom illeg. = *L.papilionacea* Prantl

*Lejeuneagoebelii* Schiffn. = *Cololejeuneatrichomanis* (Gottsche) Steph.

*Lejeuneaherzogii* Mizut. = *L.papilionacea* Prantl

*Lejeuneaimbricata* (Nees) Nees = *Cheilolejeuneatrapezia* (Nees) R.M.Schust. et Kachroo

*Lejeuneainfestans* (Steph.) Mizut. = *L.papilionacea* Prantl

*Lejeunealuteola* (Steph.) Mizut. nom. illeg. ≡ *L.mimula* Hürl.

*Lejeuneamitracalyx* (Eifrig) Mizut. = *L.alata* Gottsche

*Lejeuneaornata* (K.I.Goebel) Schiffn. ≡ *Coluraornata* K.I.Goebel

*Lejeuneapilifera* Tixier nom.illeg. ≡ *L.patriciae* Schäf.-Verw.

*Lejeuneapseudostipulata* Schiffn. ≡ *Cololejeuneapseudostipulata* (Schiffn.) Benedix

*Lejeuneapterota* (Herzog) Mizut. = *L.papilionacea* Prantl

*Lejeuneapunctiformis* Taylor ≡ *Microlejeuneapunctiformis* (Taylor) Steph.

*Lejeuneatamaspocsii* G.E.Lee = *L.pulchriflora* (Pearson) G.E.Lee, Bechteler, Pócs, Schäf.-Verw. et Heinrichs

*Lejeuneazollingeri* (Steph.) Mizut. ≡ *L.mizutanii* Grolle

*Lepicolealoriana* Steph. = *L.rara* (Steph.) Grolle

*Lepidolejeuneaborneensis* (Steph.) R.M.Schust. ≡ *Mohamediaborneensis* (Steph.) R.L.Zhu et L.Shu (Zhu et al. 2019)

*Lepidoziaabietinella* Herzog ≡ *Kurziaabietinella* (Herzog) Grolle

Lepidoziacladorhizavar.macgregorii (Steph.) Herzog = *L.cladorhiza* (Reinw., Blume et Nees) Nees

*Lepidoziagonyotricha* Sande Lac. ≡ *Kurziagonyotricha* (Sande Lac.) Grolle

*Lepidozialongitudinalis* Herzog ≡ *Neolepidozialongitudinalis* (Herzog) E.D.Cooper

*Lepidoziamamillosa* Schiffn. ≡ *Neolepidoziamamillosa* (Schiffn.) E.D.Cooper

*Lepidozianeesii* Lindenb. ≡ *Tricholepidozianeesii* (Lindenb.) E.D.Cooper

*Lepidoziaophiria* Steph. ≡ *Neolepidoziaophiria* (Steph.) E.D.Cooper

*Lepidoziapapulosa* Steph. ≡ *Neolepidoziapapulosa* (Steph.) Fulford et J.Taylor

*Lepidoziatrichocoleoides* Herzog ≡ *Tricholepidoziatrichocoleoides* (Herzog) R.M.Schust.

*Lepidoziawallichiana* Gottsche ≡ *Neolepidoziawallichiana* (Gottsche) Fulford et J Taylor

*Leptolejeuneaelliptica* auct. (Asian plants; non typus) = *L.subacuta* A.Evans (Bechteler et al. 2017, Shu et al. 2021)

Leptolejeuneaellipticasubsp.subacuta (A.Evans) R.M.Schust. ≡ *L.subacuta* A.Evans

*Leptolejeuneapicta* Herzog = *L.amphiophthalma* Zwickel

*Leptolejeunearadiata* (Mitt.) Steph. = *L.maculata* (Mitt.) Schiffn.

*Leptolejeuneaschiffneri* (Schiffn.) Steph. = *L.maculata* (Mitt.) Schiffn.

Leptolejeuneaschiffnerif.angustifolia Herzog = *L.maculata* (Mitt.) Schiffn.

Leptolejeuneaschiffnerif.latifolia Herzog = *L.maculata* (Mitt.) Schiffn.

Leptolejeuneaschiffnerivar.subintegerrima Herzog = *L.maculata* (Mitt.) Schiffn.

*Leptolejeuneaserrulata* Herzog = *L.tripuncta* (Mitt.) Steph. (Shu et al. 2016).

*Leptolejeuneaspinistipula* (Mizut.) Xiao L.He ≡ *Soellaspinistipula* (Mizut.) R.L.Zhu et L.Shu (Shu et al. 2021)

*Leucolejeuneadecurrens* (Steph.) Mizut. ≡ *Cheilolejeuneamizutanii* W.Ye et R.L.Zhu

*Leucolejeunealoriana* (Steph.) Mizut. ≡ *Cheilolejeunealoriana* (Steph.) W.Ye et R.L.Zhu

*Leucolejeuneaxanthocarpa* (Lehm. et Lindenb.) A.Evans ≡ *Cheilolejeuneaxanthocarpa* (Lehm. et Lindenb.) Malombe

*Lobatiriccardialobata* (Schiffn.) Furuki = *L.coronopus* (Steph.) Furuki

*Lophocoleaciliolata* (Nees) L.Söderstr. ≡ *Cryptolophocoleaciliolata* (Nees) L.Söderstr.

*Lophocoleacostata* (Nees) L.Söderstr. ≡ *Cryptolophocoleacostata* (Nees) L.Söderstr.

*Lophocoleamassalongoana* Schiffn. ≡ *Cryptolophocoleamassalongoana* (Schiffn.) L.Söderstr.

*Lopholejeuneacranstonii* Steph. = *L.eulopha* (Taylor) Schiffn.

*Lopholejeuneajavanica* (Nees) Schiffn. = *L.nigricans* (Lindenb.) Schiffn.

*Lopholejeunealatialata* Mizut. = *L.zollingeri* (Steph.) Schiffn.

*Lopholejeuneanicobarica* Steph. = *L.eulopha* (Taylor) Schifnn.

*Lopholejeuneanipponica* Horik = *L.zollingeri* (Steph.) Schiffn.

*Makednothallusobtusidens* Herzog = *Jenseniadecipiens* (Mitt.) Grolle

*Marsupellaintegra* N.Kitag. = *Gymnomitrionsubintegrum* (S.W.Arnell) Váňa

*Marsupellarevoluta* (Nees) Dumort. ≡ *Gymnomitrionrevolutum* (Nees) H.Philib.

*Marsupellasubintegra* S.W.Arnell ≡ *Gymnomitrionsubintegrum* (S.W.Arnell) Váňa

*Mastigobryumbancanum* Sande Lac. ≡ *Acromastigumbancanum* (Sande Lac.) A.Evans

*Mastigobryumborneense* De Not. = *Bazzaniafallax* (Sande Lac.) Schiffn.

*Mastigobryumbrotheri* Steph. ≡ *Acromastigumbrotheri* (Steph.) A.Evans

*Mastigobryumcalcaratum* Sande Lac. ≡ *Bazzaniacalcarata* (Sande Lac.) Schiffn.

*Mastigobryumcincinnatum* De Not. ≡ *Bazzaniacincinnata* (De Not.) Trevis.

*Mastigobryumconcinnum* De Not. = *Bazzaniaintermedia* (Gottsche et Lindenb.) Trevis.

*Mastigobryumcrenatistipulum* Steph. = *Bazzaniamanillana* (Steph.) Steph.

*Mastigobryumduplex* De Not. = *Bazzaniainvolutiformis* (De Not.) Trevis.

*Mastigobryumechinatiforme* De Not. ≡ *Acromastigumechinatiforme* (De Not.) A.Evans

*Mastigobryumelegantulum* De Not. = *Acromastiguminaequilaterum* (Lehm. et Lindenb.) A.Evans

*Mastigobryumerosum* δ *pulopenangense* Lindenb. et Gottsche ≡ Bazzaniaerosavar.pulopenangensis (Lindenb. et Gottsche) Schiffn.

*Mastigobryumferox* De Not. = *Bazzaniaharpago* (De Not.) Schiffn.

*Mastigobryumfimbriatum* Steph. ≡ *Acromastigumfimbriatum* (Steph.) A.Evans

*Mastigobryumfleischeri* Steph. ≡ *Bazzaniafleischeri* (Steph.) Abeyw.

*Mastigobryumharpago* De Not. ≡ *Bazzaniaharpago* (De Not.) Schiffn.

*Mastigobryuminaequitextum* (Steph.) Steph. ≡ *Bazzaniainaequitexta* Steph.

*Mastigobryuminsigne* De Not. ≡ *Bazzaniainsignis* (De Not.) Trevis.

Mastigobryumintermediumvar.sarawakianum De Not. ≡ Bazzaniaintermediavar.sarawakiana (De Not.) Schiffn.

*Mastigobryuminvolutiforme* De Not. ≡ *Bazzaniainvolutiformis* (De Not.) Trevis.

*Mastigobryumlinganum* De Not. ≡ *Acromastigumlinganum* (De Not.) A.Evans

*Mastigobryumlinguiforme* Sande Lac. ≡ *Bazzanialinguiformis* (Sande Lac.) Trevis.

*Mastigobryumloricatum* (Reinw., Blume et Nees) Gottsche, Lindenb. et Nees ≡ *Bazzanialoricata* (Reinw., Blume et Nees) Trevis.

*Mastigobryumlowii* Sande Lac. ex Steph. ≡ *Bazzanialowii* (Steph.) Schiffn.

*Mastigobryummalaccense* Steph. ≡ *Bazzaniamalaccensis* (Steph.) Tixier

*Mastigobryummuricatulum* Herzog = *Bazzaniahorridula* Schiffn.

*Mastigobryumnatunense* Steph. = *Bazzaniaparadoxa* (Sande Lac.) Steph.

*Mastigobryumnotarisii* (De Not.) Steph. nom. illeg. = *Acromastiguminaequilaterum* (Lehm. et Lindenb.) A.Evans

*Mastigobryumpulvinulatum* De Not. ≡ *Mastigopelmapulvinulatum* (De Not.) Grolle

*Mastigobryumrecurvum* (Mont.) Lindenb. ≡ *Bazzaniarecurva* (Mont.) Trevis.

Mastigobryumrecurvumvar.pallens De Not. = *Bazzaniarecurva* (Mont.) Trevis.

*Mastigobryumrepandistipulum* Steph. = *Bazzaniatridens* (Reinw., Blume et Nees) Trevis.

*Mastigobryumsubtile* Sande Lac. ≡ *Bazzaniasubtilis* (Sande Lac.) Trevis.

*Mastigobryumvagum* De Not. = *Bazzaniaerosa* (Reinw., Blume et Nees) Trevis.

Mastigobryumvittatumvar.luxurians De Not. ≡ Bazzaniavittatavar.luxurians (De Not.) Schiffn.

*Mastigobryumzollingeri* Lindenb. ≡ *Bazzaniazollingeri* (Lindenb.) Trevis.

*Mastigolejeuneaatypos* Sydow = *Thysananthusrepletus* (Taylor) Sukkharak et Gradst.

*Mastigolejeuneaauriculata* auct. (Asian plants; non typus) = *Thysananthushumilis* (Steph.) Sukkharak et Gradst.

*Mastigolejeuneagradsteinii* Sukkharak ≡ *Thysananthusgradsteinii* (Sukkharak) Sukkharak et Gradst.

*Mastigolejeuneahumilis* (Gottsche) Schiffn. ≡ *Thysananthushumilis* (Gottsche) Sukkharak et Gradst.

*Mastigolejeuneaindica* Steph. ≡ *Thysananthusindicus* (Steph.) Sukkharak et Gradst.

*Mastigolejeunealigulata* (Lehm. et Lindenb.) Schiffn. ≡ *Thysananthusligulatus* (Lehm. et Lindenb.) Sukkharak et Gradst.

*Mastigolejeunearecondita* (Steph.) Mizut. ≡ *Thysananthusreconditus* (Steph.) Sukkharak et Gradst.

*Mastigolejeunearecurvifolia* Mizut. ≡ *Thysananthushumilis* (Gottsche) Sukkharak et Gradst.

*Mastigolejeunearepleta* (Taylor) A.Evans ≡ *Thysananthusrepletus* (Taylor) Sukkharak et Gradst.

*Mastigolejeuneatruncata* Mizut. ≡ *Thysananthustruncatus* (Mizut.) Sukkharak et Gradst.

*Mastigolejeuneavirens* (Ångstr.) Steph. ≡ *Thysananthusvirens* (Ångstr.) Sukkharak et Gradst.

*Mastigopelmabilobum* Herzog = *Mastigopelmapulvinulatum* (De Not.) Grolle

*Megaceroscelebensis* Steph. = *Megacerosflagellaris* (Mitt.) Steph.

*Metahygrobiellaacuminata* (Herzog) R.M.Schust. ≡ *Cephaloziaacuminata* (Herzog) R.M.Schust.

*Metahygrobiellaacutiloba* Inoue ≡ *Cephaloziaacutiloba* Inoue

*Metahygrobiellamollusca* (De Not.) R.M.Schust. ≡ *Cephaloziamollusca* (De Not.) R.M.Schust.

*Metahygrobiellastolonacea* (Herzog) R.M.Schust. ≡ *Cephaloziastolonacea* (Herzog) R.M.Schust.

*Metzgeriaborneensis* Kuwah. = *M.leptoneura* Spruce

Metzgeriaconjugatasubsp.japonica (S.Hatt.) Kuwah. = *M.lindbergii* Schiffn.

*Metzgeriadecipiens* (C.Massal.) Schiffn. = *M.ciliata* Raddi

*Metzgeriahamata* Lindb. = *M.leptoneura* Spruce

*Metzgeriamolokaiensis* Kuwah. = *M.furcata* (L.) Corda

*Metzgeriapapulosa* Steph. = *M.leptoneura* Spruce

*Metzgeriapectinata* Steph. = *M.lindbergii* Schiffn.

*Metzgeriasandei* Schiffn. = *M.leptoneura* Spruce

*Metzgeriasharpii* Kuwah. = *M.leptoneura* Spruce

*Metzgeriathomeensis* Steph. = *M.leptoneura* Spruce

*Metzgeriopsispusilla* K.I.Goebel = *Cololejeuneametzgeriopsis* (K.I.Goebel) Gradst., R.Wilson, Ilk.-Borg. et Heinrichs

*Microlejeuneacucullata* (Reinw., Blume et Nees) J.B.Jack et Steph. ≡ *Metalejeuneacucullata* (Reinw., Blume et Nees) Grolle

*Microlejeuneagracillima* (Mitt.) Steph. = *M.lunulatiloba* Horik.

*Microlejeuneasundaica* Steph. = *Metalejeuneacucullata* (Reinw., Blume et Nees) Grolle

*Neohattoriaperversa* (Steph.) R.M.Schust. = Frullaniajunghuhnianavar.tenella (Sande Lac.) Grolle et S.Hatt.

*Notoscyphusparoicus* Schiffn. = *N.lutescens* (Lehm. et Lindenb.) Mitt.

*Otolejeuneasemperiana* (Steph.) Grolle ≡ *Allorgellasemperiana* (Steph.) Bechteler, G.E.Lee, Schäf.-Verw. et Heinrichs

*Pallaviciniafistulosa* Steph. = *Pallaviciniaambigua* (Mitt.) Steph.

*Phaeocerosfoveatus* J.Haseg. ≡ *Phaeomegacerosfoveatus* (J.Haseg.) J.C.Villarreal

*Phragmicomaligulata* (Lehm. et Lindenb.) Gottsche, Lindenb. et Nees ≡ *Thysananthusligulatus* (Lehm. et Lindenb.) Sukkharak et Gradst.

*Phragmicomapulopenangensis* Gottsche ≡ *Schiffneriolejeuneapulopenangensis* (Gottsche) Gradst.

*Phragmicomatumida* (Nees) Nees et Mont. ≡ *Schiffneriolejeuneatumida* (Nees) Gradst.

*Physiotiummyriangium* De Not. = *Pleuroziaacinosa* (Mitt.) Trevis.

*Placolejeuneasubarrhyncha* Herzog = *Cheilolejeuneatrapezia* (Nees) R.M.Schust. et Kachroo

*Plagiochilaaccedens* Steph. = *P.korthalsiana* Molk.

*Plagiochilacameronensis* Inoue = *P.hampeana* Gottsche

*Plagiochiladendroides* (Nees) Lindenb. ≡ *Chiastocaulondendroides* (Nees) Carl

*Plagiochilaelegantissima* Herzog nom. illeg. = *P.blepharophora* (Nees) Lindenb.

*Plagiochilaestipulata* Steph. = *P.blepharophora* (Nees) Lindenb.

*Plagiochilafraseri* Steph. = *P.teysmannii* Sande Lac.

*Plagiochilahottae* Inoue = *P.perserrata* Herzog

*Plagiochila inﬁrma* (Sande Lac.) Sande Lac. = *P.javanica* (Sw.) Nees et Mont

*Plagiochilalaxissima* Schiffn. = *P.bicornuta* Steph.

*Plagiochilalinguifolia* De Not. ≡ *Denotarisialinguifolia* (De Not.) Grolle

*Plagiochilalobulata* Schiffn. = *P.bantamensis* (Reinw., Blume et Nees) Mont

*Plagiochilalongifolia* Steph. = *P.clavatosaccata* Steph.

*Plagiochilamutabilis* De Not. = *P.bantamensis* (Reinw., Blume et Nees) Mont

*Plagiochilaopposita* Dumort. ≡ *Chiastocaulonoppositum* (Reinw., Blume et Nees) S.D.F.Patzak, M.A.M Renner, Schäf.-Verw. et Heinrichs

*Plagiochilapachycephala* De Not. ≡ *Chiastocaulonpachycephalum* (De Not.) Herzog

*Plagiochilarichardsii* Herzog = *P.bantamensis* (Reinw., Blume et Nees) Mont.

*Plagiochilasambusana* (Steph.) Beauverd = *P.kurzii* Steph.

Plagiochilasandeif.remotidens Herzog = *P.sandei* Dozy

*Plagiochilasingularis* Schiffn. ≡ *Dinckleriasingularis* (Schiffn.) M.A.M.Renner, Schäf.-Verw. et Heinrichs

*Plagiochilatridens* Steph. = *P.ungarangana* Sande Lac.

*Plagiochilatsutomui* Inoue = *P.ungarangana* Sande Lac.

*Plagiochilavanikorensis* Steph. = *P.bantamensis* (Reinw., Blume et Nees) Mont

*Plagiochilionbraunianum* (Nees) S.Hatt. ≡ *Chiastocaulonbraunianum* (Nees) S.D.F.Patzak, M.A.M Renner, Schäf.-Verw. et Heinrichs

*Plagiochilionoppositum* (Reinw., Blume et Nees) S.Hatt. ≡ *Chiastocaulonoppositum* (Reinw., Blume et Nees) S.D.F.Patzak, M.A.M Renner, Schäf.-Verw. et Heinrichs

*Plagiochilionpachycephalum* (De Not.) Inoue ≡ *Chiastocaulonpachycephalum* (De Not.) Herzog

*Plagiochiliontheriotianum* (Steph.) Inoue ≡ *Chiastocaulontheriotianum* (Steph.) S.D.F.Patzak, M.A.M Renner, Schäf.-Verw. et Heinrichs

*Plectocoleaparabolica* Herzog = *Solenostomatetragonum* (Lindenb.) Váňa et D.G.Long

*Plectocoleatruncata* (Nees) Herzog ≡ *Solenostomatruncatum* (Nees) Váňa et D.G.Long

Pleuroziagiganteavar.borneensis (De Not.) Schiffn. = *P.gigantea* (F.Weber) Lindb.

Porellaacutifoliasubsp.latior S.Hatt. = P.caespitanssubsp.latior (S.Hatt.) S.Hatt.

Porellaacutifoliavar.elbertii (Steph.) S.Hatt. = *P.acutifolia* (Lehm. et Lindenb.) Trevis.

Porellaacutifoliavar.johannis-winkleri (Herzog) S.Hatt. = *P.acutifolia* (Lehm. et Lindenb.) Trevis.

*Porellajohannis-winkleri* (Herzog) S.Hatt. = *P.acutifolia* (Lehm. et Lindenb.) Trevis.

*Prionolejeuneaungulata* Herzog = *Lejeuneaapiculata* Sande Lac.

Pseudolepicoleatrolliisubsp.andoi (R.M.Schust.) S.Hatt. et Mizut. = *P.andoi* (R.M.Schust.) S.Hatt. et Mizut.

*Ptychanthuspycnocladus* Taylor ≡ *Acrolejeuneapycnoclada* (Taylor) Schiffn.

*Ptychanthussulcatus* (Nees) Nees ≡ *Spruceanthussulcatus* (Nees) Gradst.

*Ptychanthustumidus* Nees ≡ *Schiffneriolejeuneatumida* (Nees) Gradst.

*Ptychocoleuscoroniformis* T.Kodama et N.Kitag. = *Acrolejeuneaarcuata* (Nees) Grolle et Gradst.

*Ptychocoleuscranstonii* Steph. = *Schiffneriolejeuneapulopenangensis* (Gottsche) Gradst.

*Ptychocoleusfertilis* (Reinw., Blume et Nees) Trevis. ≡ *Acrolejeuneafertilis* (Reinw., Blume et Nees) Schiffn.

*Ptychocoleushaskarlianus* (Gottsche) Steph. = *Schiffneriolejeuneatumida* (Nees) Gradst.

*Ptychocoleuslongispicus* Steph. = *Schiffneriolejeuneanymannii* (Steph.) Gradst. et Terken.

*Ptychocoleuspulopenangensis* (Gottsche) Trevis. ≡ *Schiffneriolejeuneapulopenangensis* (Gottsche) Gradst.

*Ptychocoleuspycnocladus* (Taylor) Steph. ≡ *Acrolejeuneapycnoclada* (Taylor) Schiffn.

*Ptychocoleussarawakensis* Steph. = *Schiffneriolejeuneatumida* (Nees) Gradst.

*Ptychocoleussquarrosifolius* Steph. = *Schiffneriolejeuneatumida* (Nees) Gradst.

*Ptychocoleustjibodensis* Verd. ≡ *Acrolejeuneatjibodensis* (Verd.) Grolle et Gradst.

*Ptychocoleustridens* Steph. = *Schiffneriolejeuneapulopenangensis* (Gottsche) Gradst.

*Ptychocoleuswichurae* (Schiffn.) Steph. = *Acrolejeuneafertilis* (Reinw., Blume et Nees) Schiffn.

*Pycnolejeuneaangulistipa* Steph. ≡ *Tuyamaellaangulistipa* (Steph.) R.M.Schust. et Kachroo

*Pycnolejeuneaappendiculata* Herzog = *Tuyamaellaserratistipa* S.Hatt.

*Pycnolejeuneaarietina* Tixier = *Cheilolejeuneaceylanica* (Gottsche) R.M.Schust. et Kachroo

Pycnolejeuneabadiaf.parvistipa Herzog = *Lepidolejeuneabidentula* (Steph.) R.M.Schust.

*Pycnolejeuneabancana* Steph. = *P.contigua* (Nees) Grolle

*Pycnolejeuneabidentula* Steph. ≡ *Lepidolejeuneabidentula* (Steph.) R.M.Schust.

*Pycnolejeuneaborneensis* Steph. ≡ *Mohamediaborneensis* (Steph.) R.L.Zhu et L.Shu (Zhu et al. 2019).

*Pycnolejeuneaceylanica* (Gottsche) Schiffn. = *Cheilolejeuneaceylanica* (Gottsche) R.M.Schust. et Kachroo

*Pycnolejeuneacorticola* Steph. = *Lepidolejeuneabidentula* (Steph.) R.M.Schust.

*Pycnolejeuneadecurvifolia* (Steph.) Steph. = *Lepidolejeuneabidentula* (Steph.) R.M.Schust.

*Pycnolejeuneaexcisula* Steph. = *Cheilolejeuneaincisa* (Gottsche) R.M.Schust.

*Pycnolejeuneaflavida* (Mitt.) Steph. ≡ *Lejeuneaflavida* Mitt.

*Pycnolejeuneagigantea* Steph. ≡ *Cheilolejeuneagigantea* (Steph.) R.M.Schust. et Kachroo

*Pycnolejeuneaimbricata* (Nees) Schiffn. = *Cheilolejeuneatrapezia* (Nees) R.M.Schust. et Kachroo

*Pycnolejeuneaintegristipula* J.B.Jack et Steph. ≡ *Lepidolejeuneaintegristipula* (J.B.Jack et Steph.) R.M.Schust.

*Pycnolejeuneakinabaluensis* T.Kodama = *P.sphaeroides* (Sande Lac.) J.B.Jack et Steph.

*Pycnolejeuneamalaccensis* G.Hoffm. ≡ *Cheilolejeuneamalaccensis* (G.Hoffm.) Xiao L.He

*Pycnolejeuneameyeniana* (Nees, Lindenb. et Gottsche) Steph. = *Cheilolejeuneatrapezia* (Nees) R.M.Schust. et Kachroo

Pycnolejeuneameyenianavar.ligulata G.Hoffm. = *Cheilolejeuneatrapezia* (Nees) R.M.Schust. et Kachroo

*Pycnolejeuneamicholitzii* Steph. ≡ *Cheilolejeuneamicholitzii* (Steph.) R.M.Schust. et Kachroo

*Pycnolejeuneamolaris* Mizut. = *P.cavistipula* (Steph.) Mizut.

*Pycnolejeuneanicobarica* Steph. = *Lepidolejeuneabidentula* (Steph.) R.M.Schust.

*Pycnolejeuneapunctata* Steph. = *Lepidolejeuneabidentula* (Steph.) R.M.Schust.

*Pycnolejeuneaspinistipula* Mizut. ≡ *Soellaspinistipula* (Mizut.) R.L.Zhu et L.Shu (Shu et al. 2021)

*Pycnolejeuneautriculata* Steph. ≡ *Lejeuneautriculata* (Steph.) Mizut.

*Pycnolejeuneaventricosa* (Schiffn.) P.Syd. ≡ *Cheilolejeuneaventricosa* (Schiffn.) Xiao L.He

*Radulaapiculata* Steph. = *R.anceps* Sande Lac.

*Radulaindica* Steph. = *R.tabularis* Steph.

*Radulalaciniata* Herzog = *R.anceps* Sande Lac.

*Radulamiqueliana* Taylor = *R. retroﬂexa* Taylor

*Radulanovae-guineae* Steph. = *R.formosa* (Spreng.) Nees

*Radularetroﬂexavar.fauciloba* (Steph.) K.Yamada = *R.retroﬂexa* Taylor

*Radulasubpallens* Steph. = *R.ventricosa* Steph.

*Radulayangii* (B.Y.Yang) K.Yamada = *R.javanica* Gottsche

Rebouliahemisphaericavar.javanica (Nees) Schiffn. = R.hemisphaericasubsp.australis R.M.Schust.

*Rhaphidolejeuneacyclops* (Sande Lac.) Herzog ≡ *Drepanolejeuneacyclops* (Sande Lac.) Grolle et R.L.Zhu

*Rhaphidolejeunealongicruris* (Steph.) Herzog ≡ *Drepanolejeunealongicruris* (Steph.) Grolle et R.L.Zhu

*Rhaphidolejeuneaspicata* (Steph.) Grolle ≡ *Drepanolejeuneaspicata* (Steph.) Grolle et R.L.Zhu

*Riccardialobata* Schiffn. = *Lobatiriccardiacoronopus* (Steph.) Furuki

Riccardialobataf.gigantea Herzog nom. inval. = *Lobatiriccardiacoronopus* (Steph.) Furuki

*Riccardiapinguis* (L.) Gray ≡ *Aneurapinguis* (L.) Dumort.

*Riccardiaridleyi* Schiffn. = *R.elata* (Steph.) Schiffn.

*Riccardiascabra* Schiffn. = *R.crassa* (Schwägr.) C.Massal.

*Riccardiatenuicostata* Schiffn. = *R.inconspicua* (Steph.) Reeb et Bardat

*Saccogynamuricella* (De Not.) Mitt. ≡ *Saccogynidiummuricellum* (De Not.) Grolle

*Saccogynarigidula* (Nees) Schiffn. ≡ *Saccogynidiumrigidulum* (Nees) Grolle

*Saccogynidiumiwatsukii* S.Hatt. ≡ *Heteroscyphusiwatsukii* (S.Hatt.) Piippo

*Saccogynidiumjugatum* (Mitt.) Grolle = *S.rigidulum* (Nees) Grolle

Scapaniajavanicaf.amplifolia Herzog nom. inval. = *S.javanica* Gottsche

*Schistochilaaligeriformis* (De Not.) Schiffn. = *S.aligera* (Nees et Blume) J.B.Jack et Steph.

*Schistochilaphilippinensis* (Mont.) Steph. = *S.aligera* (Nees et Blume) J.B.Jack et Steph.

*Schistochilawallisii* J.B.Jack et Gottsche = *S.blumei* (Nees) Trevis.

*Schistochilawrayana* Steph. = *S.acuminata* Steph.

Sendtneradicladosvar.borneensis De Not. = *Mastigophoradiclados* (F.Weber) Nees

*Southbyagrollei* N.Kitag. = *S.organensis* Herzog

*Sphenolobopsiskitagawae* R.M.Schust. = *Sphenolobopsispearsonii* (Spruce) R.M.Schust.

*Spruceanthusmarianus* sensu Mizut. (non typus) = *S.planiuscula* (Mitt.) X.Q.Shi, R.L.Zhu et Gradst. (Shi et al. 2015).

*Steereaclemensiana* (Verd.) S.Hatt. ≡ *Frullaniaclemensiana* Verd.

*Steereamastigophoroides* S.Hatt. et Kamim. = *Frullaniaclemensiana* Verd.

*Stenolejeuneaapiculata* (Sande Lac.) R.M.Schust. ≡ *Lejeuneaapiculata* Sande Lac.

*Stenorrhipisrhizomatica* Herzog = *Kymatocalyxrhizomaticus* (Herzog) Gradst. et Váňa

*Stictolejeunearichardsii* Herzog = *S.balfourii* (Mitt.) E.W.Jones

*Strepsilejeuneaocclusa* Herzog ≡ *Cheilolejeuneaocclusa* (Herzog) T.Kodama et N.Kitag.

*Symphyogynopsisfilicum* (Nadeaud) Grolle = *S.gottscheana* (Mont. et Nees) Grolle

*Syzygiellavariegata* (Lindenb.) Spruce = *S.securifolia* (Nees) Inoue

*Taeniolejeuneaperaffinis* (Schiffn.) Zwickel ≡ *Cololejeuneaperaffinis* (Schiffn.) Schiffn.

*Taxilejeuneaalbescens* Steph. ≡ *Lejeuneaalbescens* (Steph.) Mizut.

*Taxilejeuneacompressiuscula* Steph. ≡ *Lejeuneacompressiuscula* Steph. (Lee et al. 2018)

*Taxilejeunealumbricoides* (Nees) Steph. ≡ *Lejeunealumbricoides* (Nees) Nees

*Taxilejeunealuteola* (Steph.) Eifrig ≡ *Lejeuneamimula* Hürl.

*Taxilejeuneapatersonii* (Steph.) Eifrig = *Lejeunealeratii* (Steph.) Mizut.

*Taxilejeuneasordida* (Nees) Eifrig ≡ *Lejeuneasordida* (Nees) Nees

*Taxilejeuneaumbilicata* (Nees) J.B.Jack et Steph. ≡ *Lejeuneaumbilicata* (Nees) Nees

*Telaranealongitudinalis* (Herzog) R.M.Schust. ≡ *Neolepidozialongitudinalis* (Herzog) E.D.Cooper

*Telaraneaneesii* (Lindenb.) Fulford ≡ *Tricholepidozianeesii* (Lindenb.) E.D.Cooper

*Telaraneaoctoloba* Del Ros. ≡ *Tricholepidoziaoctoloba* (Del Ros.) E.D.Cooper

*Telaraneaophiria* (Gottsche) J.J.Engel et G.L.Merr. ≡ *Neolepidoziaophiria* (Gottsche) E.D.Cooper

*Telaraneaquadriseta* (Steph.) J.J.Engel et G.L.Merr. ≡ *Tricholepidoziaquadriseta* (Steph.) E.D.Cooper

*Telaraneasemperiana* (Steph.) Del Ros. ≡ *Tricholepidoziasemperiana* (Steph.) E.D.Cooper

*Telaraneatrichocoleoides* (Herzog) R.M.Schust. ≡ *Tricholepidoziatrichocoleoides* (Herzog) R.M.Schust.

*Thysananthusborneensis* Steph. = *T.gottschei* (J.B.Jack et Steph.) Steph.

Thysananthusfruticosusf.pendulus Herzog = *T.fruticosus* (Lindenb. et Gottsche) Schiffn.

*Thysananthuslaceratus* Steph. = *T.convolutus* Lindenb.

*Thysananthusminor* Verd. = *T.spathulistipus* (Reinw., Blume et Nees) Lindenb.

*Thysananthusplanus* Sande Lac. = *T.retusus* (Reinw., Blume et Nees) B.M.Thiers et Gradst.

*Thysananthusrichardsianus* Verd. = *T.aculeatus* Herzog

Thysananthusspathulistipusf.borneensis (Herzog) Herzog = *T.spathulistipus* (Reinw., Blume et Nees) Lindenb.

*Trichocoleamerrillana* Steph. ≡ *Leiomitramerrillana* (Steph.) T.Katag.

*Trocholejeuneacrassicaulis* (Steph.) Mizut. ≡ *Acrolejeuneacrassicaulis* (Steph.) J.Wang bis et Gradst.

*Tylimanthussaccatus* (Hook.) Carrington et Pearson ≡ *Acrobolbussaccatus* (Hook.) Briscoe

*Xenolejeuneaceylanica* (Steph.) Tixier nom. inval. ≡ *Cheilolejeuneaceylanica* (Gottsche) R.M.Schust. et Kachroo

*Xenolejeuneafalsinervis* (Sande Lac.) Tixier nom. inval. ≡ *Cheilolejeuneafalsinervis* (Sande Lac.) R.M.Schust. et Kachroo

*Xenolejeunealongiloba* (Steph.) Tixier nom. inval. = *Cheilolejeuneatrapezia* (Nees) R.M.Schust. et Kachroo

*Xenolejeuneameyeniana* (Steph.) Tixier nom. inval. ≡ *Cheilolejeuneatrapezia* (Nees) R.M.Schust. et Kachroo

*Zoopsisargentea* (Hook.f. et Taylor) Gottsche, Lindenb. et Nees = *Z.liukiuensis* Horik.

*Zoopsisrigida* Pearson = *Z.setigera* K.I.Goebel
